# Folding within Frameworks: Confinement in Zr-MOFs Reshapes Enzyme Structure and Catalytic Activity

**DOI:** 10.1021/jacs.6c11124

**Published:** 2026-09-16

**Authors:** Siene Swinnen, Maxim Lox, Marika Di Berto Mancini, Kilian Declerck, Francisco de Azambuja, Tatjana N. Parac-Vogt, Bettina Baumgartner

**Affiliations:** † Department of Chemistry, 26657 KU Leuven, Celestijnenlaan 200F, Leuven 3001, Belgium; ‡ Van’t Hoff Institute for Molecular Sciences, 1234 University of Amsterdam, Science Park 904, Amsterdam 1098 XH, The Netherlands

## Abstract

Enhancing the robustness of functional proteins remains a central challenge in biotechnology, with implications for catalysis, pharmaceuticals, and industrial synthesis. Enzyme immobilization in porous materials such as metal–organic frameworks (MOFs) is widely used to enhance enzyme stability; however, the structural state of proteins within these environments is poorly understood and is often assumed to remain largely unchanged. Since enzyme functionality is closely linked to its 3D conformation, the lack of detailed structural information makes the design of enzyme@porous systems largely empirical. In this work, we demonstrate that *in situ* attenuated total reflectance infrared spectroscopy is a powerful tool for monitoring protein adsorption and confinement in the Zr-based MOF NU-1000. By tracking characteristic amide bands, we monitor changes in protein vibrational signatures that reflect alterations in protein structure and local environment during interaction with the framework. Our results reveal that MOFs are not passive hosts but can induce pronounced perturbations in the protein structure upon adsorption and confinement. We identified a framework-sensitive spectroscopic signature associated with protein uptake into the MOF pore environment and support this assignment through uptake kinetics, diffusion analysis, pore-size controls, and protease accessibility experiments. Protein uptake is governed not only by size compatibility but also by electrostatic interactions, ionic strength, and protein conformational state. Importantly, these immobilization- and confinement-associated structural perturbations correlate with changes in catalytic activity: enhanced catalytic activity for dynamically perturbed proteins and reduced activity for structurally constrained systems. These findings support a relationship between protein–MOF interactions, structural perturbation, and enzymatic function and provide guidelines for tuning protein behavior in MOF-based biocatalysis, separations, and sensing applications.

## Graphical abstract

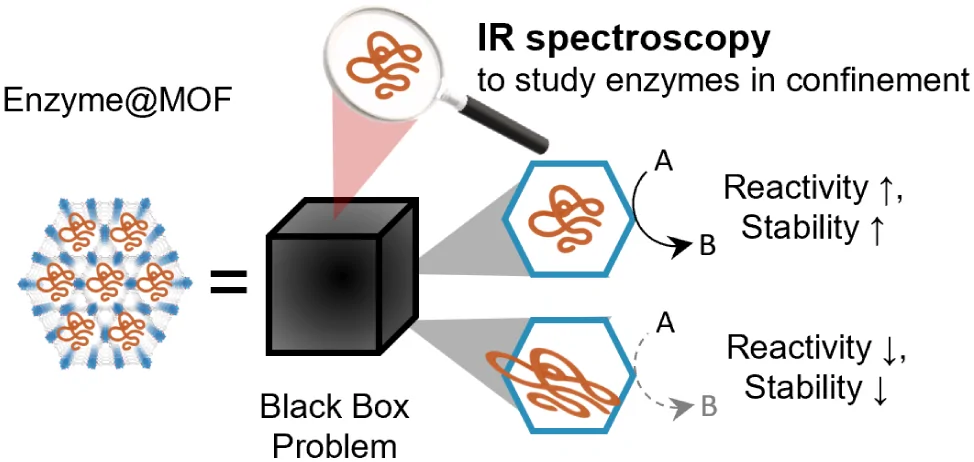

## Introduction

Enzymes are highly selective catalysts, yet their practical application is often limited by poor stability under narrow operating conditions. Encapsulation within solid supports has emerged as a powerful strategy for enhancing enzyme stability, recyclability, and functional robustness under non-native environments. A wide range of solid and porous materials, including silica, polymers,^1–7^ and metal oxides,^8–11^ has been explored as enzyme supports. However, these materials often lack the structural precision and tunability required to control biomolecular interactions at the molecular level. In this context, the long-established concept of enzyme immobilization has gained renewed momentum with the emergence of porous framework materials that offer unprecedented control over the local environment experienced by biomolecules.^12–15^

Metal–organic frameworks (MOFs) are hybrid organic–inorganic architectures composed of metal nodes and organic linkers, forming highly ordered, crystalline structures that can be designed to contain large and tunable pores capable of accommodating entire proteins (Figure 1).^16,17^ Encapsulation within the pores prevents aggregation, thereby elongating protein stability. Several studies have demonstrated that enzymes can be accommodated in the pores of MOFs,^18–20^ often resulting in improved catalytic activity, stability, and recyclability.^13,16,21–24^ For example, encapsulation of catalase in MAF-7 enhances H_2_O_2_ decomposition and preserves enzymatic activity under otherwise denaturing conditions.^25^ However, beyond serving as passive hosts, MOFs can also exhibit intrinsic catalytic activity toward biologically relevant phosphoester and peptide bonds. In particular, Zr-based MOFs, owing to the high Lewis acidity of Zr­(IV), catalyze hydrolytic reactions ranging from small molecules (e.g., chemical warfare agents or pesticides^26–29^ ) to biomacromolecules such as proteins,^30–35^ as demonstrated in studies by our group.^36–38^ This dual functionality, i.e., simultaneously acting as host and catalyst, makes MOF–protein systems uniquely versatile but also fundamentally complex.

**Figure 1:**
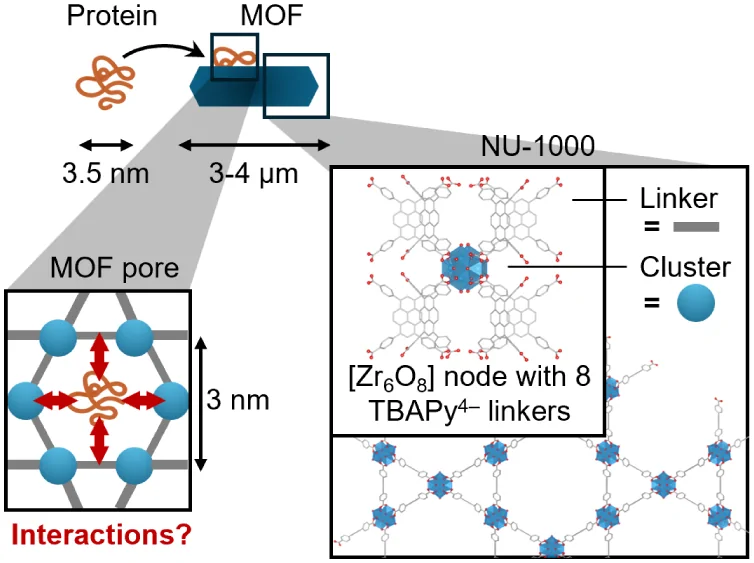
Schematic representation of protein adsorption onto a MOF. A magnified highlight shows the structure of Zr-MOF NU-1000, with a single metal node connected to eight 1,3,6,8-(*p*-benzoate)­pyrene (TBAPy^4–^) linkers. Color scheme: Zr (teal), O (red), C (gray). Hydrogens omitted for clarity.

Because enzyme function is largely dependent on its conformation, understanding how confinement and interactions within MOFs affect protein structure is critical. However, despite increasing interest in enzyme@MOF systems, establishing general design principles remains challenging and largely empirical. Even for relatively simple systems, such as amino acids and peptides, our recent work has shown that MOF–biomolecule interactions depend on a multitude of factors.^39,40^ For whole proteins, this complexity is further amplified by their size, structural flexibility, and sensitivity to environmental conditions. As a result, a comprehensive molecular-level understanding of how MOFs influence protein structure and function is still lacking, particularly under realistic conditions.^5,41^

In addition to the complexity of the system, another major challenge is the limited structural information provided by currently availableexperimental techniques. Methods such as isothermal titration calorimetry (ITC),^42,43^ small-angle X-ray scattering (SAXS),^44^ small-angle neutron scattering (SANS),^45^ Bradford assay,^46^ or confocal fluorescence microscopy (CFM)^47^ provide valuable information on adsorption kinetics and protein loading but offer limited structural insight into the protein upon interaction with the MOF. In particular, they generally cannot reveal structural differences between proteins in solution, those adsorbed on the external surface, and those confined within the pores, nor do they enable simultaneous characterization of both the MOF host and protein guest under relevant conditions. Consequently, current studies are often biased toward a material-centered perspective, leaving the structural characterization of proteins within MOFs insufficiently understood.

Among the spectroscopic techniques capable of probing molecules in confinement,^48^ infrared (IR) spectroscopy represents a particularly powerful yet underutilized approach for studying MOF–biomolecule interactions.^49–51^ In attenuated total reflectance (ATR) mode, IR absorption takes place within an evanescent wave generated at the crystal–sample interface upon total internal reflection of the incident IR beam (Figure 2A). This field penetrates approximately 200–800 nm into the sample, reducing solvent background while enabling simultaneous probing of the MOF structure, proteins in solution, surface-adsorbed species, and proteins confined within the pores. Furthermore, structural alterations of proteins induced by environmental changes (e.g., pH, temperature, pressure, solvents, or confinement) can be tracked via characteristic vibrational bands of proteins, most prominently the amide I and II modes (Figure 2B). The amide I band (1610–1695 cm^–1^) arises mainly from C=O stretching, with minor contributions from N–H in-plane bending and out of phase C–N stretching of the peptide bond, while the amide II band (∼1550 cm^–1^) originates from a combination of N–H in-plane bending and C–N stretching. Both are highly sensitive to changes in secondary structure. Variations in hydrogen bonding patterns and dipole interactions associated with α-helices, β-sheets, turns, and disordered regions lead to distinct spectral signatures.^50^ Upon adsorption onto a solid surface, both amide bands exhibit broadening and the ratio of amide band heights changes from amide I > amide II to amide I < amide II, resulting in markedly different IR spectra compared to those observed in solution (Figure 2C).^50^ Altogether, this sensitivity makes ATR-FTIR spectroscopy uniquely suited to probe protein conformational changes within MOFs and for investigating protein adsorption, interaction dynamics, and reaction progress in enzyme@MOF systems under biologically relevant conditions.^25,52^

**Figure 2:**
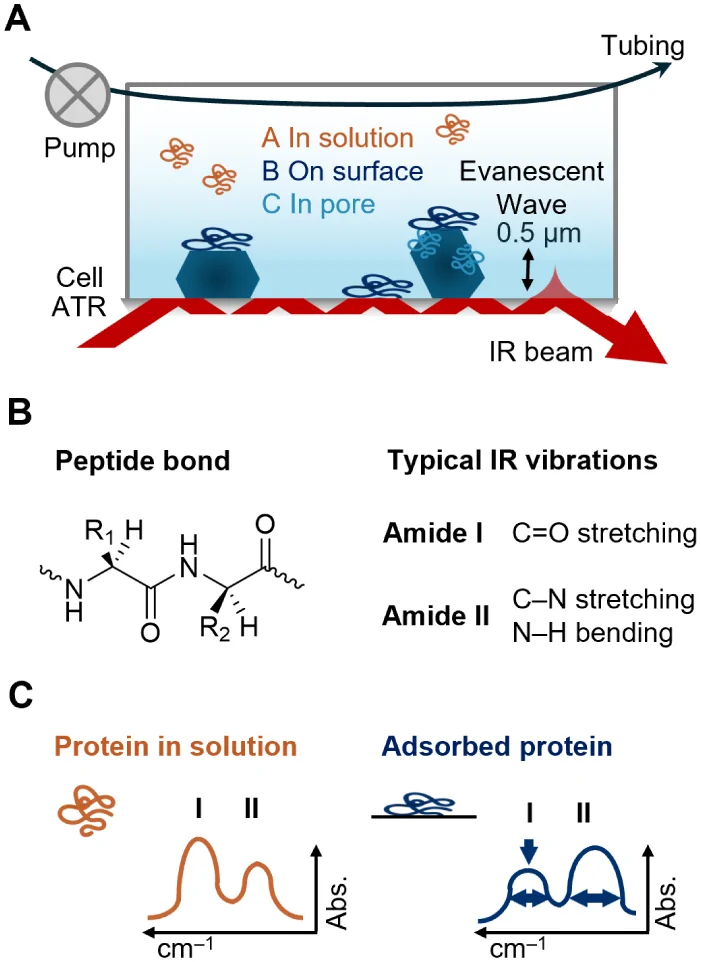
(A) Schematic representation of the IR spectroscopic setup used in this study. (B) Peptide bond structure and resulting typical bond vibrations. (C) Schematic representation of typical protein IR spectra.

Building on these spectroscopic principles, we present a systematic investigation of protein adsorption onto established Zr-based MOFs by using *in situ* ATR-FTIR spectroscopy. Our study includes the widely investigated framework NU-1000, which features large 3 nm pore apertures (Figure 1), as well as the microporous UiO-66 and mesoporous PCN-222, allowing us to assess the generality of our findings across MOFs with different pore architectures. Using a series of model proteins with varying sizes, structures, and isoelectric points (pI), we could directly probe protein behavior during confinement. We show that encapsulation is not governed solely by size exclusion but is strongly influenced by electrostatic interactions and the ionic environment. Notably, we identify new protein conformations that emerge exclusively within MOF pores, highlighting confinement as a means of modulating protein structure. Finally, we correlate confinement-induced structural perturbations with enzymatic reactivity, revealing that MOFs can act as tunable environments for controlling protein structure and function.

## Results and Discussion

### Choice of Proteins and Experimental Considerations

To systematically probe MOF–protein interactions across a representative chemical space, we selected cytochrome c (Cyt c), α-lactalbumin (α-Lac), myoglobin (MB), β-lactoglobulin (β-Lac), hemoglobin (HB), ovalbumin (OVA), and bovine serum albumin (BSA) as model proteins, spanning a range of structural and physicochemical properties. Principal component analysis (PCA) was used to map proteins according to descriptors such as size, charge, hydrophobicity, and stability, enabling the identification of a minimal set capturing maximal diversity (Figure 3A, Section S1 and S8 of SI). In the PCA plot, larger distances correspond to greater differences in protein properties. The four selected proteins for in situ studies were Cyt c, MB, HB, and BSA, which occupied distinct regions in the PCA space, differing in flexibility, size, and charge (Figure 3B).

**Figure 3:**
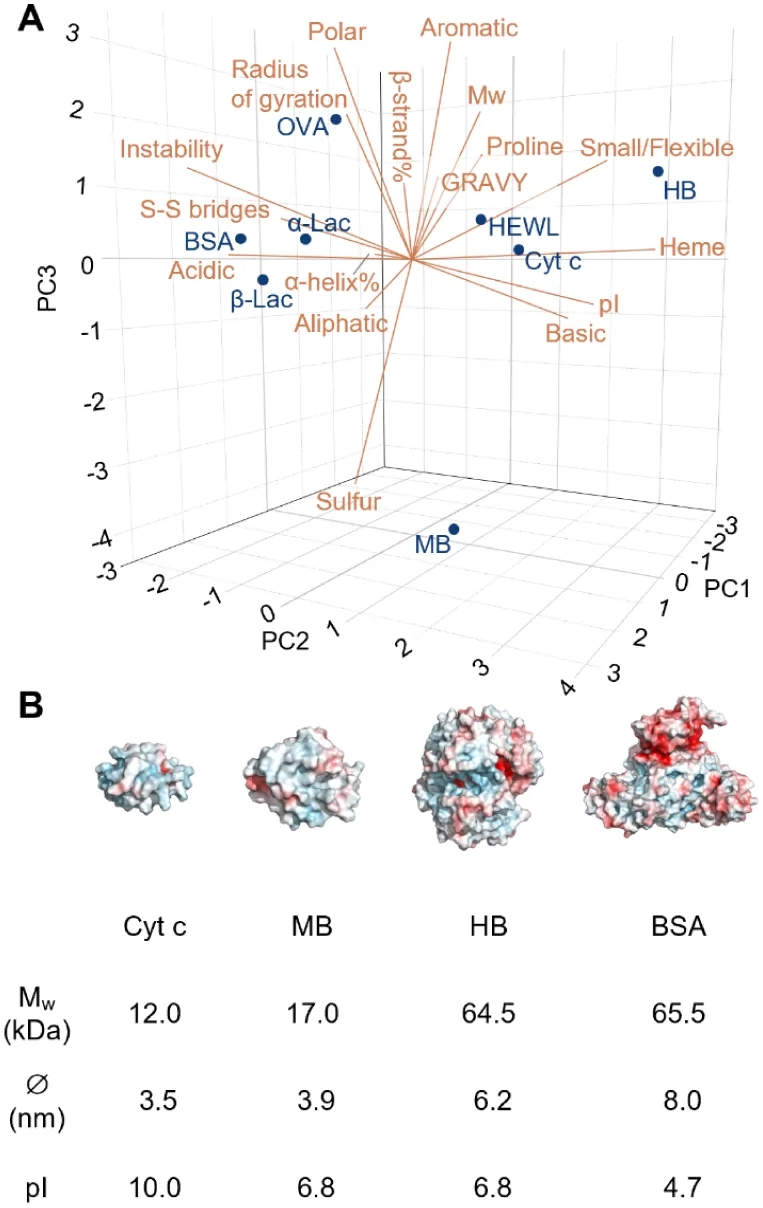
(A) PCA analysis of the selected model proteins for this study with depicted protein structures (see supporting information for interactive plot). (B) Selected model protein structures (cytochrome c (Cyt c), myoglobin (MB), hemoglobin (HB), and bovine serum albumin (BSA)) and their properties (*M*_w_: molecular weight, ⌀: diameter, pI: isoelectric point). The proteins were colored according to their surface charges using PyMOL’s APBS plugin (light blue: positive, red: negative).

As host material, we used the Zr-based MOF NU-1000 due to its large pore apertures (∼3 nm), high chemical stability in aqueous environments, and widespread use in enzyme immobilization studies.^41,47,53^ Powder X-ray diffraction (PXRD) confirmed the successful synthesis of NU-1000, and the full structural and textural characterization, provided in the Supporting Information (Section S2 and S9 of Supporting Information, Table S5-6, Figures S4-10), is consistent with previous reports.^31^ For spectroscopic measurements, NU-1000 particles were deposited onto Si ATR crystals to form a stable MOF film (Figure 2A, Section S3 of SI). The surface coverage of the MOF layer was quantified (Section S4 of SI) to ensure reproducible and comparable loading across experiments, with a typical coverage of ∼15% of the ATR crystal’s surface.

For the *in situ* monitoring of protein adsorption, we used a custom-designed flow cell mounted on the ATR crystal, allowing continuous exchange of solutions (Figure 2A, Section S3 of SI, Figure S1). Prior to measurements, the system was equilibrated with Milli-Q water until a stable baseline was achieved, confirming the stability of the MOF coating under flow conditions. Unless otherwise noted, the bare or MOF-coated ATR crystals in the presence of water served as background spectra. Next, protein solutions of different concentrations (20 μM or 80 μM) were applied, alternating with Milli-Q water washing steps. Because the evanescent field probes only a finite volume extending from the ATR surface and because MOF particles only partially cover the probed volume (Figure 2A), the recorded signal contains contributions from proteins in solution, proteins adsorbed on the native SiO_*x*_ surface of the ATR crystal, and proteins associated with the MOF particles. To disentangle these contributions, washing steps were introduced following protein exposure to selectively remove non-interacting species, thereby isolating signals arising solely from adsorbed and confined proteins (Figure 4A).

**Figure 4:**
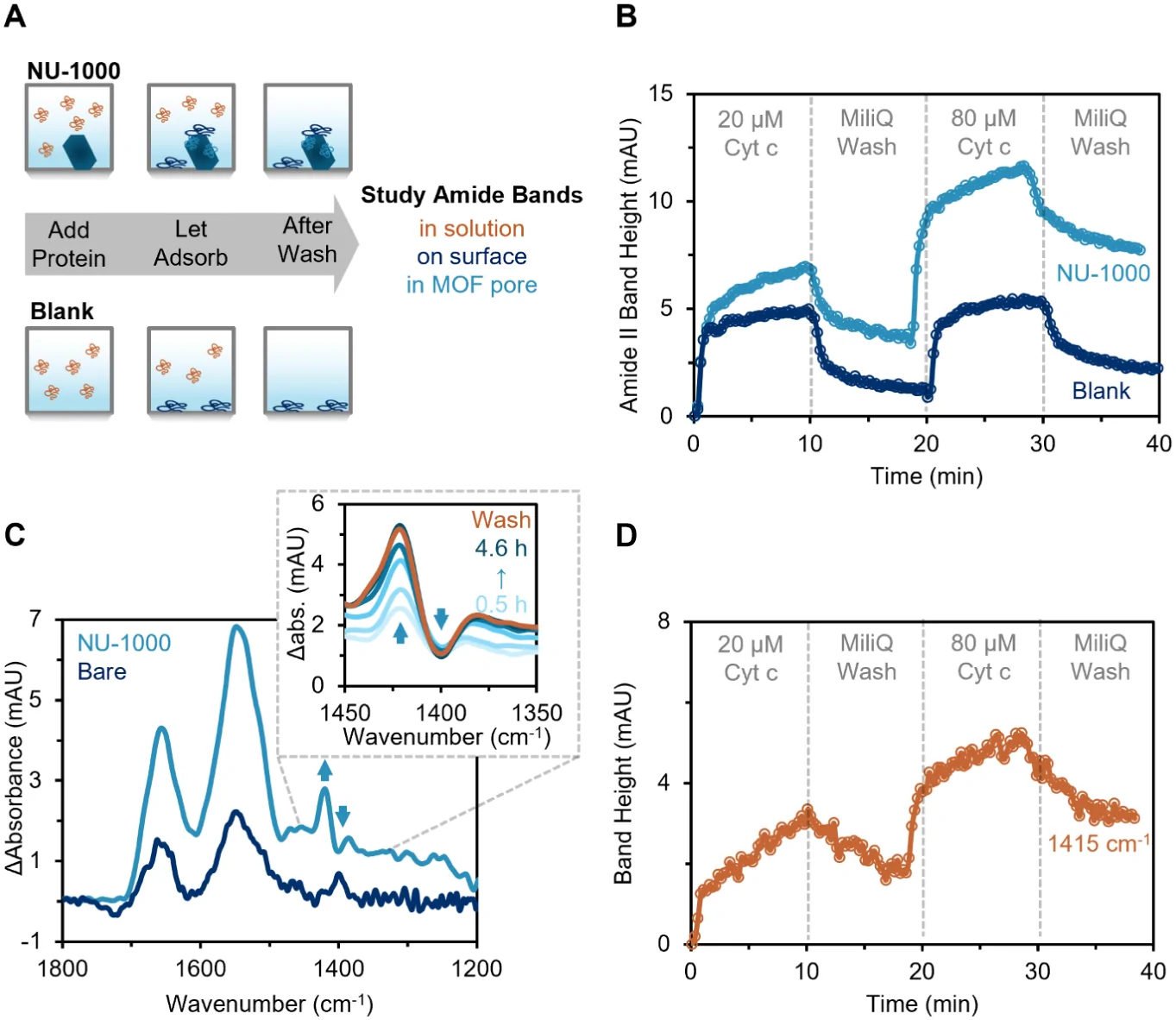
(A) Schematic overview of the protein species in the ATR-FTIR cell during the different steps of the protocol with a MOF covered (top) and a bare ATR crystal (bottom). (B) Maximum amide II band height of Cyt c at indicated concentrations followed over time for the bare and NU-1000-coated ATR crystals. The gray dashed lines indicate solvent swaps between protein solutions and Milli-Q water as depicted in A. (C) Resulting IR spectra of Cyt c (80 μM) on bare and NU-1000-coated ATR crystals. The extra panel zooms in on the spectra from the extended adsorption experiment of Cyt c (20 μM, 4.6 h, blue traces) onto an NU-1000-coated ATR crystal, followed by a 1 h Milli-Q water washing step (orange trace). (D) Band height at 1415 cm^–1^ over time obtained from an NU-1000-coated ATR crystal.

### Adsorption and MOF Pore Entry of Cytochrome c

Cyt c (12 kDa, pI ≈ 10, ⌀ ≈ 3.5 nm) was selected as a model protein whose dimensions are comparable to the pore aperture of NU-1000 (⌀ ≈ 3 nm), making pore entry plausible, as previously demonstrated (Figure 4, Figures S11–S12).^54^ To probe its uptake mechanism, we analyzed the evolution of the amide II band during Cyt c exposure (Figure 4B). On the bare ATR crystal, the amide II intensity increases rapidly and reaches a plateau within 2 min, consistent with fast saturation and surface adsorption of the protein onto a flat surface. In contrast, the NU-1000-coated ATR crystals exhibit a continuous increase in amide I and II intensity over extended time scales, without reaching equilibrium within the experimental time window. Because the intensity of the amide II band scales with the amount of protein within the evanescent field, this sustained increase indicates ongoing accumulation beyond simple surface coverage. While the MOF-coated ATR crystal provides a larger externally accessible surface area compared to the bare ATR crystal due to the deposited particles, protein adsorption onto these exposed surfaces is still expected to equilibrate on similar short time scales (compare with fast adsorption onto microporous UiO-66 below).^55,56^ The continued increase in signal intensity, beyond the initial adsorption phase observed for the MOF-coated ATR crystal, is therefore attributed to a slower process, consistent with the gradual uptake of proteins into MOFs reported previously.^55,56^ To facilitate comparison with previous studies, we performed an extended adsorption experiment lasting >3 h (Figure 4C additional panel), where we found consistently increasing intensity of amide bands. This enabled us to determine an apparent diffusion coefficient of 7.5 × 10^–16^ m^2^ s^–1^ (see Section S5 of SI, Figure S13 and Table S7), which is in good agreement with literature values obtained using CFM.^18,44,54^

To further distinguish between protein adsorbed on the external MOF surface and protein that had penetrated into the pores, we performed a complementary digestion experiment following previously published methodology (see Section S6 of SI, Tables S8–S9).^47,57^ Upon Cyt c adsorption, the MOF was treated with trypsin, a protease that hydrolyzes protein accessible at the external particle surface. The MOF framework was subsequently digested, and the remaining Cyt c was quantified by inductively coupled plasma optical emission spectrometry (ICP-OES) by determining the iron content present in Cyt c-bound heme. Approximately 33% of the adsorbed Cyt c remained after trypsin treatment, indicating that a substantial fraction of the protein was protected from enzymatic digestion and, therefore, inaccessible at the external MOF surface.

Having established the distinct uptake behavior of Cyt c into NU-1000, we next examined the corresponding protein vibrational signatures after 10 min of protein–surface interaction. ATR-FTIR spectra display the characteristic amide I and amide II bands, with significantly higher absorbance for the MOF-coated sample than for the bare ATR crystal, consistent with enhanced protein accumulation. Cyt c in solution exhibits amide I (3.0 mAU) and amide II bands (3.0 mAU), which are consistent with the typical amide I ≥ amide II band trend for folded proteins (Figures 2 and 4C).^58^ However, spectra obtained on both bare and MOF-coated ATR surfaces show a decrease in the amide I band relative to amide II, resulting in an inverted intensity ratio (amide I < amide II; 1.4 mAU < 2.3 mAU for bare ATR and 4.2 mAU < 6.8 mAU for MOF-coated samples). In addition to this change in band height ratio, alterations in band shape were also observed in the normalized spectra (Figure S14), most notably a broadening of the amide I band upon interaction with the MOF.

These spectral features are characteristic of proteins interacting with solid interfaces^59^ and indicate that Cyt c experiences a modified local environment upon association with NU-1000. The inversion of the amide I/II ratio is commonly associated with a constrained or “flattened” protein conformation at interfaces.^60^ At the same time, the broadening of the amide I band and the relative enhancement of the amide II band suggest changes in hydrogen bonding and backbone conformational dynamics.^61^ Together, these observations are consistent with a confinement-induced perturbation of the protein structure. Importantly, this spectral response does not imply a specific secondary-structure transition but rather reflects a redistribution of backbone environments, indicative of partial structural perturbation rather than complete refolding.^62^

### Establishing the IR Absorbance at 1415 cm^–1^ as a Reporter of Protein Uptake

At higher Cyt c concentrations (80 μM), the spectral features containing the carboxylate stretching modes around1400 cm^–1^ and 1620 cm^–1^ in the MOF-coated sample become more pronounced, providing further insight into protein–MOF interactions. These bands originate from vibrational modes of the MOF framework and are absent for the bare ATR crystal. Specifically, the symmetric carboxylate stretching mode of NU-1000 (Figure S2) at around 1400 cm^–1^ shifts to higher wavenumbers (1415 cm^–1^) upon introduction of Cyt c, appearing as a pronounced differential feature (Figure 4C). Since the IR spectra of pure MOF is used as the background which is subtracted from this spectrum, the observed changes reflect protein-induced changes of MOF structure. Positive bands correspond to new absorbance (e.g., protein uptake), while shifted or negative features indicate perturbations of the MOF framework. Thus, the emergence of the differential band at 1415 cm^–1^ indicates that the local environment of the NU-1000 carboxylate groups is perturbed during protein uptake. This shift is consistent with changes in hydrogen bonding and coordination environment at the Zr–carboxylate bond, suggesting direct interaction between the protein and MOF linkers and/or protein penetration into the pore environment.^63^ A similar, but less pronounced, effect is observed for the asymmetric COO^–^ stretch near 1605 cm^–1^, though partial overlap with the protein amide I region makes this spectroscopic feature less clear (Figure S12).

To further probe the origin of the band at 1415 cm^–1^ independently of a large protein guest, analogous *in situ* experiments were performed with the dipeptide glycylglycine (Gly–Gly). Exposure of NU-1000 to Gly–Gly likewise produced a shift in the carboxylate-associated region and the characteristic differential response around 1415 cm^–1^ (Figure S15). This observation supports assignment of this feature to a guest-induced perturbation of the local carboxylate environment rather than to a Cyt c-specific vibrational contribution. Interestingly, the Gly–Gly-induced band appearance at 1415 cm^–1^ developed gradually over time. Given the much smaller dimensions of the dipeptide relative to the NU-1000 pore aperture, this slow evolution is unlikely to originate from steric restrictions to pore entry and likely reflects slow equilibration of the guest within the MOF pore environment and its interactions with the framework. In addition, flowing Milli-Q solutions corrected to different pH values over MOF-coated ATR crystals also produced a subtle and reversible differential feature at 1415 cm^–1^, indicating sensitivity to environmental conditions (Figure S18). This further demonstrates the sensitivity of the carboxylate vibrations to changes in the local framework environment.

For Cyt c, several additional observations connect this framework response specifically to protein uptake. The 1415 cm^–1^ feature is absent when the same protein solution interacts with the bare Si ATR crystals (Figures S11 and S14C), excluding protein adsorption on the silica surface as its origin. Moreover, the band at 1415 cm^–1^ closely follows the gradual increase in amide II intensity (Figure 4B vs. 4D), linking framework perturbation directly to protein uptake. Given the comparable sizes of Cyt c and NU-1000’s pores, we interpreted this differential feature as an indication that the protein is entering the pores of the MOF material.

Analogous adsorption experiments were performed with microporous UiO-66, whose pores are inaccessible to Cyt c, and mesoporous PCN-222, whose pores (Ø ≈ 3.2 nm; see Table S5) can accommodate the protein to further support this interpretation (Figures S13 and S16). Synthetic procedure and characterization of the used UiO-66 and PCN-222 can be found in our previous works and SI.^33,64^ Together with the time-dependent uptake behavior and complementary digestion experiments, these observations establish the 1415 cm^–1^ differential band feature as a useful spectroscopic reporter of protein–MOF interactions associated with pore uptake.

### Influence of Cyt c Conformational State on Pore Access

Beyond pore-size compatibility, our results further indicate that the conformational state of the protein influences access to the MOF pore environment. Following the extended Cyt c adsorption experiment, the band at 1415 cm^–1^ remained pronounced even after 1 h of washing with Milli-Q water (Figure 4C, additional panel). Whereas weakly or reversibly bound protein is expected to be removed during prolonged washing, persistence of the framework perturbation indicates a strongly interacting and/or pore-confined protein population. This is consistent with the strong binding and slow desorption of proteins that is supported by our trypsin digestion experiments and previous reports for Zr-based MOFs.^18,33^

To probe whether partial unfolding facilitates pore entry, we performed *in situ* IR experiments with acid-denatured Cyt c (Figure S17). Because the dimensions of folded Cyt c are close to the pore aperture of NU-1000, partial unfolding may alter the protein shape and effective cross-section, thereby facilitating passage through the pore opening.^18,46,65^ Acid-denatured Cyt c produced a pronounced differential band at 1415 cm^–1^ after only 10 min of incubation, which remained intense even after a washing step (Figures S17). The more rapid appearance and persistence of this framework response compared with folded Cyt c are consistent with enhanced access to the pore environment upon partial unfolding. These results therefore suggest that, for proteins close to the pore-size limit, pore uptake depends not only on geometric compatibility but also on the protein conformational state.

### Generalization of Protein Uptake in NU-1000 Pores

Having established the 1415 cm^–1^ differential band as a spectroscopic signature associated with Cyt c uptake into NU-1000 pores, we next investigated whether this response extends to proteins with different sizes and physicochemical properties, particularly pI. The pI corresponds to the pH at which the net surface charge of a protein is zero. Likewise, NU-1000 exhibits a pI of 4.3, as determined by zeta potential measurements.^47^ Consequently, at the pH values probed in this work (i.e., above the pI of NU-1000), the MOF surface carries a net negative charge. Since electrostatic interactions govern protein adsorption at the MOF interface, proteins carrying an opposite net charge to the MOF are expected to interact more strongly with the surface, potentially promoting adsorption-induced conformational changes or partial unfolding.^18,56^

As a first test of this hypothesis, we selected HB (64.5 kDa, pI ≈ 6.8, ⌀ ≈ 6.2 nm), whose dimensions significantly exceed the pore aperture of NU-1000. At the pH values probed here, HB is expected to carry a neutral-to-positive net charge relative to the negatively charged NU-1000 surface, favoring interfacial adsorption. However, its dimensions significantly exceed the pore aperture of NU-1000, rendering pore entry unlikely (Figure 5, Figures S19-24). While strong amide signals of adsorbed HB are observed for both bare and MOF-coated ATR crystals, no differential MOF band at 1415 cm^–1^ is detected. This absence indicates that HB does not perturb the pore linker environment and therefore does not enter the MOF pores. Instead, HB exhibits similar adsorption behavior on both bare and MOF-coated ATR crystal surfaces, with comparable uptake kinetics and only minor spectral differences (Figures S19-24). Upon normalization, subtle features emerge, including a band near ∼1695 cm^–1^ consistent with refolding and β-sheet formation^66^ and slight broadening of the amide II (Figure 5). These changes suggest structural rearrangement upon surface adsorption, likely associated with formation of protein multilayers, which is consistent with previous reports of HB forming mono-, bi-, or multilayers on solid interfaces.^67–69^ The absence of significant changes in the MOF linker region also supports the conclusion that HB interacts primarily with the external surface.

**Figure 5:**
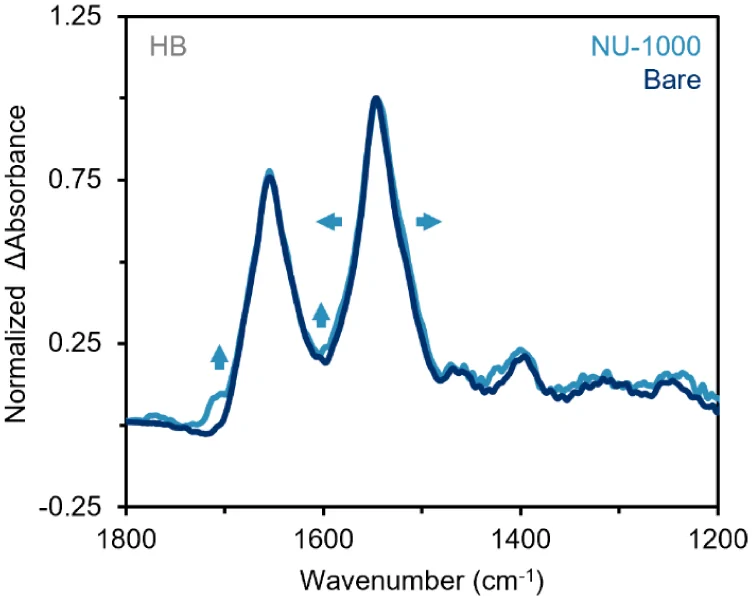
Normalized FTIR spectra of HB (80 μM) after Milli-Q water wash on the bare or NU-1000-coated ATR crystals.

The contrasting behavior of Cyt c and HB highlights the importance of size compatibility for pore access. However, as these two proteins also have significantly different pIs, electrostatic interactions could be contributing to protein–MOF affinity and confinement as well. Cyt c (pI ≈ 10) carries a net positive charge under the experimental conditions, whereas HB (pI ≈ 6.8) is expected to be nearly neutral or slightly negatively charged around pH 7.0. To probe the effect of protein charge, HB adsorption was investigated at both pH 7.0 and pH 5.0 (Figures S19–S22), where HB transitions from near neutral/slightly negative to positively charged. Despite increasing the positive charge of HB, no MOF linker perturbation at 1415 cm^–1^ was observed, indicating that electrostatic attractions alone are insufficient to drive pore entry when steric constraints dominate. Instead, HB adsorption appears to be governed by protein–protein interactions, leading to multilayer formation that likely shields the MOF surface and does not lead to protein entering the pore environment.

Having established that large proteins such as HB remain surface-bound, we next examined adsorption of MB (16.9 kDa, pI ≈ 6.8, ⌀ ≈ 3.9 nm), a borderline case between Cyt c and HB, whose size exceeds the pore aperture only slightly (Figure 6, Figures S25-30). Upon MB adsorption at pH 7.4, the differential MOF band at 1415 cm^–1^ emerges, indicating that MB can access and perturb the MOF linker environment. However, this feature is less intense and develops more slowly compared to Cyt c, suggesting sterically hindered and partial pore entry. In addition, subtle broadening of the amide I and II bands and the appearance of a new feature in the 1620–1610 cm^–1^ region are observed (Figure 6A). While this region can be associated with β-sheet formation,^66^ the absence of a corresponding band near 1690 cm^–1^, suggests that these changes instead originate from perturbations to the asymmetric COO^–^ band of the MOF linker vibrations rather than a clear secondary-structure transition.

**Figure 6:**
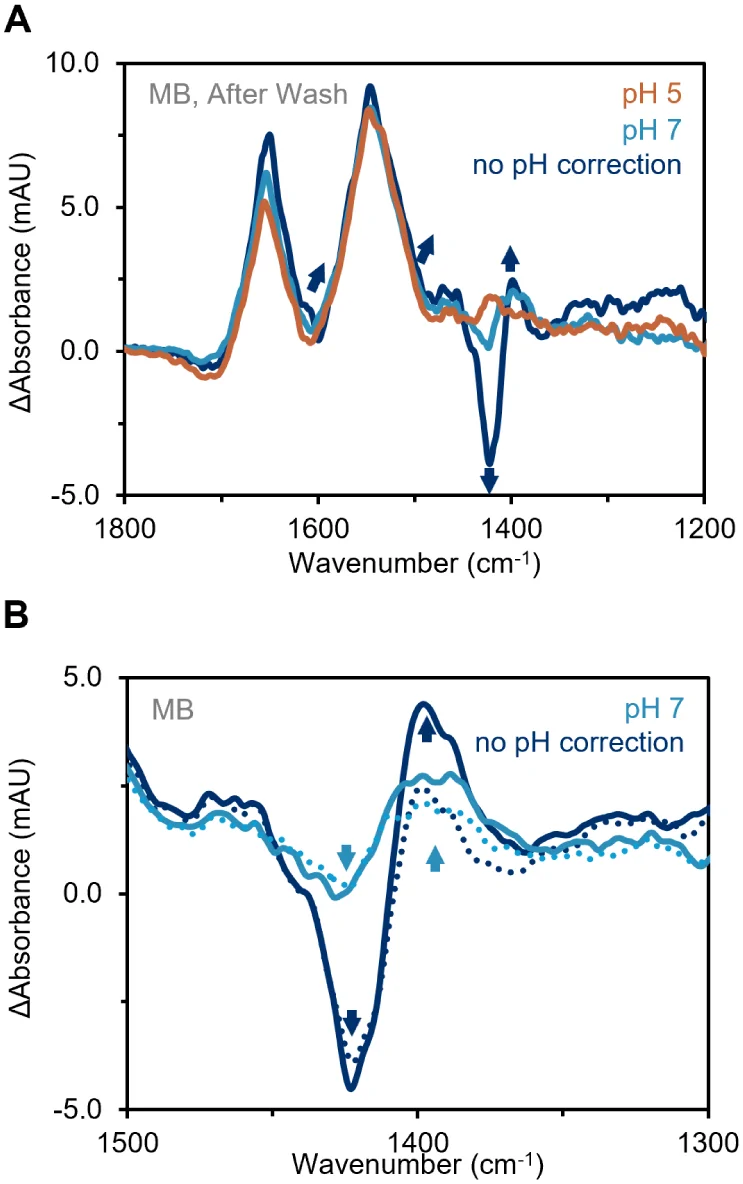
(A) FTIR spectra of MB (80 μM) after Milli-Q wash of NU-1000-coated ATR crystals at indicated pHs. (B) Zoomed view of shifting bands around 1415 cm^–1^ of MB (80 μM) after Milli-Q wash of NU-1000-coated ATR crystals with pH correction to pH 7.0 or without pH correction (∼pH 7.4). Full lines: spectra at start of washing step. Dashed lines: spectra at end of washing step.

To further assess the role of electrostatics near the size-exclusion limit, MB adsorption was also evaluated at pH 7.0 and pH 5.0, with the protein quasi-neutral or positively charged, respectively. At pH 7.0, which is near the pI of MB, the ∼1415 cm^–1^ band persists after washing and the amide II intensity reaches values of ∼8 mAU, indicating stable pore engagement and higher protein uptake (Figure 6A). In contrast, at pH 5.0, the overall uptake seems comparable (similar amide II band height), but the amide I band height is reduced (amide I intensity ∼5 mAU) and the 1415 cm^–1^ band is not affected, consistent with weaker and more reversible protein/MOF surface interactions. Although MB carries a slightly higher positive charge at lower pH, adsorption is maximized near MB’s pI, most likely due to reduced protein–protein repulsion facilitating pore entry.^11,41,70^ Interestingly, experiments performed in pure water (pH ≈ 7.4), without any pH adjustment using NaOH or HCl, show a pronounced and persistent 1415 cm^–1^ band and slightly increased amide II band, indicating that ionic strength also plays a critical role. Even small changes in ion concentration (e.g., Na^+^, Cl^−^) significantly influence both the extent and rate of protein uptake, highlighting the importance of screening effects in protein–MOF interactions.

Finally, we investigated BSA (65 kDa, pI ≈ 4.7, ⌀ ≈ 8 nm), a large protein whose size far exceeds the pore dimensions and whose pI is the lowest among the proteins probed in this study (Figure 7, Figures S31-36). At pH 7.0, where BSA is negatively charged, small broadening of the amide II band as well as a small differential feature at 1415 cm^–1^ was observed (Figure 7A). In contrast, without pH correction, the resulting solution had a pH of ∼5.0 and afforded a distinct differential band at 1415 cm^–1^ which appeared immediately after BSA was introduced to the NU-1000-coated ATR crystal and intensified over time (Figure 7B). At this pH, close to BSA’s pI and in the absence of any additional ions, BSA appears to restructure in the presence of NU-1000, potentially enabling partial insertion into the pores despite its native size exceeding the pore diameter. The spectra display broadened amide bands and new, sharp maxima in the amide II region, which are features absent in the spectra obtained with bare ATR crystals. In model polypeptides, splitting of the amide II band has been assigned to the structural reorganization and the formation of new salt bridges.^71,72^ Control experiments confirmed that these spectral changes cannot be reproduced by simple denaturation or ionic effects alone (Figures S35-36). As opposed to the other adsorption experiments, the BSA uptake here quickly plateaus, strongly suggesting that BSA partially enters and then blocks NU-1000’s pores. Together, these results establish size compatibility as the primary determinant of efficient pore access, while electrostatics, ionic strength, and protein structure flexibility modulate the extent and kinetics of confinement.

**Figure 7:**
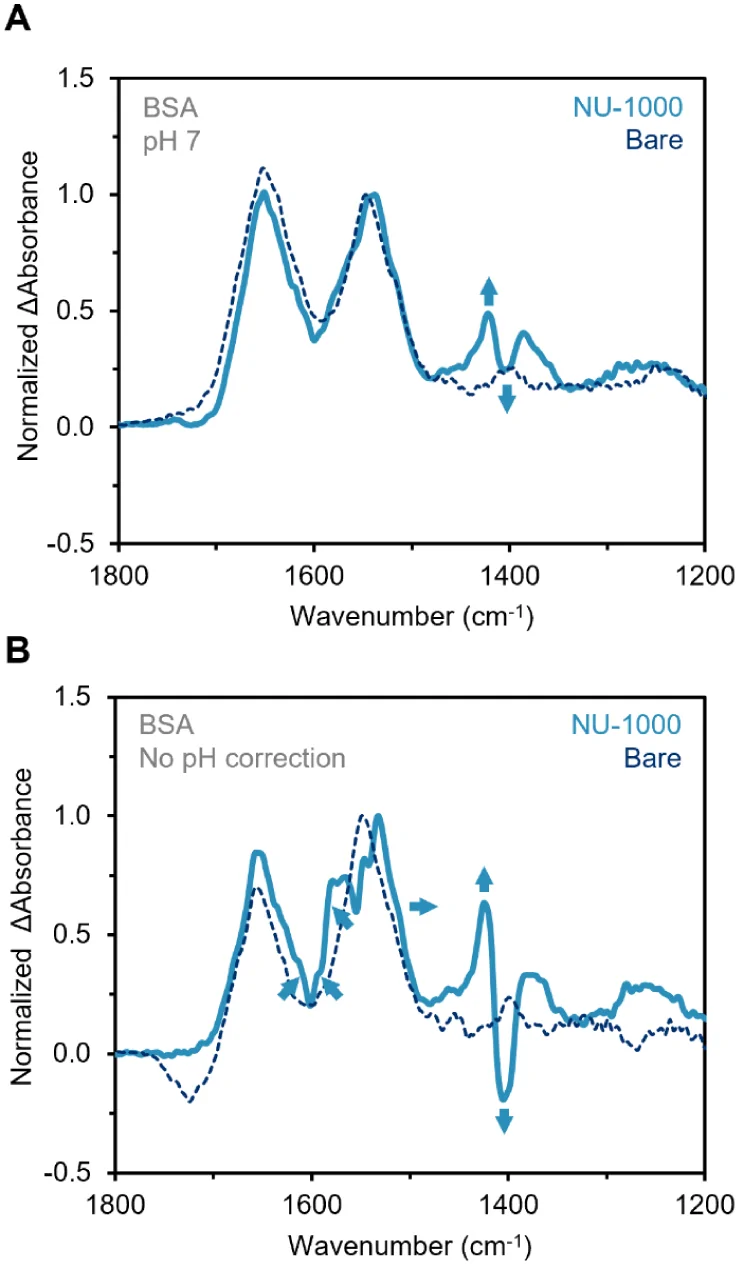
FTIR spectra of BSA (80 μM) after Milli-Q wash for the NU-1000-coated ATR crystals (full lines) and the bare ATR crystals (dashed lines) and with either a correction to pH 7.0 (A) or without pH correction (∼pH 5.0) (B).

### Correlation of Enzymatic Reactivity with Structural Perturbation

To link the structural perturbations observed by ATR-FTIR spectroscopy to functional properties of the protein, we evaluated the enzymatic activity of Cyt c and BSA using model reactions (Figure 8, Section S7 of SI, Tables S10–S12, Figures S37-41). Following the time scales of IR measurements and immobilization protocols reported in the literature, we immobilized the enzymes on NU-1000 for 2 and 24 h. The loading was quantified via depletion of the supernatant (Table S10). After 2 h, Cyt c exhibited a higher loading (0.64 nmol/mg of NU-1000) than BSA (0.19 nmol/mg of NU-1000), consistent with its smaller size and greater accessibility to the MOF pores observed in our *in situ* experiments. Upon prolonged immobilization (24 h), the loading of Cyt c decreased to 0.27 nmol/mg of NU-1000, which we attributed to partial denaturation during extended incubation (see UV–Vis spectra, Figure S37), in agreement with previous reports.^33,73^ In contrast, BSA loading increased to 1.9 nmol/mg of NU-1000 over the same period, consistent with continued adsorption as observed in the uptake experiments with in situ ATR-FTIR spectroscopy.

**Figure 8:**
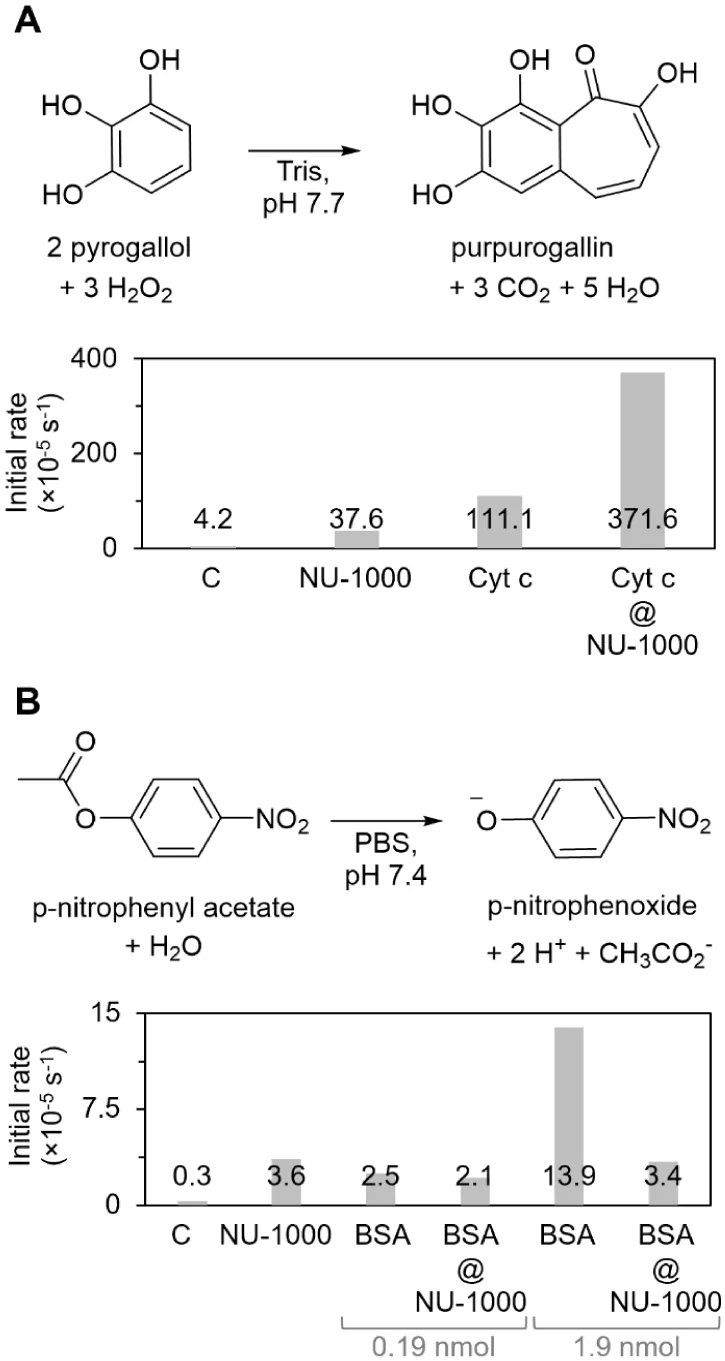
Reaction scheme and initial reaction rates of indicated catalysts for (A) the oxidation of pyrogallol and (B) the hydrolysis of para-nitrophenyl acetate. C represents control reactions in the absence of any catalyst. The gray text indicates the used amount of the enzyme BSA. Reaction rates normalized by the amount of immobilized enzyme resulted in a 100 times faster rates for Cyt c@NU-1000 (1.37×10^–2^ s^–1^ mol^–1^) compared to BSA@NU-1000 (1.11×10^–4^ s^–1^ mol^–1^).

Catalytic activity of immobilized enzymes was assessed using pyrogallol oxidation for Cyt c (Figure 8A) and para-nitrophenyl (NPA) hydrolysis for BSA (Figure 8B). For comparison, equivalent amounts of nonimmobilized protein were tested under the same conditions. For Cyt c (Table S11, Figures S38–S39), immobilization onto NU-1000 leads to a pronounced increase in catalytic activity, with an approximately fourfold enhancement compared to the free enzyme in solution. The control experiments with NU-1000 performed in the absence of Cyt c revealed only minor background activity (Figure 8A). Spectroscopically, Cyt c confined within NU-1000 exhibits a reduced amide I/amide II intensity ratio and broadened amide bands, consistent with a modified hydrogen-bonding environment and increased backbone dynamics.^62^ Importantly, these changes do not indicate complete unfolding but rather a partially perturbed conformational state. Together with the fourfold higher enzymatic activity, these observations suggest that confinement stabilizes a more reactive state.^74,75^ Such behavior is consistent with previous observations of Cyt c at interfaces and in membrane-like environments, where modest structural perturbation leads to an increased peroxidase-like activity.

In contrast, BSA exhibits markedly different behavior upon immobilization (Table S12, Figure S40–S41). The activity of BSA in NU-1000 is significantly reduced compared to the free enzyme. This decrease in reactivity correlates with the spectroscopic signatures indicative of structural constraint. In the IR spectra, the amide II band splits into multiple components, indicating the presence of multiple backbone environments and heterogeneous hydrogen-bonding networks, which have previously been attributed to the formation of new salt bridges.^71,72^ Control experiments performed in the presence of additional salt (Figure S35) suppressed the pronounced response observed in the BSA amide bands. This finding points to strong protein-surface interactions and suggests that redistribution of local electrostatic contacts contributes to salt-bridge formation between BSA and the MOF.

Together with the low BSA uptake, even after 24 h, which is consistent with its larger size and limited pore accessibility, these results suggest that interaction with NU-1000 drives BSA toward a less reactive and more conformationally constrained state. This behavior is likely associated with rigidification of the protein through electrostatic interactions with the MOF and may, upon prolonged incubation, also lead to partial unfolding at the interface. ICP-OES analysis of the trypsin-digested BSA/NU-1000 sample was inconclusive because the sulfur contribution from trypsin could not be distinguished unambiguously from that of BSA.

Collectively, these results highlight that enzymatic activity in MOF environments is not governed solely by size or pI as traditionally assumed.^76^ Instead, protein uptake, structural adaptation, and catalytic performance emerge from the interplay between (i) steric constraints imposed by the pore geometry and accessibility, (ii) electrostatic interactions between protein residues and the charged or polar groups of the framework, and (iii) the local ionic environment in the confined liquid phase. In our systems, favorable molecular dimensions and conformational flexibility facilitate pore confinement and are associated with enhanced catalytic activity, as observed for Cyt c, whereas limited pore accessibility and stronger electrostatic interactions with the framework may constrain protein mobility and promote salt-bridge formation, as observed for BSA. Moreover, even small variations in ionic strength, such as those introduced during pH adjustment, significantly influence adsorption kinetics and pore accessibility. This observation is consistent with reports that increased ionic strength can suppress protein adsorption by competing for binding sites.^77,78^ Yet, such effects are often overlooked in protein@MOF systems, where residual ligands and counterions are rarely considered.^79,80^ ATR-FTIR spectroscopy is sensitive not only to changes in protein secondary structure but also to hydration, electrostatic environment, adsorption geometry, and mass-transport effects. In this study, considering these coupled contributions alongside the independent activity measurements reveals that protein–framework interactions, confinement, and the local solution environment collectively shape the observed catalytic behavior.

## Conclusion

By combining *in situ* ATR-FTIR spectroscopy with enzymatic assays, we correlate protein adsorption and immobilization-induced structural perturbations with changes in enzymatic activity in MOF environments. This approach enables simultaneous tracking of protein uptake and changes in characteristic protein vibrational signatures that are correlated to protein conformation in aqueous solutions under reaction conditions. Importantly, we identify a framework-sensitive spectroscopic response associated with protein uptake into the NU-1000 pore environment. In combination with uptake kinetics, diffusion analysis, pore-size controls, and protease accessibility experiments, this response provides a means to distinguish predominantly surface-bound protein from protein accessing the MOF pore environment. More broadly, our findings show that each protein–MOF combination represents a unique interaction regime, governed by a specific interplay of molecular properties and environmental conditions.

Leveraging this underappreciated approach, our results demonstrate that protein uptake in NU-1000 cannot be described by size exclusion alone. Instead, pore access emerges from a balance of steric constraints, electrostatic interactions, the ionic environment, and the protein conformational state. While large proteins remain predominantly surface-bound, smaller proteins or proteins whose conformation permits passage through the pore aperture can access the pore environment, where interactions with the framework are accompanied by distinct perturbations of the protein vibrational spectrum. These responses are not uniform: confinement can either enhance backbone dynamics and catalytic activity or induce rigidification and a loss of function. Taken together, this work shows that MOFs can actively influence the local environment, structure, and function of immobilized proteins rather than serve solely as passive supports. These insights establish a framework for the rational design of MOF-based systems in which protein behavior can be tuned through controlled confinement, interfacial interactions, and solution conditions.

## Supplementary Material





## Notes

Interactive plot for Figure 3a (ZIP)
General procedures, detailed experimental methods, SEM images, PXRD, ^1^H-NMR spectra, TGA, N_2_ physisorption, ICP-OES results, and FTIR spectra (PDF)
