# Supplementary material for: Folding within Frameworks: Confinement in Zr-MOFs Reshapes Enzyme Structure and Catalytic Activity
Source: J Am Chem Soc. 2026 Sep 16;148(38):41038–48. doi: 10.1021/jacs.6c11124 (PMC13632556; doi:10.1021/jacs.6c11124)
Supplement: Supplementary file 1 [file ja6c11124_si_002.pdf]

Folding within Frameworks: Confinement in Zr-MOFs Reshapes Enzyme Structure and Catalytic Activity

Siene Swinnen[1,2], Maxim Lox[1], Marika Di Berto Mancini,[2] Kilian Declerck[1], Francisco de Azambuja[1], Tatjana N. Parac-Vogt[1]*, Bettina Baumgartner[2]*

[1]Department of Chemistry, KU Leuven, Celestijnenlaan 200F, 3001, Leuven, Belgium

[2]Van't Hoff Institute for Molecular Sciences, University of Amsterdam, Science Park 904, 1098 XH Amsterdam, The Netherlands

Email: tatjana.vogt@kuleuven.be, b.baumgartner@uva.nl.

Keywords: MOFs, protein, adsorption, ATR, IR, zirconium

Contents

Contents ... 1
Materials and Methods ... 2
S1: Principal component analysis (PCA) ... 2
S2: Synthesis and characterization of NU-1000 ... 4
S3: Fourier transform-infrared (FTIR) spectroscopy ... 5
S4: Quantification in ATR-FTIR spectroscopy ... 6
Determination of the path length of the used ATR crystals ... 6
Determination of the MOF's the absorption coefficients ... 7
Determination of the volume fraction of MOF present on the ATR crystal ... 8
S5: Analysis of Cyt c uptake using a one-dimensional diffusion model ... 9
S6: MOF surface treatment with trypsin ... 10
Protein adsorption and MOF digestion ... 10
Trypsin digestion ... 10
MOF digestion and ICP-OES Analysis ... 10
S7: Reactivity study of enzyme@MOF systems ... 11
Immobilization of enzymes ... 11
Hydrolysis of pNPA ... 11
Oxidation of pyrogallol ... 11
Results and Discussion ... 12
S8: Principal component analysis ... 12
S9: MOF characterization ... 14
S10: MOF-protein interaction studies using FTIR-ATR spectroscopy and orthogonal experiments ... 20
S11: Enzymatic reactivity ... 47
References ... 51

Materials and Methods

S1: Principal component analysis (PCA)

The PCA was performed in a similar way as done by Kelly et al. (2024).[1] Protein sequences and structures were obtained from Uniprot and the Protein Databank. Structural descriptors, such as radius of gyration, were calculated from atomic coordinates using the bio3d package (**Table S1**).[2] Heme and Disulfide bridges were added manually. Secondary-structure prediction was performed from primary sequence using the DECIPHER package, and the percentage of helix, β-strand, and coil were calculated for each sequence.[3]

Table S 1: Calculated descriptors based on sequence and crystal structure data from the PDB.

Descriptor name	Description
Molecular Weight	Total molecular mass of the peptide/protein.
Isoelectric Point	pH at which the molecule carries no net electrical charge.
GRAVY Index	Grand Average of Hydropathy; indicates overall hydrophobicity.
Instability Index	Predicts the protein's in vitro stability.
Net Charge at pH 7.4	Net electrical charge at physiological pH.
Aliphatic Index	Relative volume of aliphatic side chains; relates to thermal stability.
Helical Content	Fraction of residues in α-helix conformation.
Strand Content	Fraction of residues in β-strand conformation.
Coil Content	Fraction of residues in random coil conformation.
Radius of Gyration	Measure of 3D compactness from the protein's atomic coordinates (PDB).
Heme	Amount of heme present in the protein
Disulfide Bridges	Amount of disulfide bridges

Sequence-derived descriptors included molecular weight, isoelectric point, GRAVY index, instability index, net charge at pH 7.4, aliphatic index, and secondary structure content and were calculated using the Peptides package.[4] Amino-acid compositions were calculated using protr and were summarized by grouping through physicochemical properties (**Table S2**).[5]

Table S 2: Grouped amino acid properties based on their frequency in their UniProt sequence.

Group	Amino acids	Rationale
Hydrophobic	A, V, L, I, M, F, W, Y	Drive folding into protein core; hydrophobic effect; includes aromatics because they are also hydrophobic.
Aromatic	F, W, Y	π–π stacking, absorbance, spectroscopic properties; overlap with hydrophobic since aromatics are both.
Polar	S, T, N, Q, C	Hydrogen bonding, solubility; Cysteine included here because of –SH polarity.
Acidic	D, E	Negatively charged at neutral pH; electrostatic interactions, pH response.
Basic	K, R, H	Positively charged at neutral pH (though H is partly protonated); DNA/RNA binding, charge interactions.
Sulfur	C, M	Cysteine (disulfide bonds, redox), Methionine (oxidation, start codon). Both contain sulfur.
Small	G, A, S	Promote flexibility and packing; Glycine (flexible), Alanine (helix former), Serine (small polar).
Proline	P	Rigid, helix breaker, special conformational role.

To address the compositional nature of amino-acid fractions, the compositional variables were isolated and centered log-ratio (CLR) transformed using the compositions package.[6] Descriptors were checked for completeness and scaled to mean = 0 and SD = 1. Highly correlated variables (Pearson r > 0.85) were removed to reduce redundancy (**Table S3**).

Table S 3: Overview of removed variables.

Variable 1	Variable 2	Correlation	Removed variable
Hydrophobic	GRAVY Index	0.8836289	Hydrophobic
Net Charge at pH 7.4	Isoelectric Point	0.8511183	Net Charge at pH 7.4

PCA was performed using prcomp() in R to reduce dimensionality and identify major variation axes (**Table S4**).[7]

PC1 (34.8%) primarily separated proteins according to secondary-structure composition and associated physicochemical properties. Positive PC1 loadings were dominated by α-helical content, aliphatic index, radius of gyration, molecular weight, and acidity, whereas β-strand content, random-coil content, and isoelectric point loaded negatively.

PC2 (20.3%) was driven primarily by heme content and small/flexible residues, with additional contributions from instability index, isoelectric point, molecular weight, and amino acid composition.

PC3 (18.6%) contrasted proteins enriched in aromatic, polar, and random-coil residues with proteins enriched in sulfur-containing residues, indicating variation in amino acid composition rather than overall size or secondary structure.

Protein projections onto the PCs were visualized in 2D and 3D, and descriptor contributions were assessed via loadings and squared cosine (cos^2) values.

S2: Synthesis and characterization of NU-1000

NU-1000 was synthesized using an adapted solvothermal synthesis protocol according to a previous publication.[8] A 500 mL VWR pressure plus+ bottle is charged with $ZrOCl_2.8H_2O$ (1.94 g, 6.02 mmol), benzoic acid (54 g, 442 mmol) and 120 mL of N,N-dimethylformamide (DMF). This mixture is sonicated until it became homogeneous, followed by heating at 100 °C for 1 h in an oven. In a second 100 mL VWR pressure plus+ bottle, H4TBAPy (0.800 g, 1.17 mmol) and DMF (40 mL) are added and sonicated until a suspension is obtained. Next, this suspension is heated at 100 °C for 1 h in an oven, affording a clear solution. After both solutions are ready, H_4TBAPy solution is added into the 500 mL VWR pressure plus+ bottle and manually homogenized for a few minutes while the solutions are warm. The reaction mixture is placed in an oven at 120 °C for 8 h. After cooling down to room temperature, the precipitate is recovered through centrifugation and washed with DMF (3 x 45 mL, for 2 h each time). After centrifugation, the precipitate is suspended in 260 mL of DMF and 10 mL of HCl (8 M) is added dropwise while gently manually shaking. This mixture is placed in a 100 °C oven overnight. After cooling down to room temperature, the precipitate is washed with DMF (3 x 40 mL for 2 h each time) and with MeOH (4 x 40 mL for 4 days each time). After allowing the MOF powder to air dry overnight, the MOF was activated in a vacuum oven at 120 °C for 20 h. After synthesis and activation, the MOF was characterized without further treatment.

Powder X-Ray Diffraction (PXRD) patterns were recorded with roughly 7 mg of sample on a Malvern PANalytical Empyrean diffractometer over a 1.3 – 45° 2θ range, using a PIXcel3D solid state detector and Cu anode (Cu Kα1: 1.5406 Å; Cu Kα2: 1.5444 Å).

N_2 physisorption isotherms of 50 mg of sample were measured on a Micromeritics 3flex and TriStar surface analyzers at -196 °C. Prior to measurements, samples were degassed at 120 °C under vacuum for 12 h. Surface areas were calculated using the multi-point BET method applied to the isotherm adsorption branch taking into account the Rouquerol consistency criteria. The micropore volume was calculated at $p/p_0 = 0.5$ using the DFT protocol.[9]

Thermal gravimetric analysis (TGA) of 10 mg of MOF was analyzed with a NETZSCH STA 449 F3 thermal analysis system. Temperature was increased at a rate of 6 °C per minute from 35 to 600 °C.

Scanning electron microscopy (SEM) micrographs were recorded using a JEOL-6010LV SEM. SEM samples were prepared by depositing powder samples onto carbon tape. Before analysis, the samples were coated with a palladium/gold layer using a JEOL JFC-1300 autofine coater under Ar plasma. The average particle sizes were estimated from the obtained images using ImageJ.

Proton (^{1}H) nuclear magnetic resonance (NMR) of the synthesized MOFs after digestion with base. 20 mg of MOF was digested with 1.500 mL of NaOH (1 M; in D_2O) for 24 h at room temperature without stirring. 50 µL of a TMSP-d_4 solution (0.02 M, in D_2O) and 500 µL supernatants of the digestion were combined in an NMR tube. ^{1}H NMR spectra were recorded on a Bruker Avance 400 (400 MHz) spectrometer with 16 scans and a D1 time of 1 second. NMR data was analyzed with TopSpin 4.4.0.

S3: Fourier transform-infrared (FTIR) spectroscopy

Protein adsorption onto MOFs was studied *in situ* using Fourier transform-infrared (FTIR) spectroscopy (Bruker InvenioR FTIR spectrometer with N_2-cooled MCT detector) in attenuated total reflection (ATR) mode. Si ATR crystals (20 x 10 x 0.5 mm, 45°) were cut from double side polished Si wafer (Siegert). MOF films were obtained by drop casting MOF suspensions (2 mg MOF powder in 500 µL methanol) onto the ATR crystal and subsequent heating to 80 °C for 30 min. For each adsorption experiment a freshly prepared MOF-coated ATR crystal was used. MOF coated or uncoated ATR crystals were placed in a home-built polymethylmethacrylaat (PMMA) flow cell with a nitrile O-ring (diameter 14mm, thickness 1.25 mm) connected with 1/16’’ perfluoroalkoxy (PFA) tubing, Super Flangeless™ Ferrule (6-32 Flat-Bottom) and Nut Short Headless (10-32 Flat-Bottom) purchased from IDEX and placed in the FTIR spectrometer (**Figure S1**). The interferometer and sample compartment of the FTIR spectrometer was flushed with N_2. Prior analyte application, the flow cell was flushed with MilliQ water until a stable baseline was achieved. This was typically 45 min.

Protein solutions were applied with 1 mL/min flow using a peristaltic pump (Ismatec Masterflex, Germany). Protein solutions were prepared in MilliQ. pH corrections were performed by the addition of either NaOH or HCl (0.5M). Unless otherwise noted, for each set of experiments first a protein solution of 20 µM was flushed for 10 min in a closed loop through the flow cell followed by a 10 min wash step with MilliQ; after which 80 µM protein solution was applied for 10 min in closed loop through the flow cell, again followed by a wash step for 10 min with MilliQ.

FTIR spectra were derived from single channel spectra. MATLAB (R2024b, The Mathworks Inc., USA) was used to baseline correct and average the obtained spectra. Further fitting of spectra was done in Origin (Version 2022, OriginLab Corporation, USA). Spectra were manually corrected for water vapor by subtraction of water vapor spectra obtained in similar conditions. Savitzky-Golay smoothing was applied to the data using an 11-point window and a quadratic polynomial of degree 2. Normalization of the data was done by the maximum of the Amide II band. Background: blank or NU-1000 coated ATR crystal flushed with water.

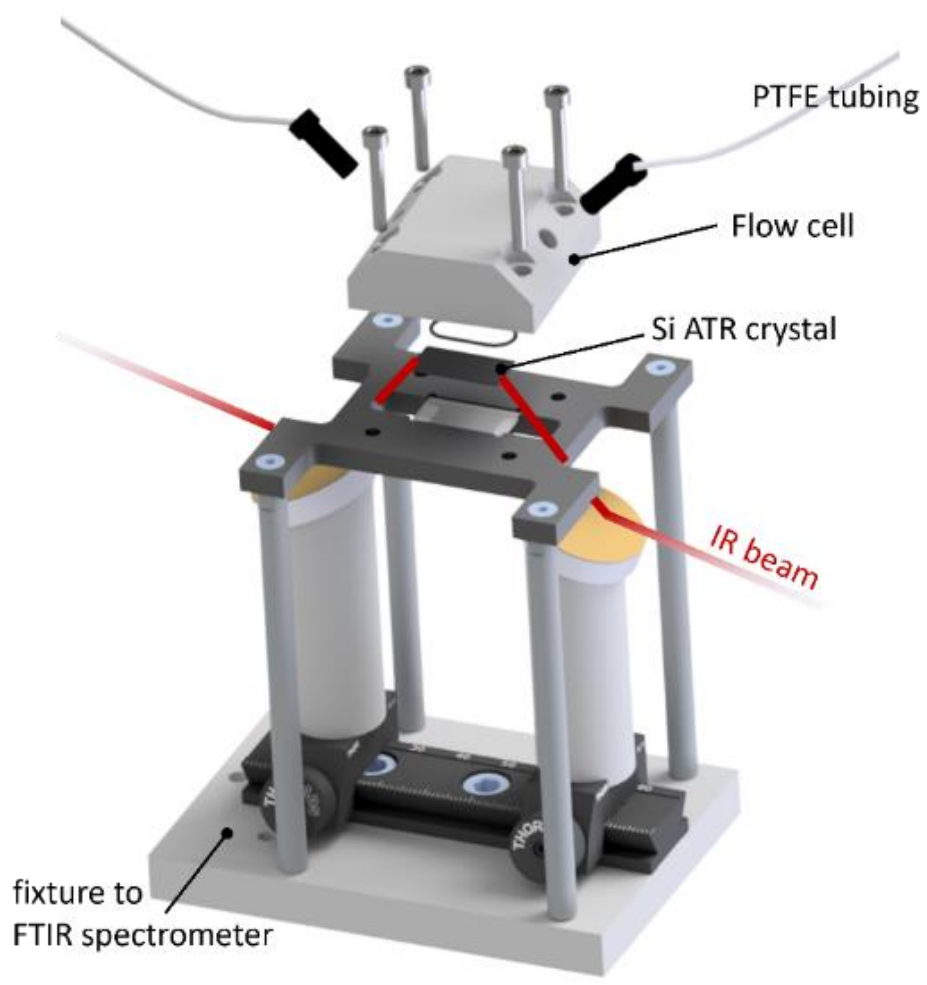

Figure S 1: 3D rendering of the *in situ* Fourier transform-infrared spectroscopy experimental setup.

S4: Quantification in ATR-FTIR spectroscopy

Lambert-Beer's law (**Eq. S1**) was used for quantification via FTIR spectroscopy, with the absorbance (A), the absorption coefficient (ε), the concentration (c), and the path length (d).

$$A = \varepsilon \cdot c \cdot d \qquad \text{Eq. S 1}$$

The amount of analyte (i.e. MOF or protein) present in the evanescent wave on the ATR crystal can thus be determined via **Eq S2** if all parameters are known. The absorbance is known from our measurements. Below is discussed how the path length and absorption coefficients were determined.

$$c = \frac{A}{\varepsilon \cdot d} \qquad \text{Eq. S 2}$$

Determination of the path length of the used ATR crystals

The following section discusses the determination of the effective path length (d_e) for ATR FTIR spectroscopy according to Harrick *et al.* via **Eq. S3** using the evanescent field (E), the ratio of the refractive indices (n_{21}) of the water (n_1) and the ATR crystal (n_2), the angle of incidence (θ), the depth of penetration (d_p), the wavelength (λ_0), and number of active bounces (N).[10]

$$d_e = \frac{n_{21}}{cos\theta} \int_0^{\infty} E^2 dz \qquad \text{Eq. S 3}$$

The evanescent field amplitudes can be integrated over the pathlength of the absorbing rarer medium, which respectively results in the effective pathlengths for the waves parallel to the plane of incidence ($d_{e,\parallel}$) and the waves perpendicular to the plane of incidence ($d_{e,\perp}$) in **Eq. S4**. and **Eq. S5**.

$$d_{e,\parallel} = \frac{\lambda_0}{n_1} \cdot \frac{n_{21} \cos(\theta)\,[2\, sin^2(\theta) - n_{21}^2]}{\pi\,(1 - n_{21}^2)[(1 + n_{21}^2) sin^2(\theta) - n_{21}^2][sin^2(\theta) - n_{21}^2\,]^{1/2}} \qquad \text{Eq. S 4}$$

$$d_{e,\perp} = \frac{\lambda_0}{n_1} \cdot \frac{n_{21}\, cos(\theta)}{\pi\,(1 - n_{21}^2)[sin^2(\theta) - n_{21}^2]^{1/2}} \qquad \text{Eq. S 5}$$

The total effective pathlength for unpolarized light can be rewritten as **Eq. S6**.

$$d_e = \frac{d_{e,\perp} + d_{e,\parallel}}{2} \cdot N \qquad \text{Eq. S 6}$$

The effective path length (d_e) of the used ATR crystal can be determined for a specific wavenumber via the number of active bounces (N) from a reference measurement of a blank ATR crystal, using the known refractive indices of the ATR crystal ($n_{ATR} = 3.42$) and water ($n_{Water} = 1.33$), and the $d_{e,\perp}$ and $d_{e,\parallel}$ calculated.

Determination of the MOF's the absorption coefficients

The absorption coefficient of each MOF was determined via KBr reference measurements via **Eq. S7**.

$$\varepsilon = \frac{A}{\mathrm{c} \cdot \mathrm{d}}$$

Eq. S 7

KBr pellets (blanks and containing known quantities of MOF) of 13 mm diameter wide were prepared using a pellet press. Reference FTIR spectra were recorded in transmission mode.

The pathlength (d) was defined as the pellet thickness and was calculated using the known pellet weight (m_{KBr}), the pellet radius (r), and the density of KBr $(\rho_{KBr} = 2.75\ g\ cm^{-3})$ via **Eq. S8**.

$$V_{KBr} = \pi \cdot r^2 \cdot d = \frac{m_{KBr}}{\rho_{KBr}} \Leftrightarrow d = \frac{m_{KBr} \cdot \pi \cdot r^2}{\rho_{KBr}}$$ Eq. S 8

The band height of the symmetric C=O stretching of the MOF linker (**Figure S2**), using the blank KBr pellet spectrum as background, from the reference measurements was used as the absorbance to determine the absorption coefficient of the MOF.

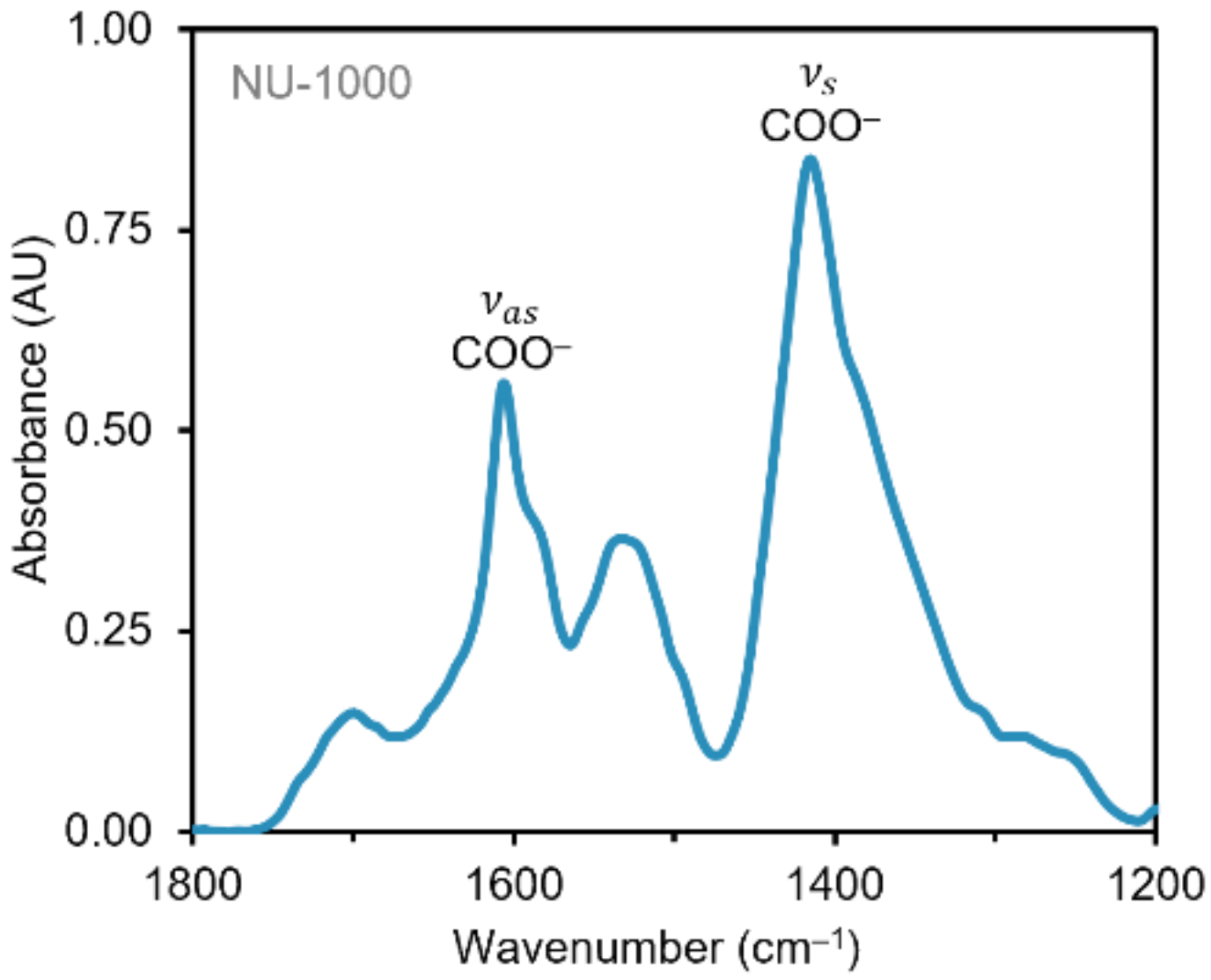

Figure S 2: Overview of the typical carboxylate linker bands of NU-1000.

Determination of the volume fraction of MOF present on the ATR crystal

The amount of MOF present on the ATR crystal can be determined using **Eq. S 2**, using the calculated absorption coefficient of NU-1000, the measured band heights of the symmetric C=O stretching of the MOF linker and the calculated $d_\mathrm{e} = 9.6\ \mu m$ at the same wavenumber obtained with $n_\mathrm{Water} = 1.33$ as approximation.

The volume fraction of MOF (ϕ_{MOF}) present in the evanescent field on the ATR crystal can be determined from the ATR absorbance of the same C=O vibration (A_{ATR} = 0.15) during a measurement of blank ATR crystal, the calculated absorption coefficient of NU-1000, and the calculated effective path length at the corresponding wavenumber.

The volume of the evanescent wave covered by MOF particles can be defined as

$$\phi_{MOF} = \frac{V_{MOF}}{V_E} \qquad \text{Eq. S 9}$$

where (V_{MOF}) the volume occupied by MOF particles and (V_E) is the total evanescent field volume. V_E corresponds to the region of the flow cell probed by the infrared radiation and is given by:

$$\mathrm{V}_E = \pi \cdot {r_{O-ring}}^2 \cdot d_\mathrm{e} \qquad \text{Eq. S 10}$$

with the known radius of the O-ring of the flow cell ($r_{O-ring} = 7\ mm$) and the height of the volume equal to the total effective path length (d_e).

S5: Analysis of Cyt c uptake using a one-dimensional diffusion model

The time-dependent Amide II band height was analyzed using the spatially averaged analytical solution of Fick's second law for one-dimensional uptake into a finite slab. This treatment follows the established Fickian description of molecular transport in nanoporous crystals, including MOFs.[11-14] The rod-shaped crystals were approximated as finite slabs along their longitudinal axes, with uptake assumed to occur symmetrically through both end faces. Surface resistance was assumed to be negligible, corresponding to a constant guest concentration at the crystal ends following exchange of the flow-cell volume.

The initial signal, A0, was fixed to the measured Amide II band height at the selected starting time t0. This time point was chosen after complete exchange of the flow-cell volume, such that subsequent changes in the solution-phase contribution to the Amide II signal were assumed to be negligible. Only data acquired at or after t0 were included in the fit. The elapsed fitting time was therefore defined as:

$$\tau = t - t_0 \qquad \text{Eq. S 11}$$

The total crystal length, ℓ, was determined from SEM images. Because diffusion was assumed to occur from both ends of the crystal, the characteristic diffusion half-length was defined as L = (ℓ/2). The measured Amide II band height was described by:

$$A(t) = A_0 + (A_\infty - A_0)\, F(\tau) \qquad \text{Eq. S 12}$$

where A_∞ is the equilibrium band height and F(τ) is the fractional approach to equilibrium. The fractional uptake was calculated as:

$$F(t) = 1 - \frac{8}{\pi^2}\sum_{n=0}^{N-1}\frac{1}{(2n+1)^2}\exp\left(-\frac{(2n+1)^2\pi^2 D_{app}\,\tau}{4L^2}\right) \qquad \text{Eq. S 13}$$

where D_{app} is the apparent diffusion coefficient and N is the number of terms retained in the truncated diffusion series. In the present analysis, N=10 was used. At τ=0, F(τ) was explicitly set to zero to ensure that the calculated signal satisfied A(0)=A0.

The model assumes that the Amide II band height is proportional to the average amount of Cyt c associated with the MOF crystals over the probed volume. It further assumes a constant apparent diffusion coefficient, a constant surface concentration following introduction of the Cyt c solution, and predominantly one-dimensional transport along the crystal long axis.

The fitted parameters were A_∞ and D_{app}. Their values were determined by minimising the sum of squared residuals between the experimental and calculated band heights. The minimization was performed in MATLAB using the derivative-free Nelder–Mead algorithm implemented in fminsearch. To constrain both the signal amplitude and the diffusion coefficient to positive values, the fitted parameters were expressed in logarithmic form:

$$A_\infty = A_0 + \exp(p_1) \text{ and } D_{app} = \exp(p_2) \qquad \text{Eq. S 14}$$

The characteristic diffusion half-time, defined as the time required for the modelled signal to reach 50% of its total change, was calculated as:

$$t_{50} = \frac{0.1967\, L^2}{D_{app}} \qquad \text{Eq. S 15}$$

S6: MOF surface treatment with trypsin

Protein adsorption and MOF digestion

The MOF surface treatment was adapted from the literature.[11, 15] NU-1000 (5.0 mg) was suspended in 1 mL of either 0.1 mM Cyt c, BSA, or Milli-Q water (control). Suspensions were incubated at room temperature on a shaker for 2 or 4 h. Each condition was prepared in duplicate, with one aliquot designated for direct analysis and the other for subsequent proteolytic digestion. Following incubation, the suspensions were centrifuged at 12 000 rpm for 5 min. The supernatants were collected, and the pellets were washed with 1 mL Milli-Q water by brief vortexing followed by centrifugation at 12 000 rpm for 5 min. The washed pellets were retained for subsequent analysis using the Pierce™ Bradford Protein Assay Kit (Thermo Fisher Scientific). The collected supernatants were diluted fivefold with Milli-Q water prior to protein quantification using the Bradford assay. Briefly, 30 µL of each diluted sample or protein standard was mixed with 270 µL of Bradford reagent in a clear 96-well plate. Cyt c and BSA calibration standards (0, 1, 2, 5, 10, 15, and 20 µM) were prepared analogously. Plates were shaken for 10 min, and absorbance spectra were recorded from 400 to 650 nm. All measurements were performed in triplicate.

Trypsin digestion

The washed NU-1000 pellets were resuspended in 1 mL of 1 mM Tris-HCl buffer (pH 7.8) containing trypsin (Sigma-Aldrich, T4799) at 50 eq. relative to the estimated adsorbed protein concentration (approximately 1.5 mM trypsin). Samples were incubated at 37 °C for 4 h. Following digestion, samples were centrifuged at 12 000 rpm for 5 min, and the supernatants were collected. The resulting pellets were washed twice with 1 mL Milli-Q water, with vortexing and centrifugation at 12 000 rpm for 5 min after each wash. The second wash supernatants were retained for ICP-OES analysis.

MOF digestion and ICP-OES Analysis

For complete digestion of the MOF, pellets were resuspended in 1 mL of 1 M NH_4HCO_3 and incubated at room temperature for 24 h without stirring. For ICP-OES analysis, Zr, S, and Fe calibration standards (0.1–50 ppm) were prepared in 2% HNO_3. Samples were diluted fivefold by mixing 1 mL of sample with 4 mL of 4% HNO_3, affording a final HNO_3 concentration of approximately 2 %. The resulting acid concentration was selected to compensate for neutralization by NH_4HCO_3 during sample preparation. ICP-OES measurements were performed in both axial and radial viewing modes using two analytical wavelengths for Zr and three wavelengths each for S and Fe. Protein, trypsin, and buffer controls were analyzed alongside the NU-1000 samples.

S7: Reactivity study of enzyme@MOF systems

Immobilization of enzymes

The immobilization procedure was adapted from the literature.[16] 5 mg of NU-1000 powder was added to 1mL of a Milli-Q solution of BSA or Cyt c (20 μM) and stirred for either 2 or 24 h at room temperature. The resulting enzyme@MOF composite was isolated by centrifugation at 12000 rpm for 5 min, and the supernatant was collected and kept for analysis. The enzyme@MOF solid was used for the catalytic reaction. The amount of enzyme immobilized in NU-1000 powder was determined by subtracting the amount of enzyme measured in the supernatant from the initial total amount. For BSA, the concentration in the supernatants was quantified the Pierce™ Bradford Protein Assay Kit (Thermo Fisher Scientific). For Cyt c, the concentration was determined by UV-Vis absorbance at 409 nm, using a 5-point calibration curve. Each immobilization experiment was performed *in triplo*.

Hydrolysis of pNPA

The kinetics of pNPA hydrolysis catalyzed by free BSA, NU-1000, BSA@NU-1000, or solvent alone were monitored by following the absorbance of nitrophenolate ion at 400 nm using a UV-Vis spectrophotometer (Ocean Insight with light source) in 1 cm path cuvette.[17] A pNPA stock solution (5 mM) was prepared in pure acetonitrile due to its low solubility in water and to prevent spontaneous hydrolysis. A fresh BSA stock solution was prepared in PBS. The final concentration of pNPA and BSA in the cuvette were adjusted depending on the experiment (65 μM pNPA, and 63 or 619 μg/ml BSA, respectively). pNPA hydrolysis was also performed in the absence of any catalyst as control to confirm the hydrolysis rate from the solvent was negligible. For the reaction catalyzed by free BSA or by PBS alone, the pNPA solution and the BSA solution (or PBS) were mixed directly in a plastic cuvette to have a final volume of 2 mL (1 mL of ACN and 1 mL of PBS), and the absorbance at 400 nm was recorded over time. For the reaction catalyzed by solid materials (NU-1000 or BSA@NU-1000), 5 mg of the solid were mixed in a vial with the appropriate amount of substrate in a total volume of 2 mL (1 mL ACN and 1 mL water) under continuous stirring. Aliquots were taken every 5 or 10 min, diluted in PBS, and analyzed by UV-Vis spectroscopy at 400 nm. Because the MOF exhibits residual absorbance, the baseline was taken from the first spectrum (time zero). For all experiments, absorbance at 400 nm was plotted as a function of time, and the initial rate was obtained from a linear fit.

Oxidation of pyrogallol

The kinetics of pyrogallol oxidation catalyzed by free Cyt c, NU-1000, Cyt c@NU-1000, or solvent alone were monitored by following the absorbance of purpurogallin at 420 nm using a UV-Vis spectrophotometer (Ocean Insight with light source) in 1 cm path cuvette.[18] Stock solutions of Pyrogallol (30 mM), Tris buffer (1 M, pH 7.7), freshly prepared Cyt c (0.2 mg/mL), and freshly prepared H_2O_2 (30 mM) were prepared in Milli-Q. Pyrogallol oxidation was also performed in the absence of any catalyst to confirm that the reaction rate in Tris buffer solution under air was negligible. For reactions catalyzed by free Cyt c or by buffer alone, the substrate, oxidant, Tris buffer, and Cyt c (when applicable) were mixed directly in a plastic cuvette to have a final concentration of 5 mM, 26.4 mM, 0.1 M, and 40 mg/mL, respectively, in a total volume of 1 mL. The absorbance at 420 nm was recorded over time, while continuously stirring the solution. For the reaction catalyzed by solid materials (MOF or Cyt c@NU-1000), 1 mg of the solid were added to the cuvette together with the same reagent concentrations described above and a final volume of 2 mL to reduce the scattering derived from the solid. The mixture was continuously stirred while monitoring the absorbance at 420 nm. Because the MOF exhibits residual absorbance, the baseline was taken from the first spectrum (time zero). For all experiments, absorbance at 420 nm was plotted as a function of time, and the initial rate was obtained from a linear fit.

Results and Discussion

S8: Principal component analysis

Table S 4: Comparison of different protein cleaving agents and their properties.

Protein	Cyt c	MB	HEWL	α-Lac	BSA	HB	Ova	β-Lac
**Uniprot code[a]**	P00004	P68082	P00698	P00711	P02769	P01966 (α)<br>P02070 (β)	P01012	P02754
**PDB code[b]**	1HRC	1AZI	1IEE	7eka	1ao6	6IHX	1OVA	1BEB
**Disulfide Bridges**	0	0	4	4	17	0	1	2
**Heme**	1	1	0	0	0	4	0	0
**Molecular Weight**	11701	16951	14313	14186	66432	61933	42750	18281
**Isoelectric Point**	10.201	8.077	9.212	4.586	5.668	8.234	4.957	4.559
**GRAVY Index**	-0.902	-0.396	-0.472	-0.453	-0.475	0.017	-0.006	-0.162
**Instability Index**	15.561	10.114	16.091	27.384	40.112	6.587	37.179	42.430
**Net Charge at pH 7.4**	8.809	0.408	6.886	-8.039	-21.374	2.560	-12.602	-8.647
**Aliphatic Index**	59.135	89.346	65.116	83.171	76.141	92.787	90.182	102.407
**Alpha Helix Content**	30.769	60.131	28.682	35.772	62.264	49.303	58.181	54.938
**Beta Strand Content**	16.346	11.765	14.729	18.699	8.576	10.105	10.909	11.728
**Random Coil Content**	52.885	28.105	56.589	45.528	29.160	40.592	30.909	33.333
**Radius of Gyration**	19.180	14.740	14.479	14.289	38.463	23.053	34.655	22.178
**Hydrophobic**	0.308	0.399	0.357	0.350	0.362	0.453	0.447	0.432
**Aromatic**	0.087	0.072	0.093	0.098	0.084	0.084	0.086	0.061
**Polar**	0.192	0.131	0.326	0.293	0.223	0.171	0.236	0.210
**Acidic**	0.115	0.137	0.070	0.163	0.170	0.094	0.122	0.160
**Basic**	0.231	0.209	0.140	0.130	0.170	0.164	0.109	0.123
**Sulfur**	0.019	0	0.062	0.065	0.060	0.010	0.016	0.031
**SmallFlex**	0.173	0.229	0.264	0.130	0.156	0.251	0.239	0.160

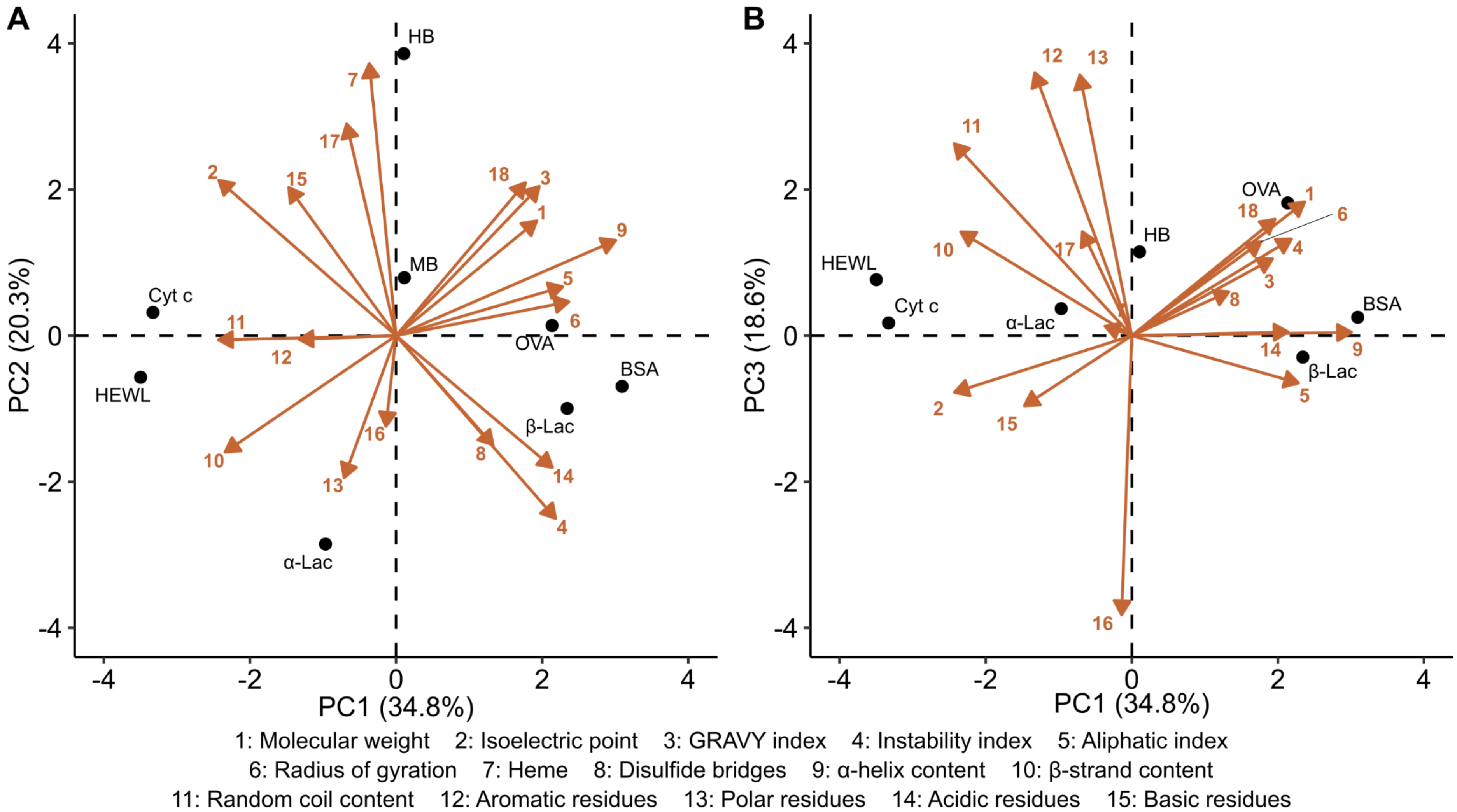

Figure S 3: 2D views of the 3D PCA analysis.

The PCA successfully reduced multidimensional protein descriptors into biologically interpretable axes to visualize major and subtle differences in the probed protein's physicochemical properties.

S9: MOF characterization

Table S 5: Overview of the structural properties of MOFs in this work.

MOF	Particle size (μm)	BET surface area (m²/g)	Micropore area (m²/g)	External surface area (m²/g)	Pore size (Å)	Missing linkers
**NU-1000**	3.835 ± 0.230	2.219	318.2	1.901	12; 15; 31	0.6[a]
**UiO-66**[b]	0.229 ± 0.039	1155	973	182	7.7; 11; 16; 19	1.0
**PCN-222**[c]	20	1.960	223	1734	32	

[a]More information on how the missing linker content is estimated, is explained below Fig. S6.
[b]The material from a previous publication was reused for these experiments and was fully characterized. More information can be found in this reference.[19]
[c]The missing linker content was not determined for this MOF.

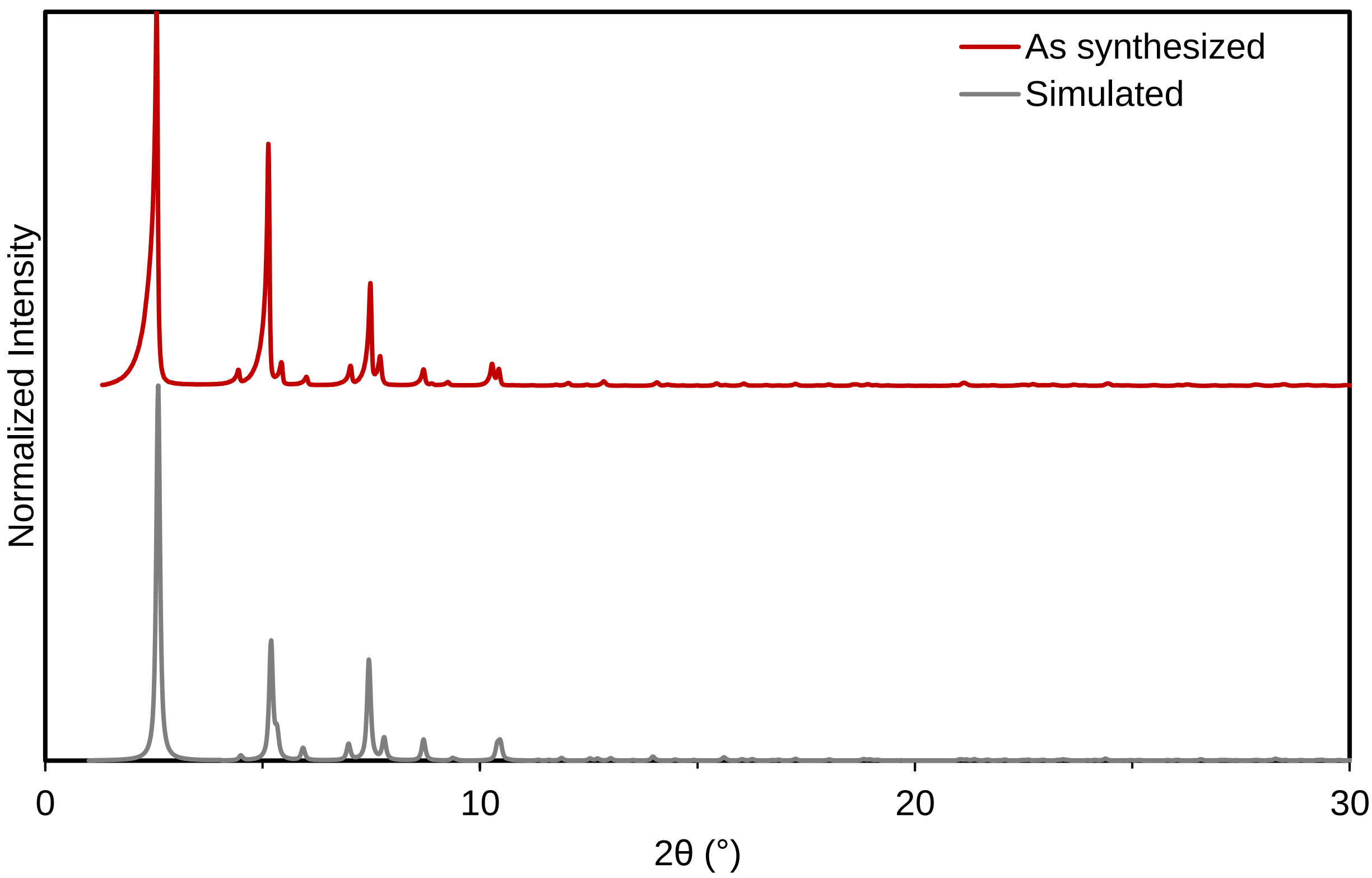

Figure S 4: PXRD pattern of NU-1000 after synthesis.

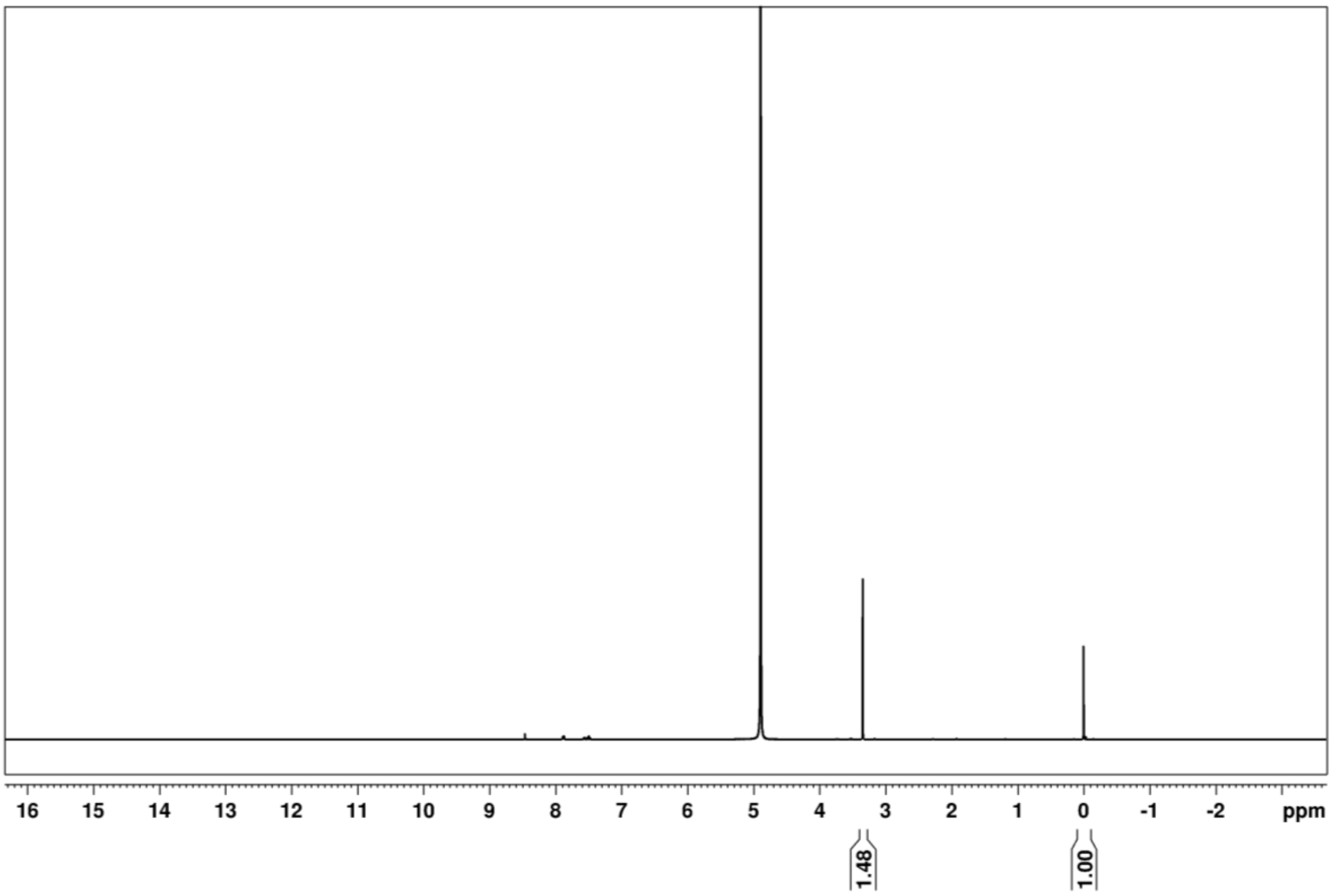

Figure S 5: ^{1}H-NMR spectra of NU-1000 after base digestion (1M NaOH, in D_2O). Some MeOH (3.35 ppm) leftover from the washing procedure remained present in the MOF structure after the activation. TMSP-d_4 (0 ppm) was used as an internal standard.

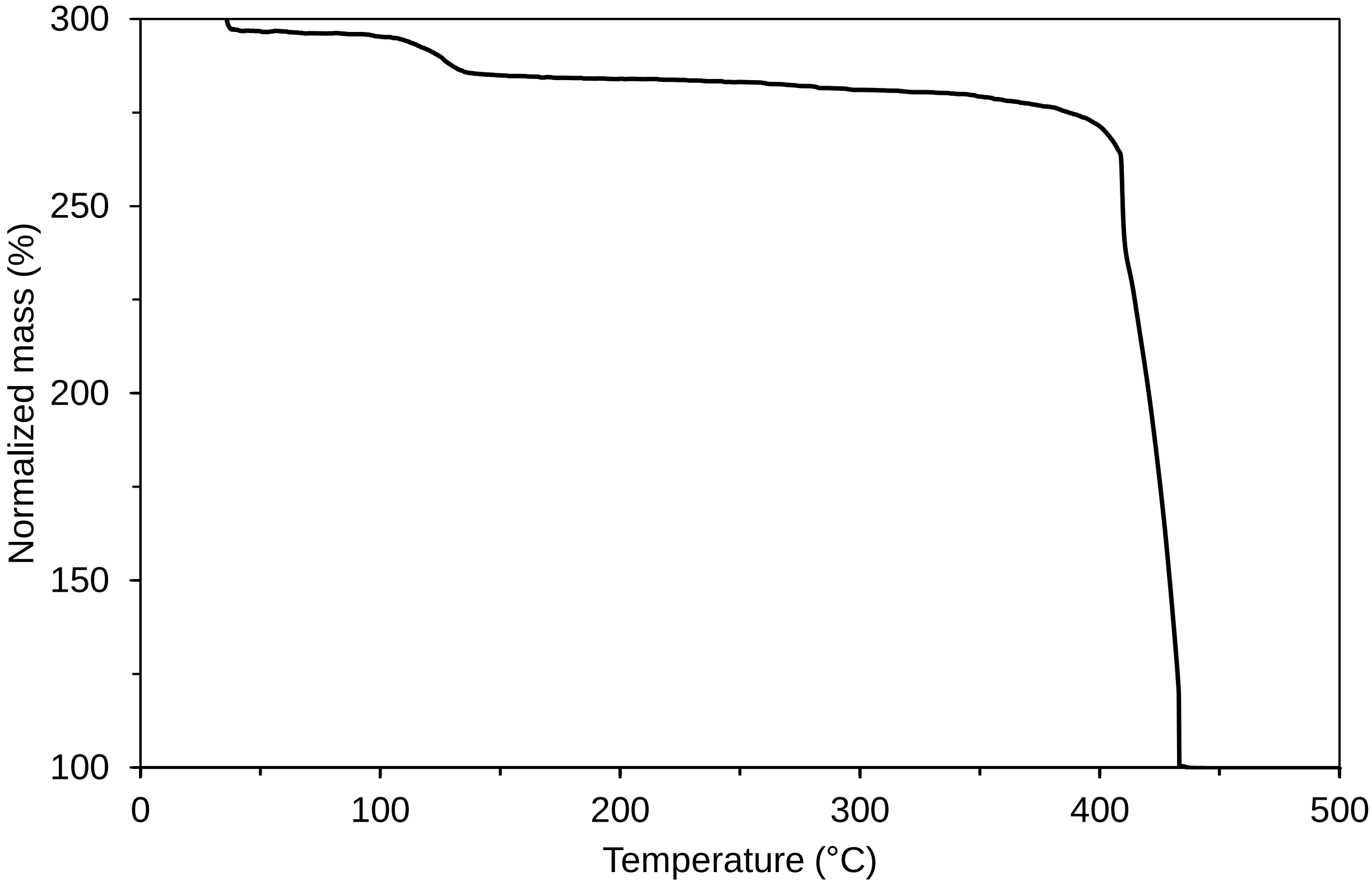

Figure S 6: Thermogravimetric analysis of NU-1000 after synthesis.

Table S 6: Overview of steps for estimating the missing linker content.

Start Material loss	End Material loss	Δ(End-Start)	#Total Missing Connections	#Missing connections per cluster	#Missing connections	#Missing Linkers
296	357	61	196	24.5	2.489796	0.622449

Of note, the missing linker content was estimated from the TGA curve according to the following description. The starting point of material loss from the MOF structure (when water or residual solvent loss is finished) and the percentage at which linker molecule loss in the curve starts are determined. The difference of these percentages corresponds to the number of missing connections in the synthesized MOF. Next, the weight loss percentages per connection of a Zr node is calculated, done by subtracting 100% from the starting point of material loss and divide this by the total amount of connections (i.e. a connectivity of 8 for NU-1000). The amount of missing connections per cluster is estimated by taking the difference between the start and end point of the TGA curve and dividing this by the calculated weight loss percentages per connection. Since NU-1000 consists of tetratopic linkers, we can calculate the amount of missing linkers by dividing the amount of connections by four (i.e. if one linker molecule is removed, this results in four different Zr-nodes in the structure that will have a missing linker).

An ideal NU-1000 MOF would have the general formula $[Zr_6(\mu_3\text{-}OH)_4(\mu_3\text{-}O)_4(H_2O)_4(OH)_4(TBAPy)_2]$ containing $[Zr_6(\mu_3\text{-}OH)_4(\mu_3\text{-}O)_4(H_2O)_4(OH)_4]^{8+}$ nodes where eight of the twelve coordination sites are occupied by $TBAPy^{4-}$ linkers [H_4TBAPy = 1,3,6,8-tetrakis(p-benzoic-acid)pyrene (see **Figure 1** for visualization). The TGA-derived value of 0.6 therefore refers to an average of approximately 0.6 missing TBAPy linker equivalents per Zr_6 node, corresponding to about 30 % linker deficiency relative to the ideal stoichiometry. The average linker content can thus be expressed approximately as $Zr_6(TBAPy)_{1.4}$, with the remaining coordination sites terminated by capping species.

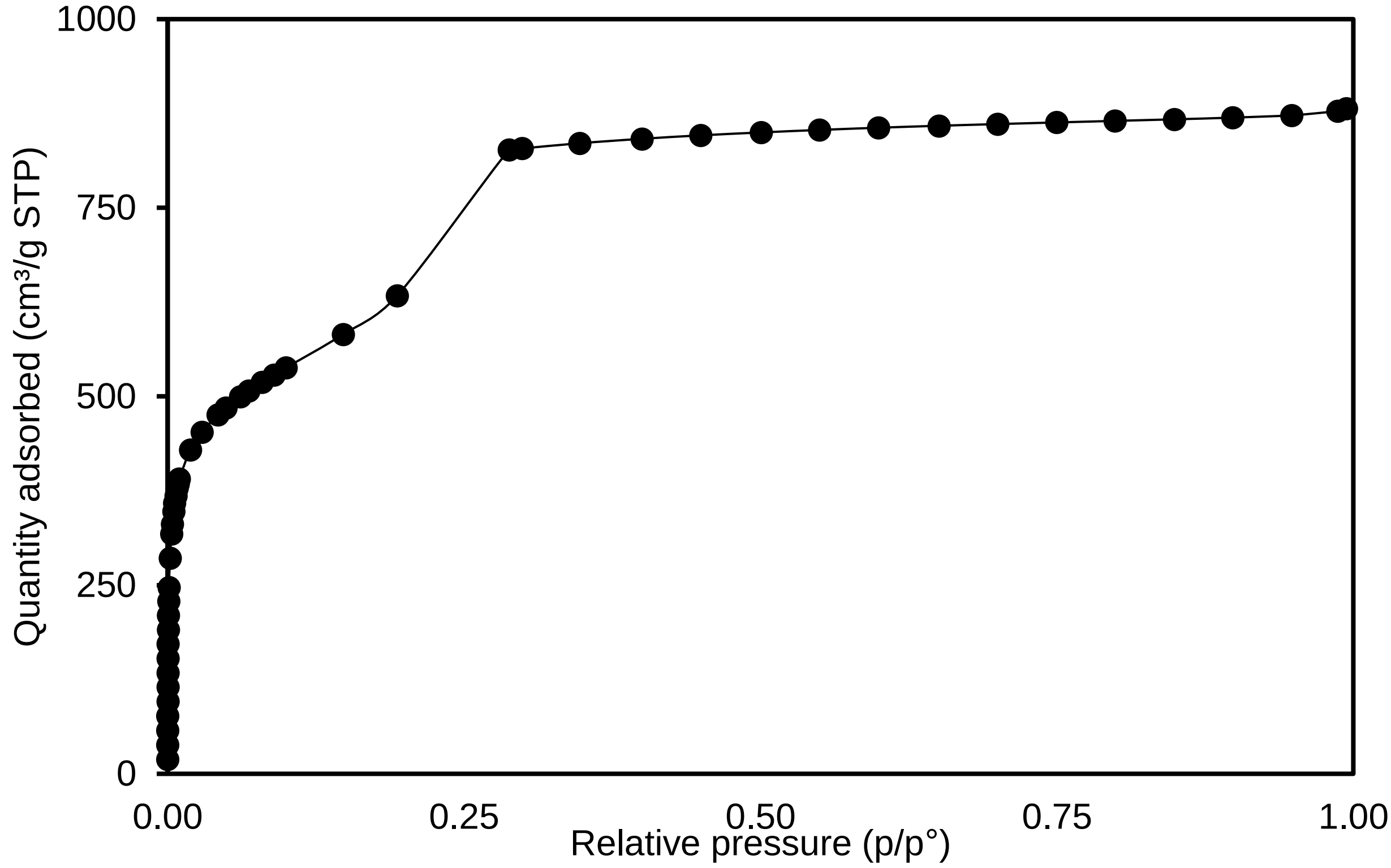

Figure S 7: N_2-physisorption isotherms of NU-1000 after synthesis.

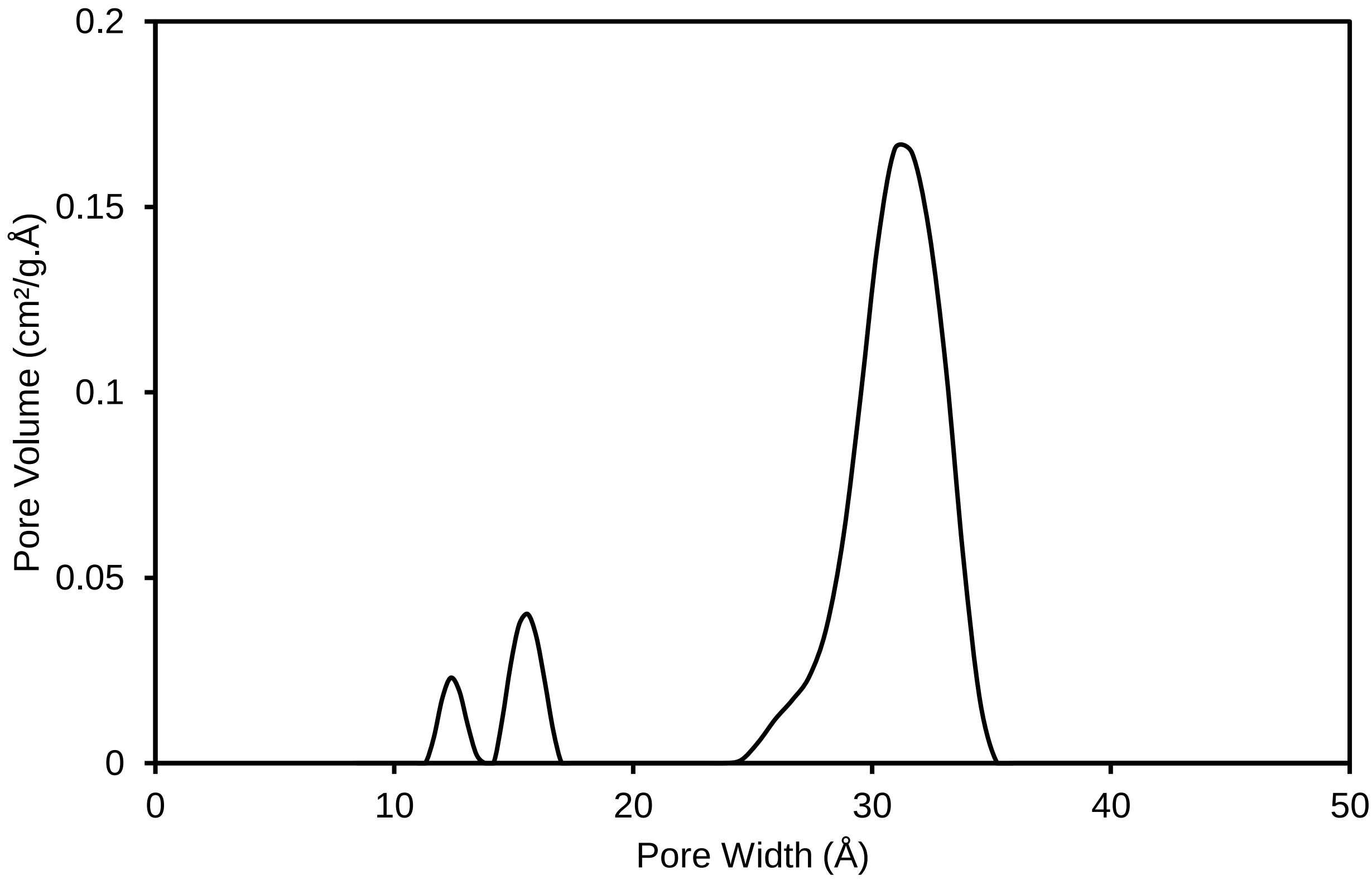

Figure S 8: Pore size distribution of NU-1000 after synthesis.

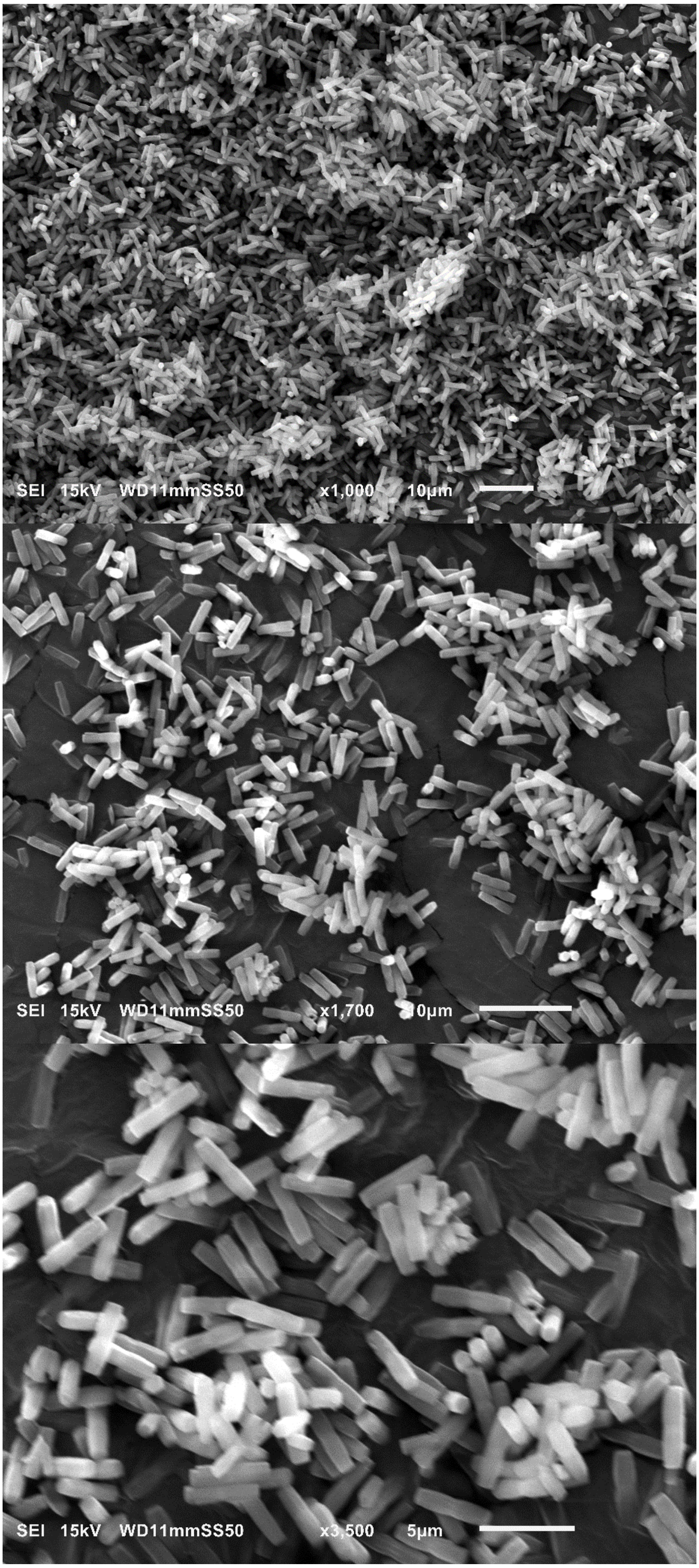

Figure S 9: SEM images of NU-1000 after synthesis.

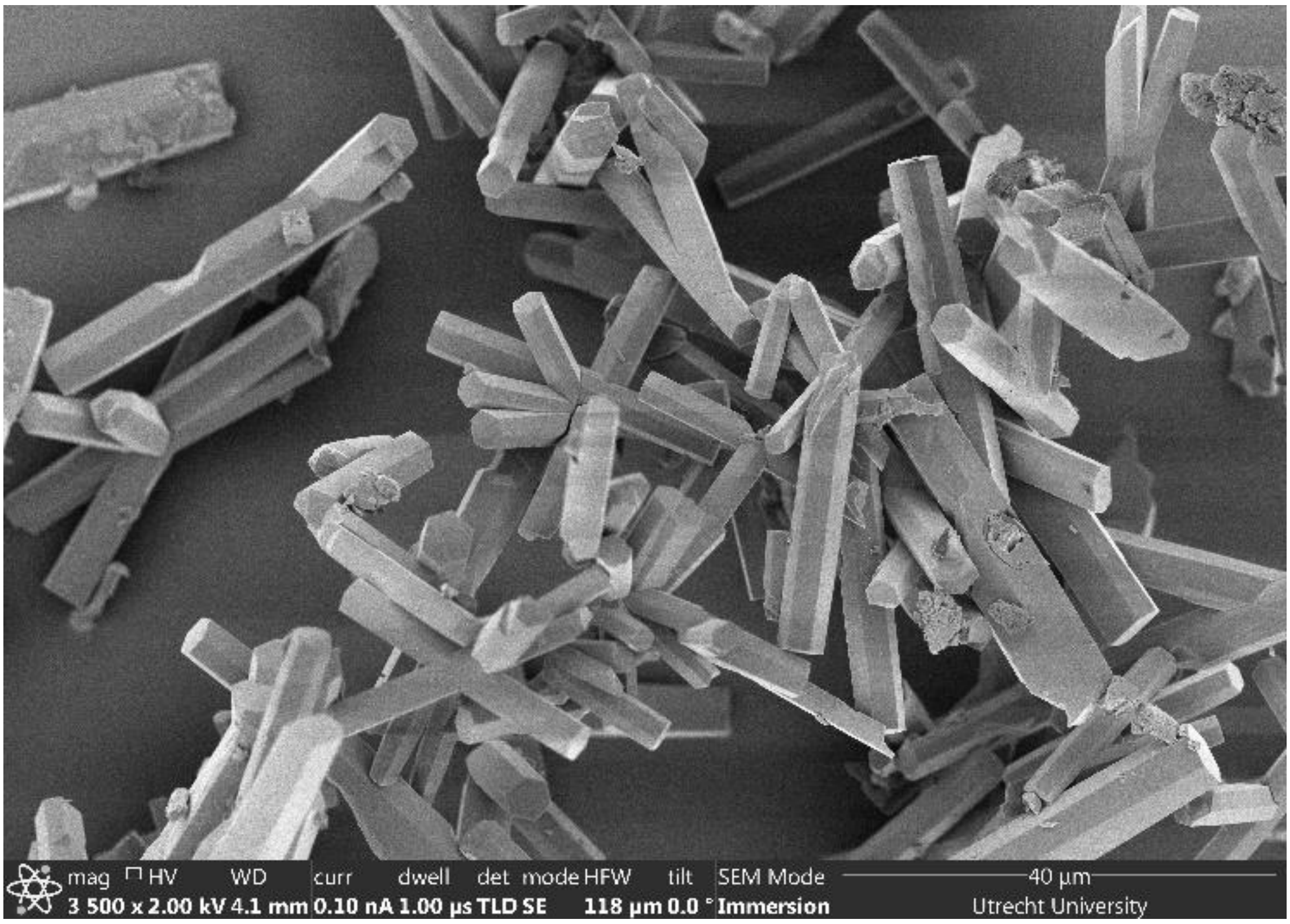

Figure S 10: Representative SEM micrograph of PCN-222 crystals used in the Cyt c uptake experiments. The crystals exhibit an elongated rod-like morphology. The total crystal length, ℓ, was determined from the long-axis dimension and was used to calculate the diffusion half-length, L=ℓ/2 = 21 +/- 7 µm.

Of note, this SEM micrograph was recorded with a EI Helios NanoLab G3 UC. MOF powders were deposited on Al stubs with carbon tape and sputter-coated with 10 nm of Pt, before the measurement. Beam currents of 0.1 nA and 2 kV were used to image the MOFs with a dwell time of 1 µs. External morphologies were imaged by collecting the secondary electrons (SE) with an Everhart–Thornley detector (ETD).

S10: MOF-protein interaction studies using FTIR-ATR spectroscopy and orthogonal experiments

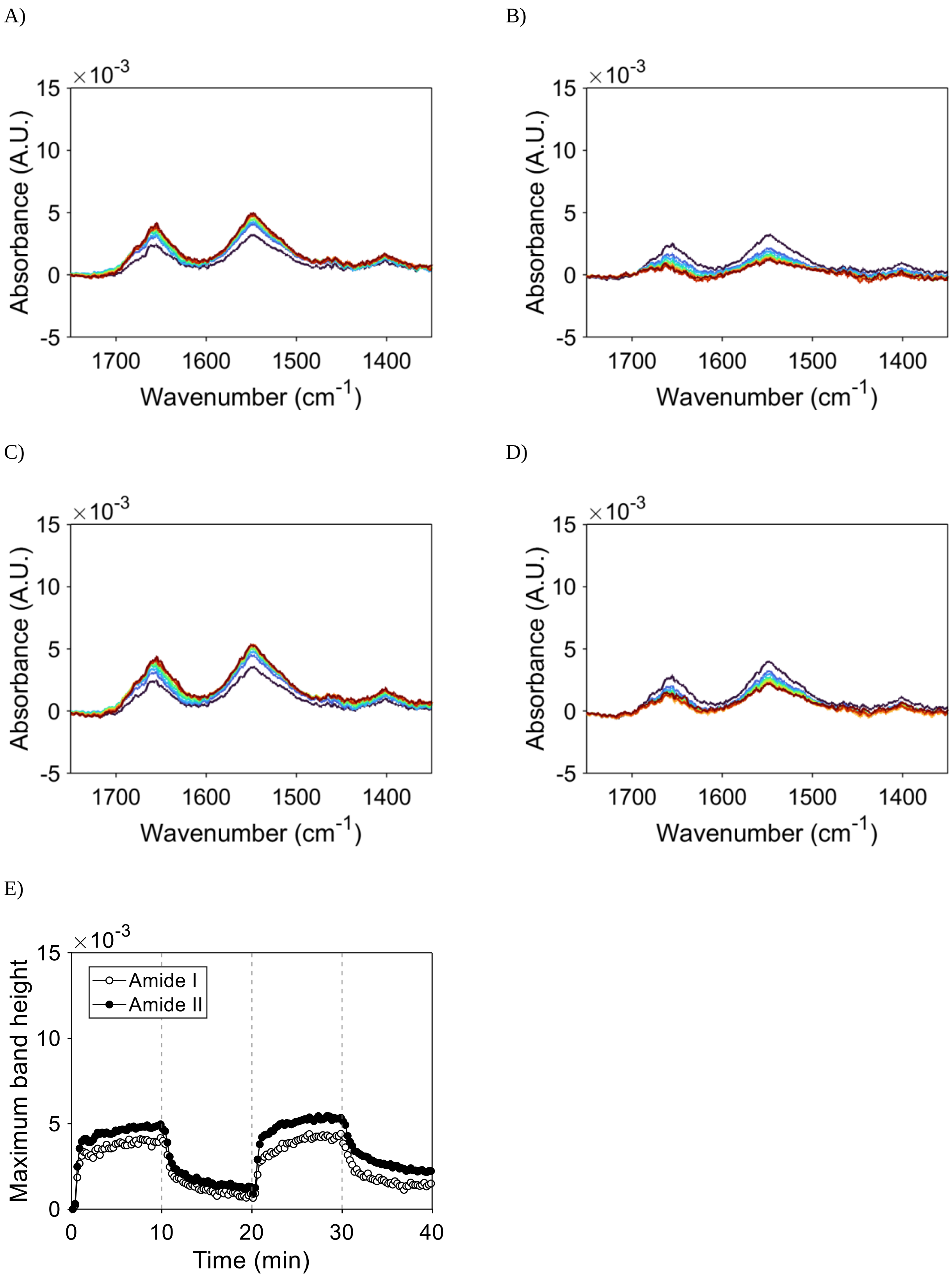

Figure S 11: **Averaged ATR-FTIR spectra of Cyt c on blank ATR crystal (pH 8.4, not corrected).** A) 20 µM protein application. B) Washing with MilliQ. C) 80 µM protein application. D) Washing with MilliQ. E). Maximum band height of amide I (~1650 cm^{-1}, white) and amide II (~1550 cm^{-1}, black) bands plotted against time. Starting spectra are depicted in blue and transform to red. The gray dashed lines indicate solutions swaps. Background spectrum**:** blank ATR crystal flushed with MilliQ.

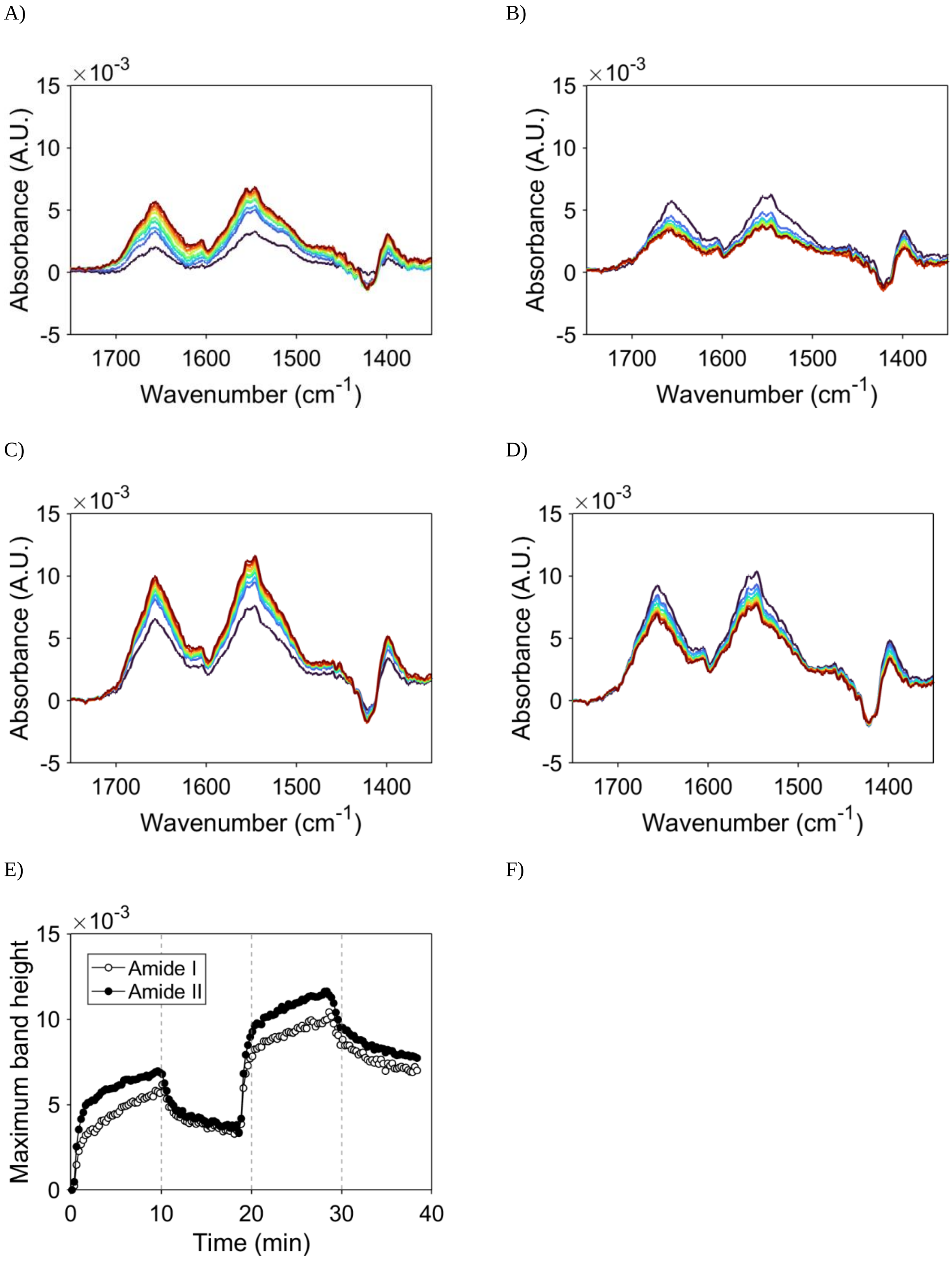

Figure S 12: **Averaged ATR-FTIR spectra of Cyt c on NU-1000 coated ATR crystal (pH 8.4, not corrected).** A) 20 µM protein application. Note that besides changes in the symmetric COO^- vibration around 1415 cm^{-1}, an additional differential feature around the asymmetric vibration at 1605 cm^{-1} becomes visible. B) Washing with MilliQ. C) 80 µM protein application. D) Washing with MilliQ. E). Maximum band height of amide I (~1650 cm^{-1}, white) and amide II (~1550 cm^{-1}, black) bands plotted against time. F) 20 µM denatured protein application. Starting spectra are depicted in blue and transform to red. The gray dashed lines indicate solutions swaps. Background spectrum**:** NU-1000 coated ATR crystal flushed with MilliQ.

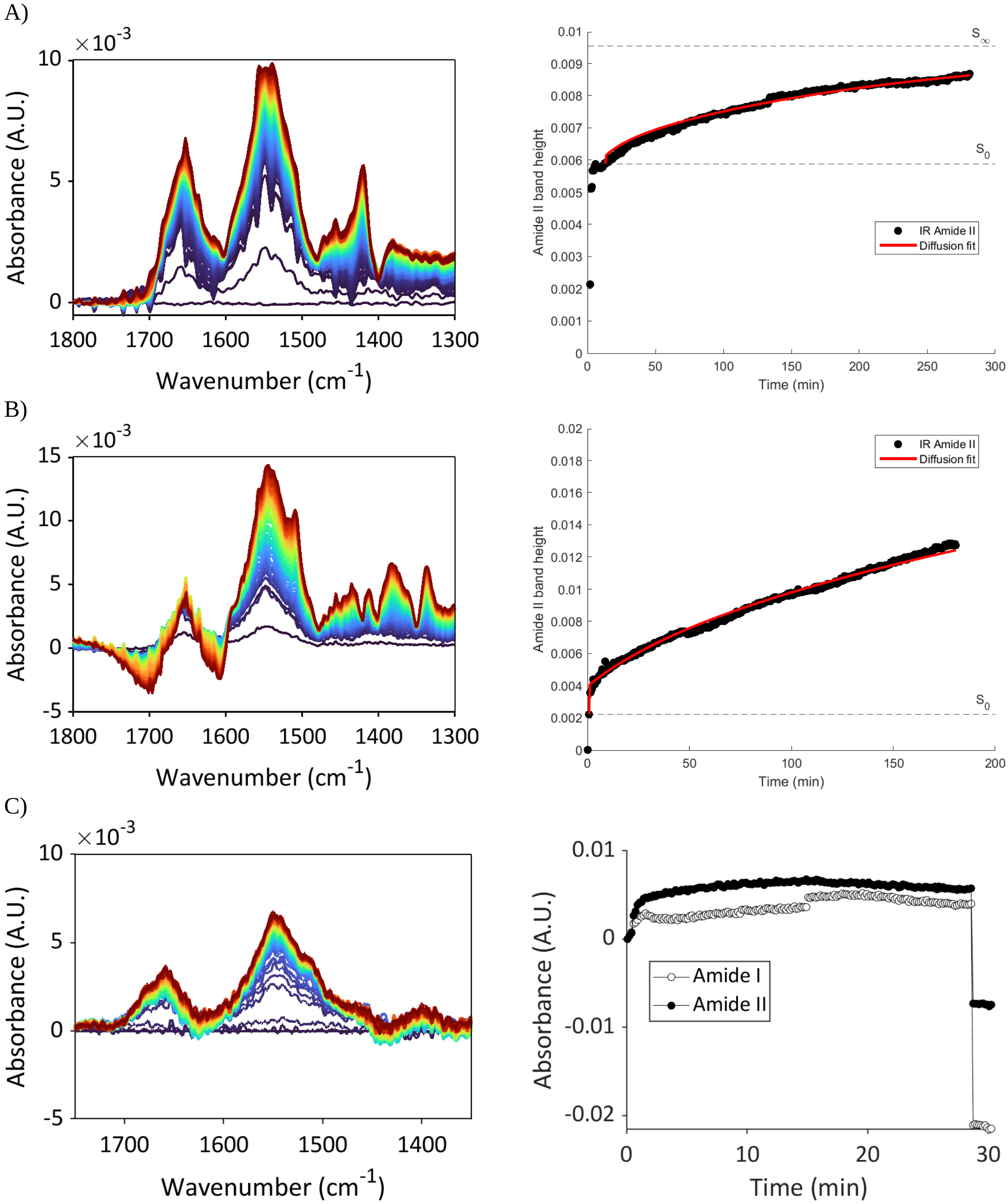

Figure S 13: Time-resolved IR spectra and amide band height recorded during Cyt c (0.3 mgmL^{-1}) uptake by (A) NU-1000 coated ATR crystal, (B) PCN-222 coated ATR crystal and (C) blank ATR crystal. Starting spectra are depicted in blue and transform to red. Background spectrum: MOF-coated ATR or blank ATR flushed with MilliQ. The black circles represent the experimental band heights, and the red lines represent nonlinear least-squares fits to the one-dimensional finite-length diffusion model.

Of note, the spectra of PCN-222 show multiple differential bands as PCN-222's linker shows multiple symmetric stretching vibrations. In contrast to NU-1000, interaction with PCN-222 appears to induce more pronounced changes in the Cyt c structure. Significant perturbations of the amide I band indicate substantial conformational rearrangement and are consistent with partial protein denaturation during uptake. This enhanced structural perturbation may also contribute to the comparatively faster uptake observed for PCN-222, as partial unfolding can facilitate access to the pore environment.

Table S 7: Results of apparent diffusion into PCN-222 and NU-1000.

	PCN-222	NU-1000
A_0	0.0022	0.0059
A_∞	0.092	0.0010
t_{50}	58.4 h	1.8 h
D_{app}	2.3 10^{-17} m^2 s^{-1}	7.5 10^{-16} m^2 s^{-1}

Of note, the fitted coefficients are referred to as apparent diffusion coefficients because the present model does not independently resolve intracrystalline diffusion, surface barriers, external mass-transfer resistance, crystal defects, or variations in crystal dimensions. The adsorption experiments for these fits were performed on NU-1000 and PCN-222 coated ATR crystals using a Cyt c concentration of 0.3 mg mL^{-1}. The corresponding time-resolved IR spectra are shown in **Figure S13,** as well as the amide II band heights extracted from these spectra were fitted using the diffusion model described above.

Table S 8: Overview of ICP-OES and Bradford assay results for Cyt c immobilized onto NU-1000, followed by trypsin cleavage and MOF digestion.

Samples Cyt c	Immobilization time (h)	Zr (ppm)	Fe (ppm)	S (ppm)	Fe (%)	S (%)	Bradford (%)
**Stock solution**	0	-0.10	0.98	2.64	100	100	100
**A) pellet after protein adsorption**	2	242.27	0.42	2.43	43	92	62
	4	244.81	0.40	2.05	41	78	64
**B) supernatant of A**	2	-0.09	0.62	1.12	63	42	-
	4	-0.10	0.61	1.13	63	43	-
**C) pellet after trypsin**	2	245.34	0.13	10.07	13	381	-
	4	231.81	0.13	9.93	13	376	-
**D) supernatant of C**	2	-0.09	0.24	136.06	24	5148	-
	4	-0.09	0.24	140.58	24	5319	-
**E) second wash after trypsin**	2	-0.08	-0.01	-0.31	-1	-12	-
	4	-0.09	-0.02	-0.22	-2	-9	-
**Control: Cyt c + trypsin**	-	-0.09	1.10	171.23	-	-	-
**Control: trypsin**	-	-0.10	-0.02	162.68	-	-	-

Of note, the data was averaged based on the radial ICP-OES measurements, was background corrected, and finally corrected with the appropriate protein-free MOF-containing blank.

Table S 9: Overview of ICP-OES and Bradford assay results for BSA immobilized onto NU-1000, followed by trypsin cleavage and MOF digestion.

Samples BSA	Immobilization time (h)	Zr (ppm)	S (ppm)	S (%)	Bradford (%)
**Stock solution**	0	-0.10	27.56	100	100
**A) pellet after protein adsorption**	2	245.43	10.09	37	27
	4	240.06	9.72	35	29
**B) supernatant of A**	2	-0.10	15.35	56	-
	4	-0.10	16.12	58	-
**C) pellet after trypsin**	2	241.46	10.53	38	-
	4	250.07	11.03	40	-
**D) supernatant of C**	2	-0.10	156.30	567	-
	4	-0.10	157.09	570	-
**E) second wash after trypsin**	2	-0.09	-0.37	-1	-
	4	-0.09	-0.34	-1	-
**Control: BSA + trypsin**	-	-0.09	203.92	-	-
**Control: trypsin**	-	-0.10	162.68	-	-

Of note, in contrast to Cyt c, BSA does not contain a unique elemental marker that can be selectively distinguished from trypsin by ICP-OES: both BSA and trypsin contain sulfur-bearing amino acids. Sulfur quantification would therefore contain contributions from both proteins and could not be unambiguously assigned to retained BSA. We consequently refrained from using sulfur-based ICP-OES as a quantitative measure of BSA retention.

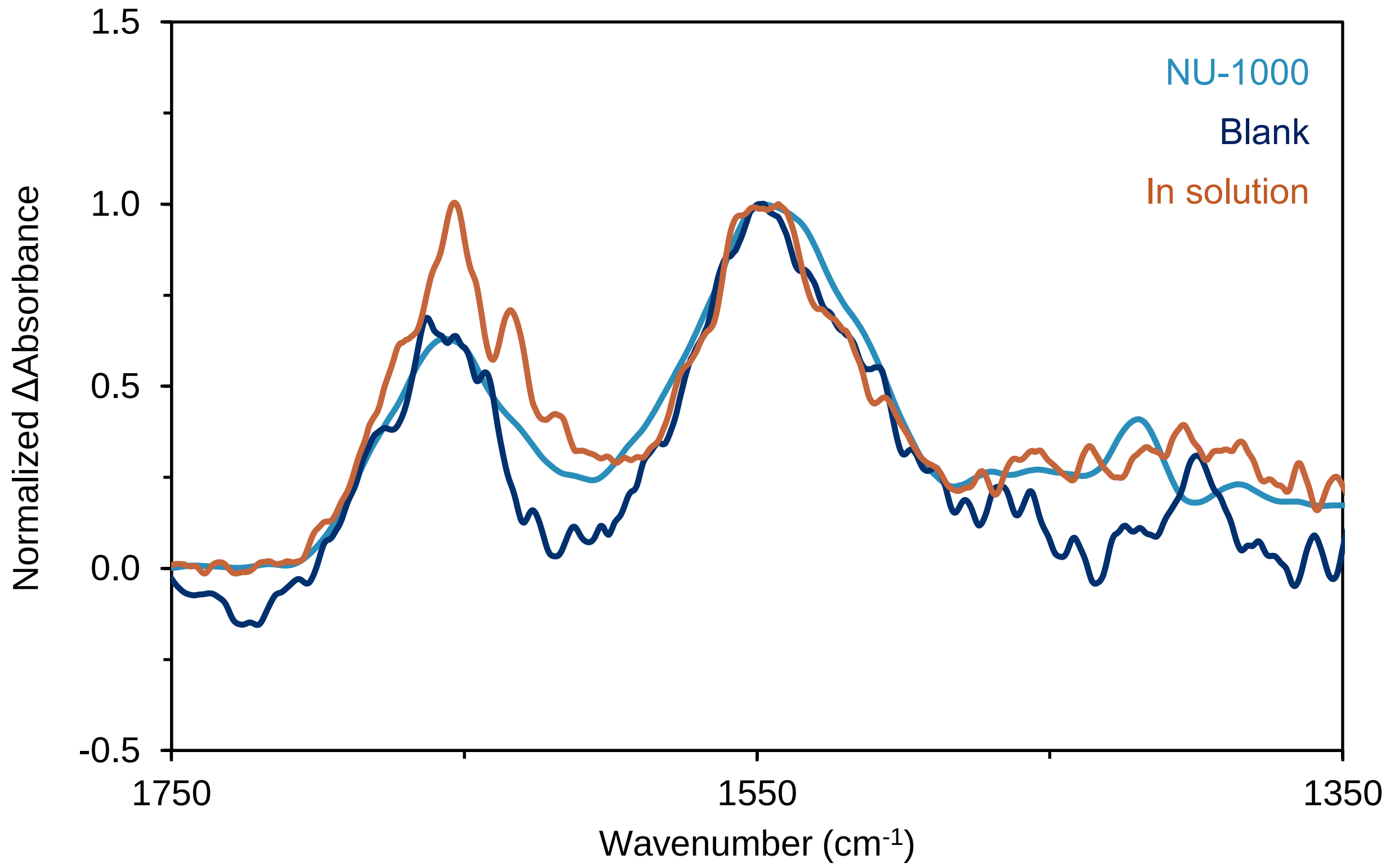

Figure S 14: Resulting normalized IR spectra of Cyt c (80 µM) blank and NU-1000 coated ATR crystals. The in solution spectrum was obtained by subtraction of Cyt c on the blank ATR crystal before at 25 min and after washing at 35 min. The spectra were normalized according to the amide II band.

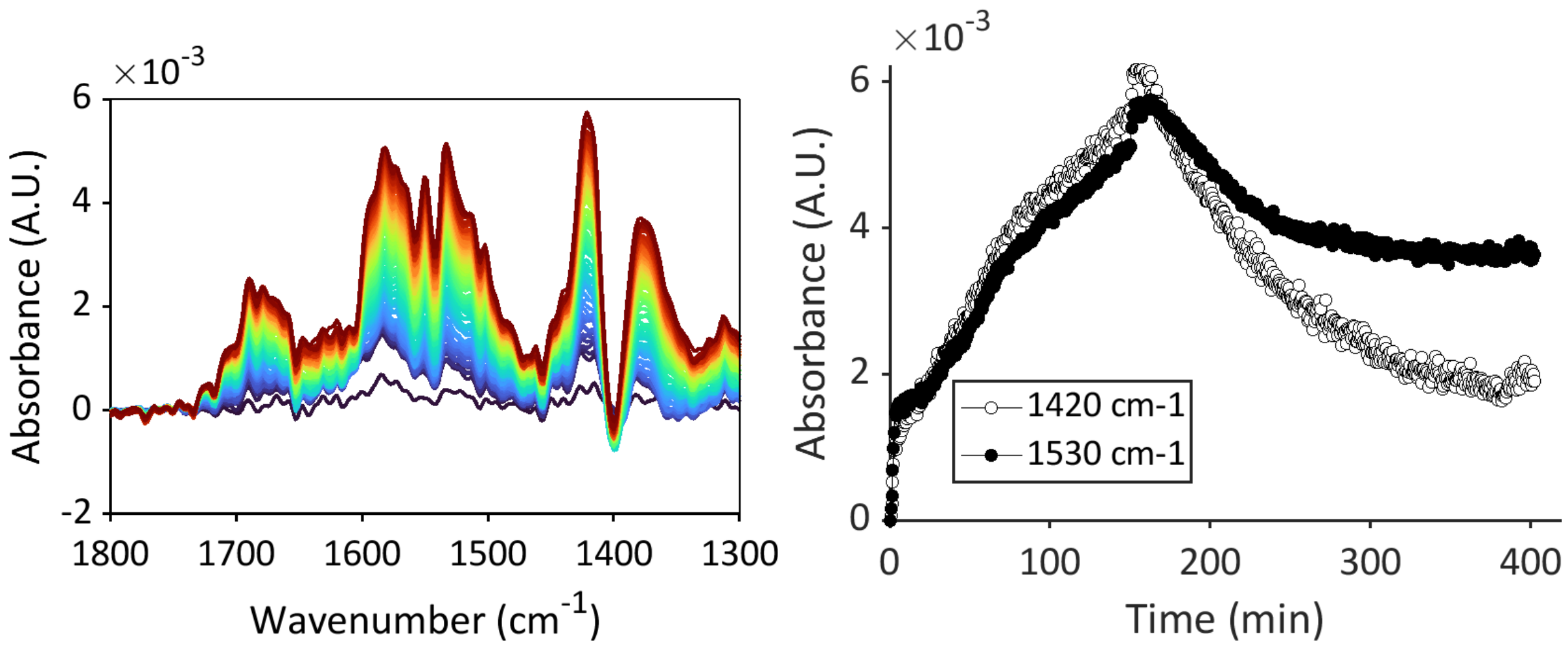

Figure S 15: ATR-FTIR spectra of 1.5 mM GlyGly on NU-1000 coated ATR crystal for 3 h, followed by 3 h of washing with MilliQ. Starting spectra are depicted in blue and transform to red. Background spectrum: NU-1000 coated ATR crystal flushed with MilliQ.

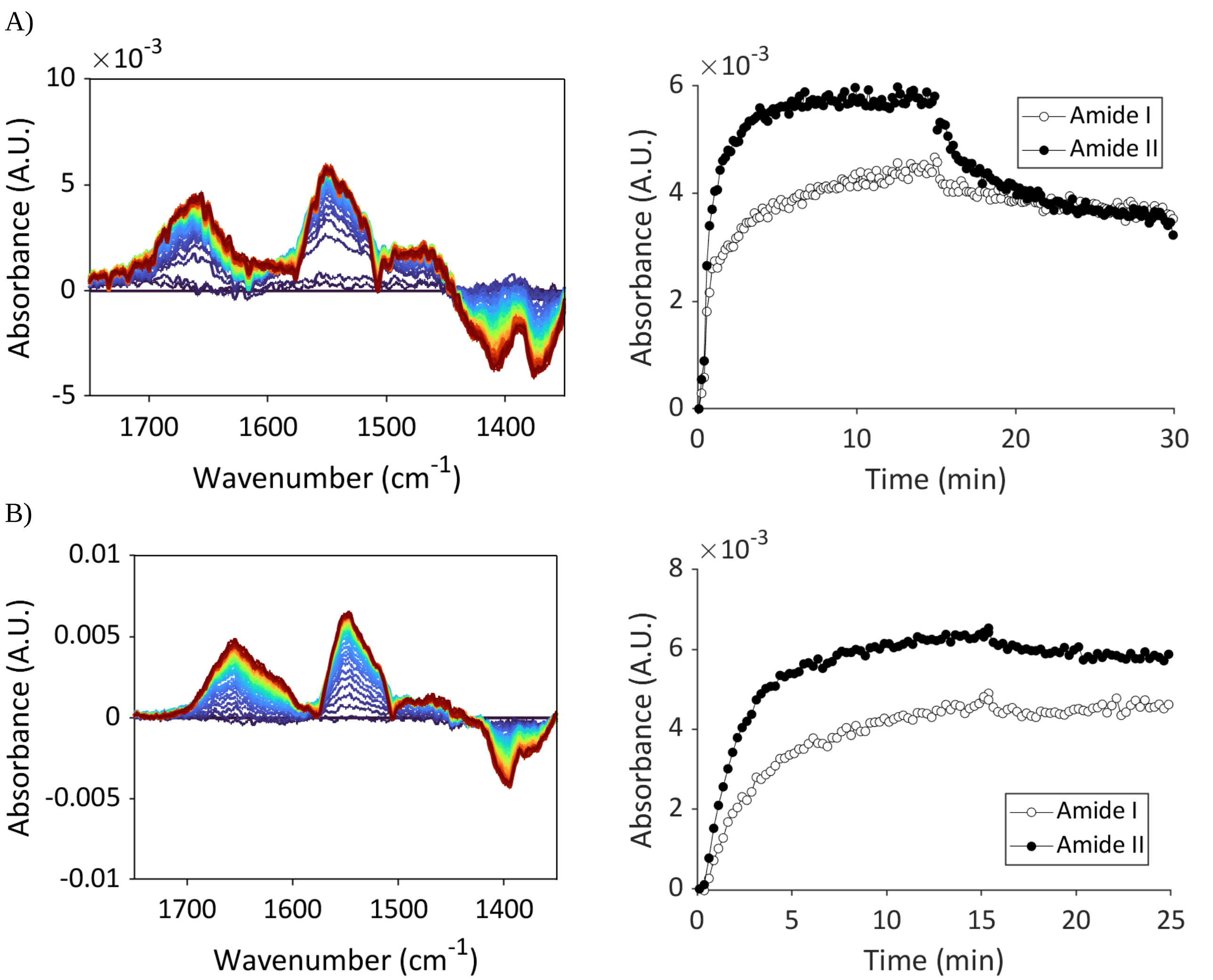

Figure S 16: Time-resolved IR spectra and amide band height recorded protein uptake by UiO-66 coated ATR crystal with (A) Cyt c (0.3 mgmL^{-1}) and (B) BSA (0.3 mgmL^{-1}). Starting spectra are depicted in blue and transform to red. Background spectrum: MOF-coated ATR or blank ATR flushed with MilliQ. The black circles represent the experimental band heights, and the red lines represent nonlinear least-squares fits to the one-dimensional finite-length diffusion model.

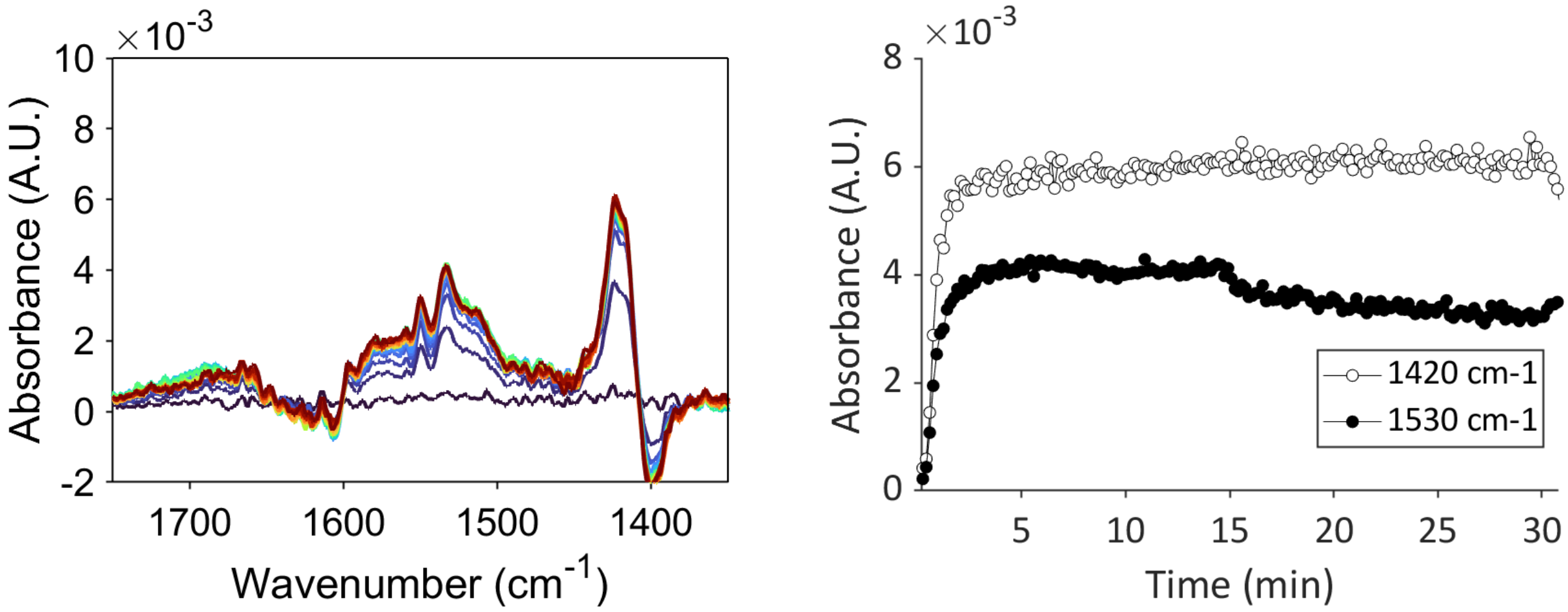

Figure S 17: Averaged ATR-FTIR spectra of 20 µM acid denatured Cyt c on NU-1000 coated ATR crystal. Starting spectra are depicted in blue and transform to red. Background spectrum: NU-1000 coated ATR crystal flushed with MilliQ.

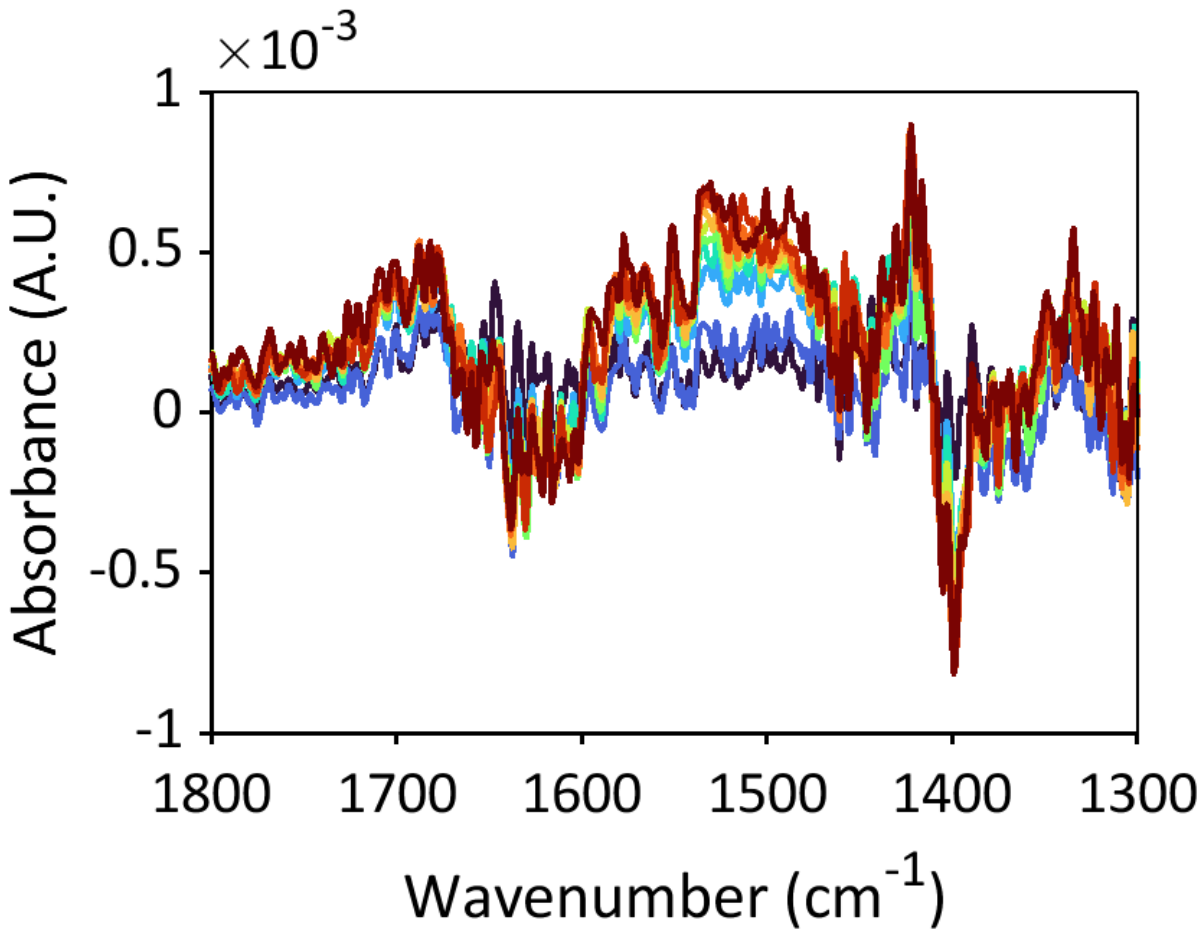

Figure S 18: Resulting ATR-FTIR spectra of flushing MilliQ water of pH 7 over an NU-1000 coated ATR crystal until a stable baseline was obtained, followed by changing to flushing MilliQ water that was corrected to pH 5 using HCl. Starting spectra are depicted in blue and transform to red. Background spectrum: NU-1000 coated ATR crystal flushed with MilliQ.

A)

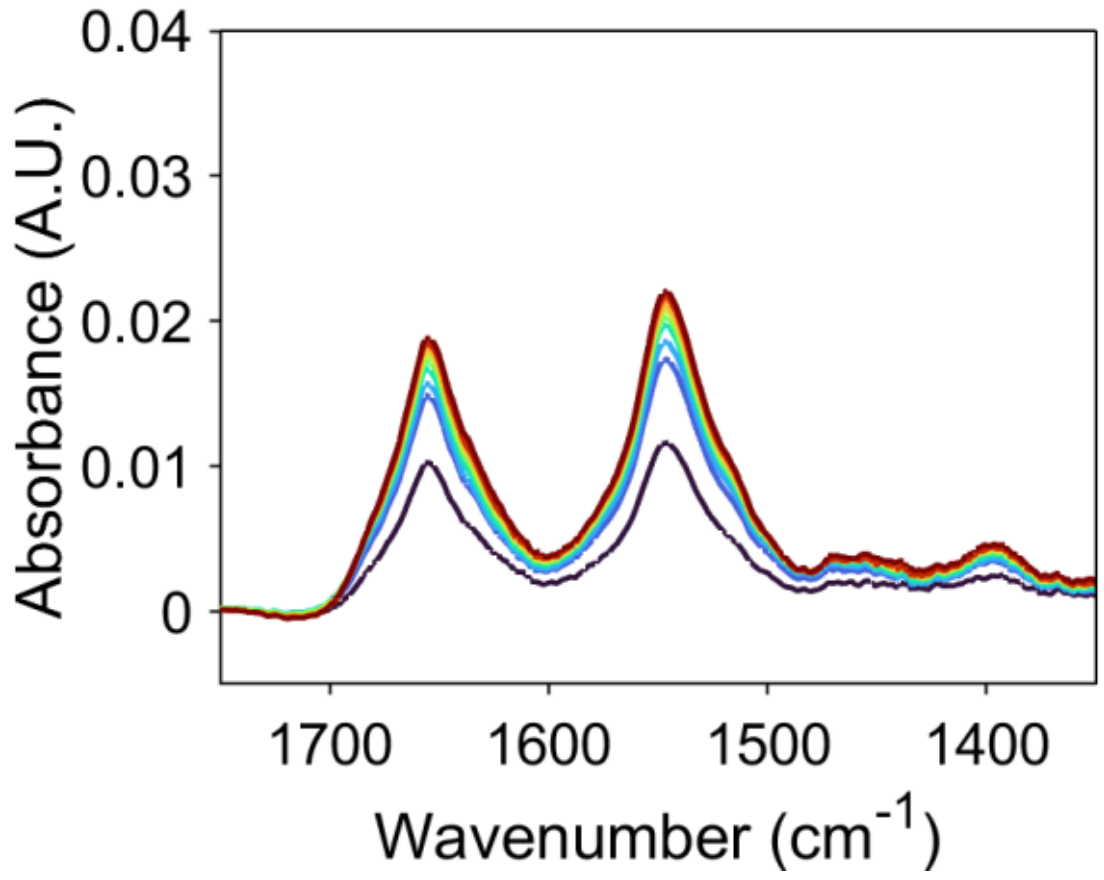

B)

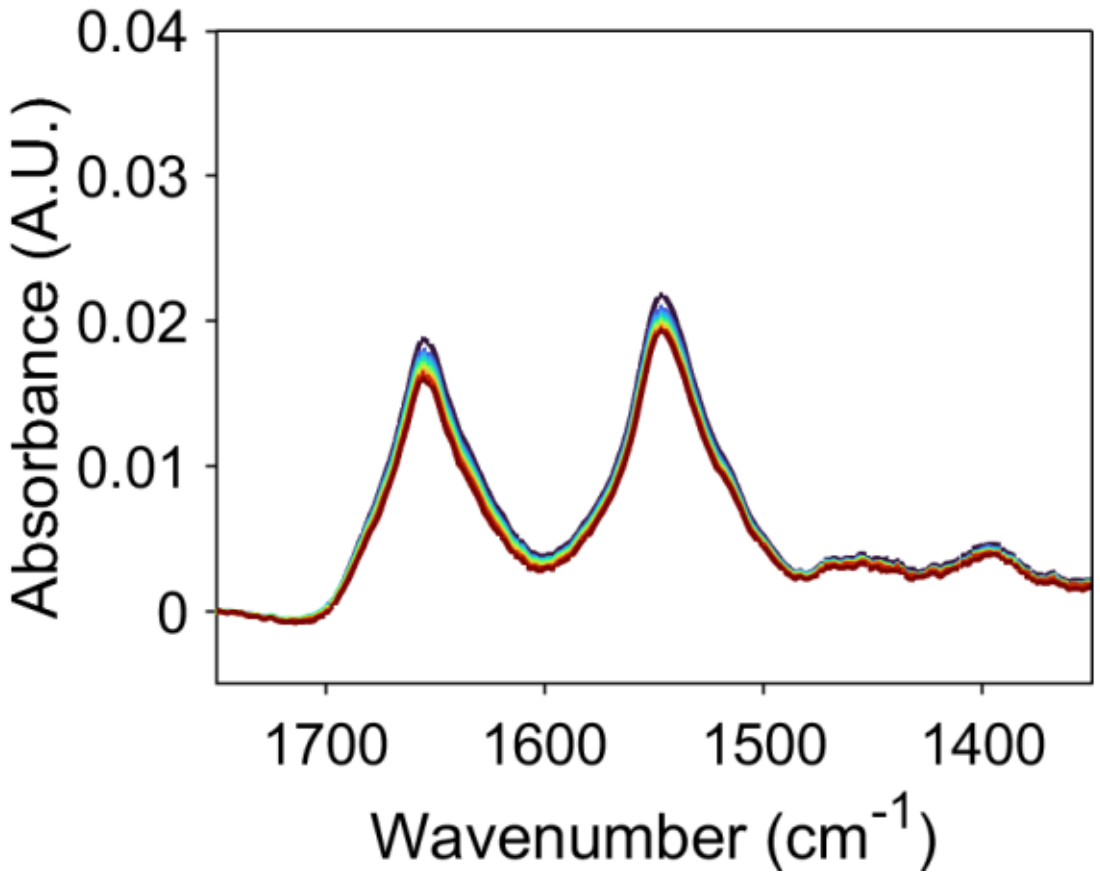

C)

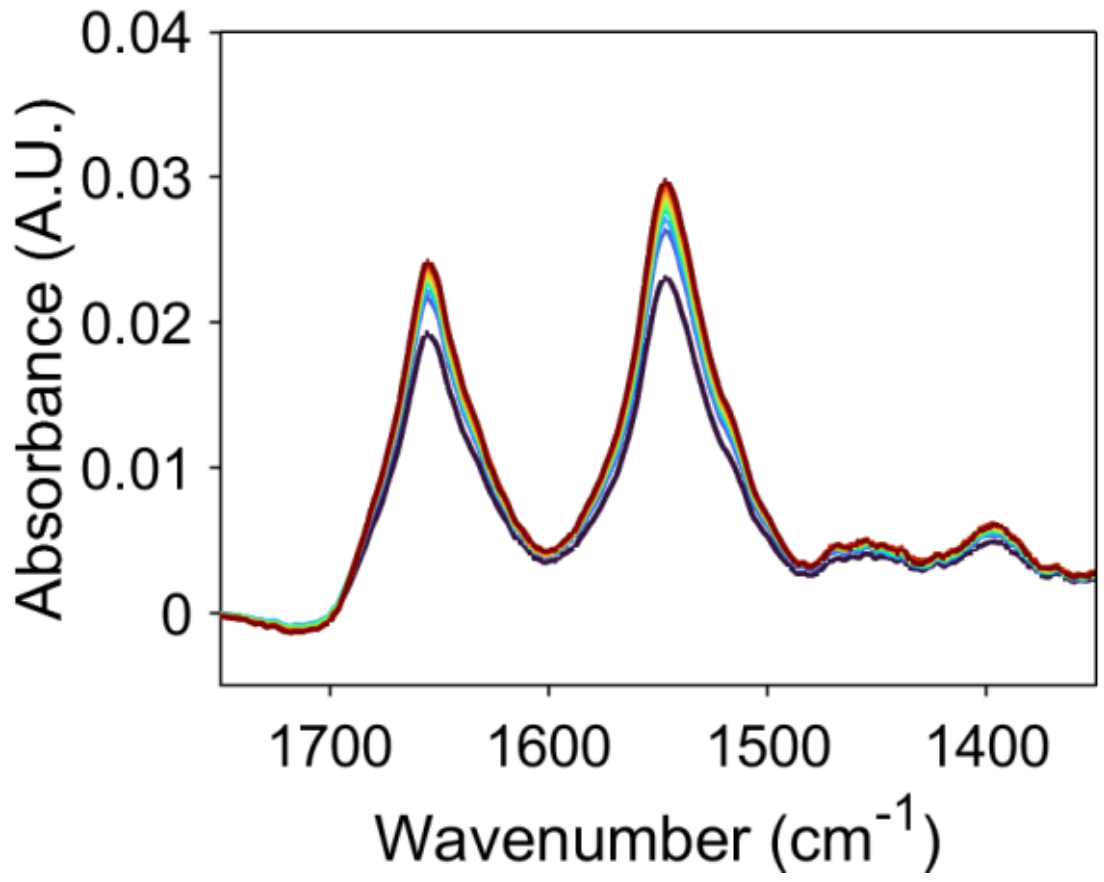

D)

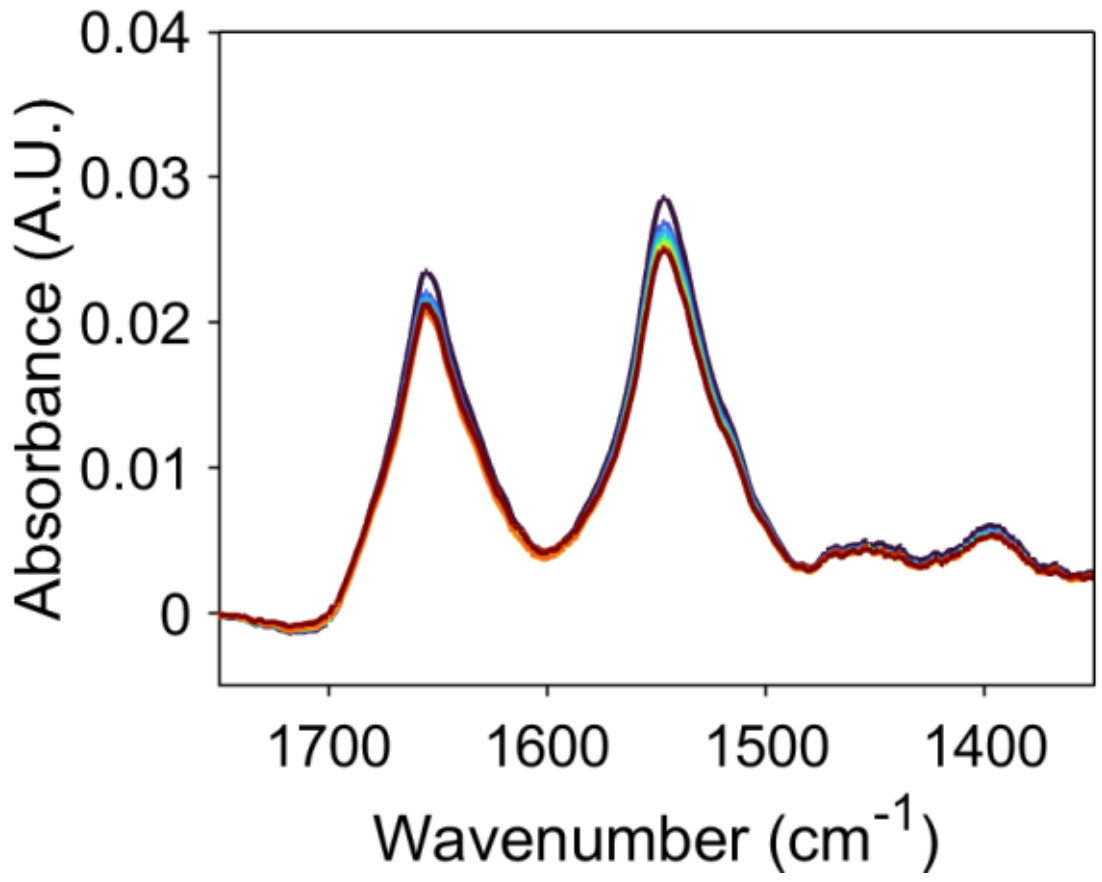

E)

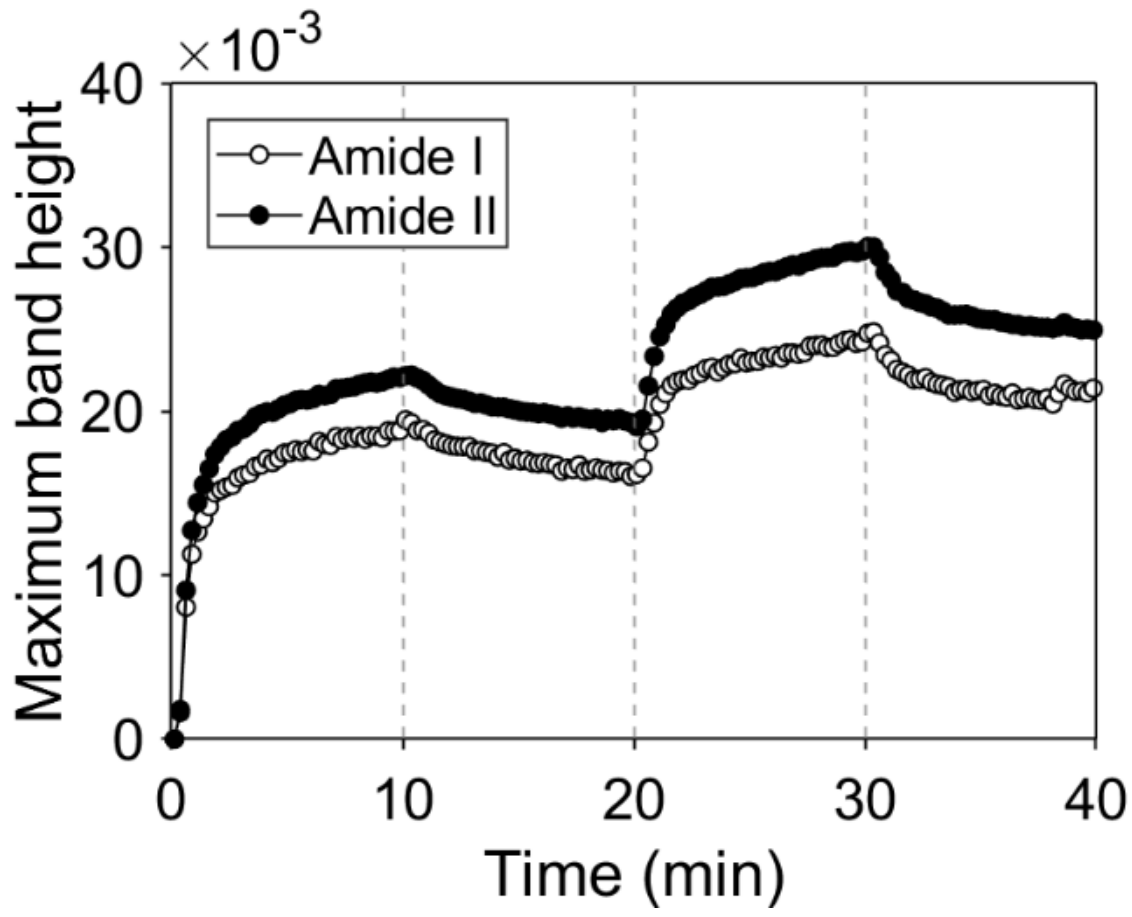

Figure S 19: **Averaged ATR-FTIR spectra of HB on blank ATR crystal (corrected to pH 7).** A) 20 µM protein application. B) Washing with MilliQ. C) 80 µM protein application. D) Washing with MilliQ. E). Maximum band height of amide I (~1650 cm^{-1}, white) and amide II (~1550 cm^{-1}, black) bands plotted against time. Starting spectra are depicted in blue and transform to red. The gray dashed lines indicate solutions swaps. Background spectrum**:** blank ATR crystal flushed with MilliQ.

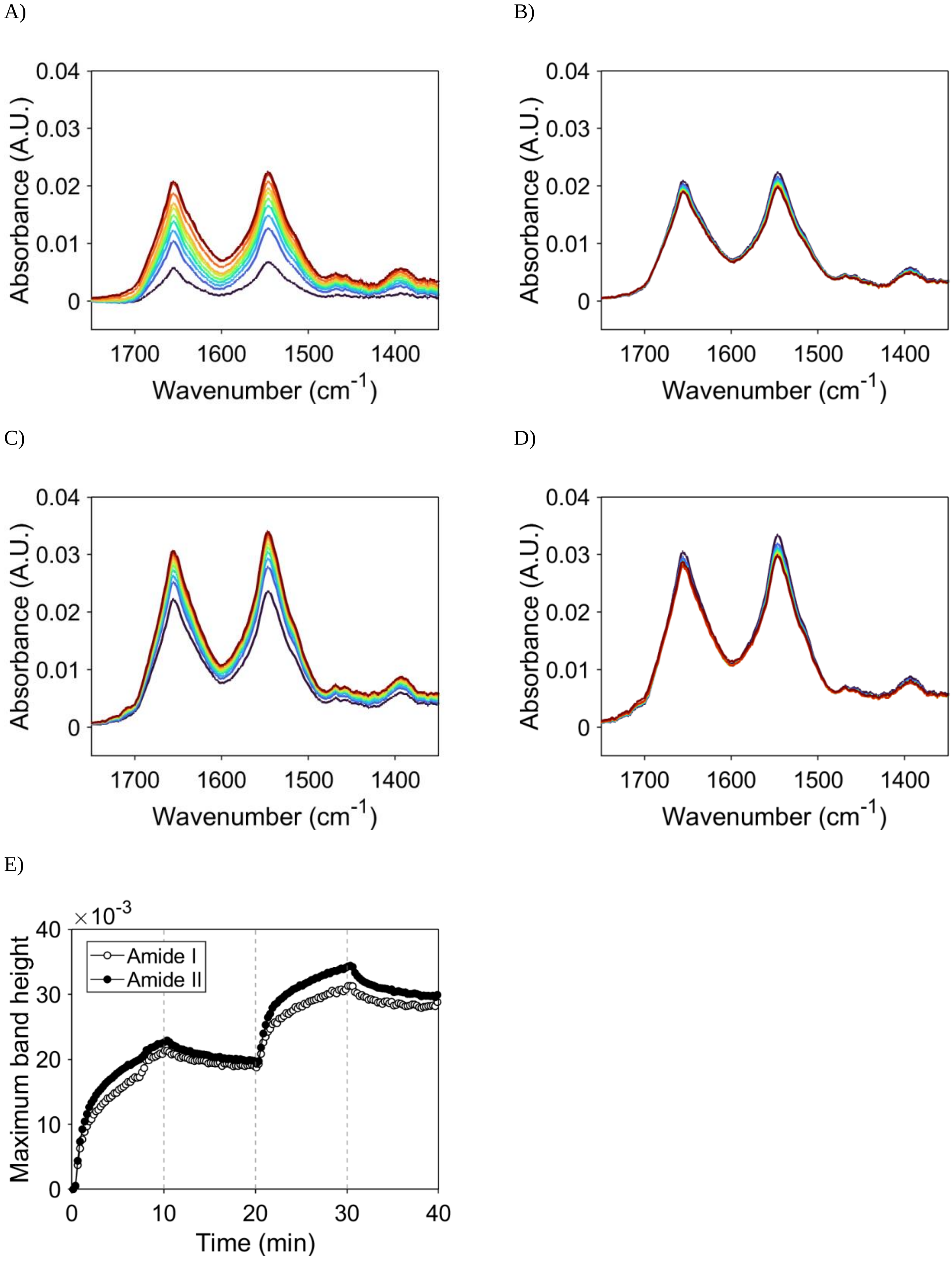

Figure S 20: **Averaged ATR-FTIR spectra of HB on NU-1000 coated ATR crystal (corrected to pH 7).** A) 20 µM protein application. B) Washing with MilliQ. C) 80 µM protein application. D) Washing with MilliQ. E). Maximum band height of amide I (~1650 cm^{-1}, white) and amide II (~1550 cm^{-1}, black) bands plotted against time. Starting spectra are depicted in blue and transform to red. The gray dashed lines indicate solutions swaps. Background spectrum**:** NU-1000 coated ATR crystal flushed with MilliQ.

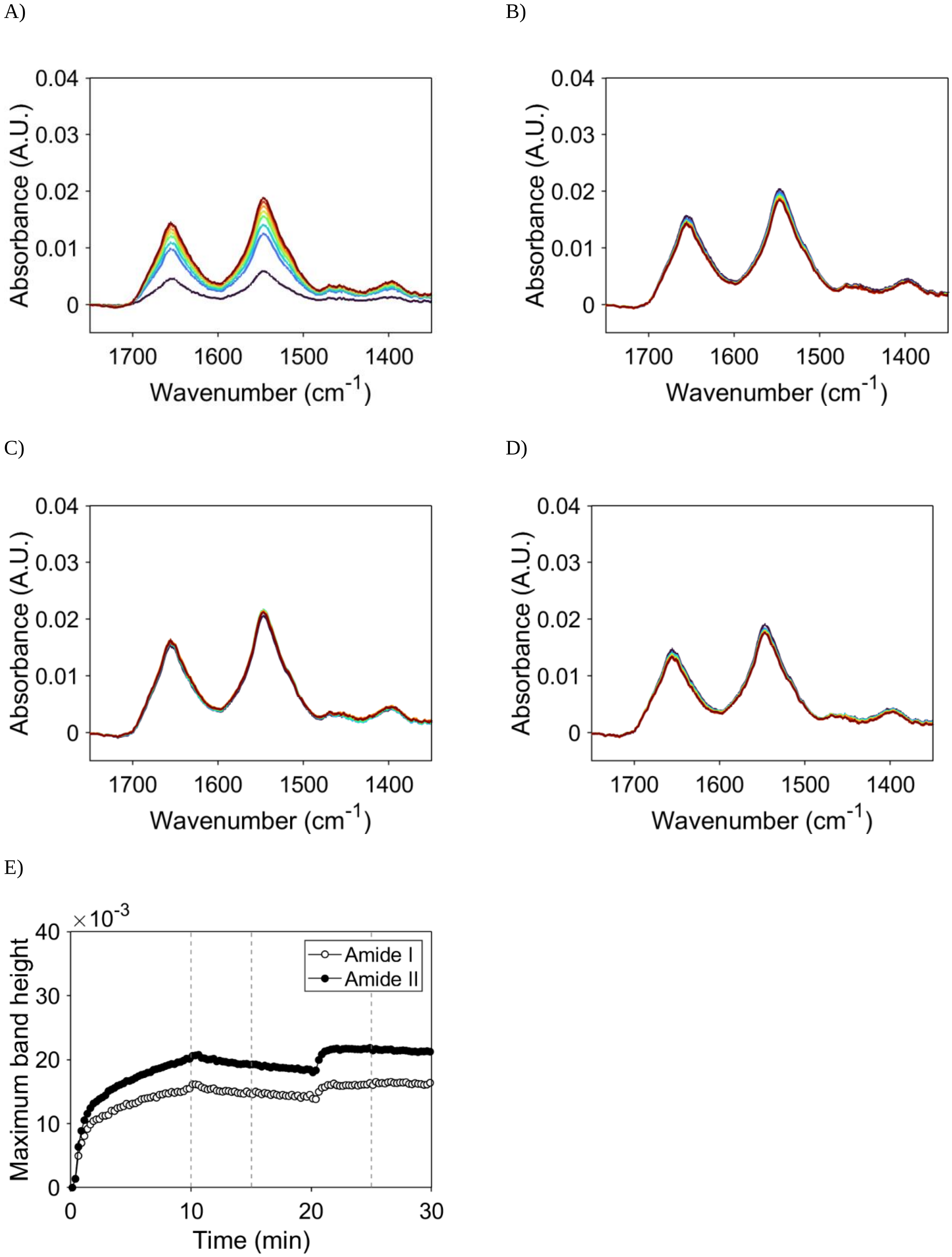

Figure S 21: **Averaged ATR-FTIR spectra of HB on blank ATR crystal (corrected to pH 5).** A) 20 µM protein application. B) Washing with MilliQ. C) 80 µM protein application. D) Washing with MilliQ. E). Maximum band height of amide I (~1650 cm^{-1}, white) and amide II (~1550 cm^{-1}, black) bands plotted against time. Starting spectra are depicted in blue and transform to red. The gray dashed lines indicate solutions swaps. Background spectrum**:** blank ATR crystal flushed with MilliQ.

A)

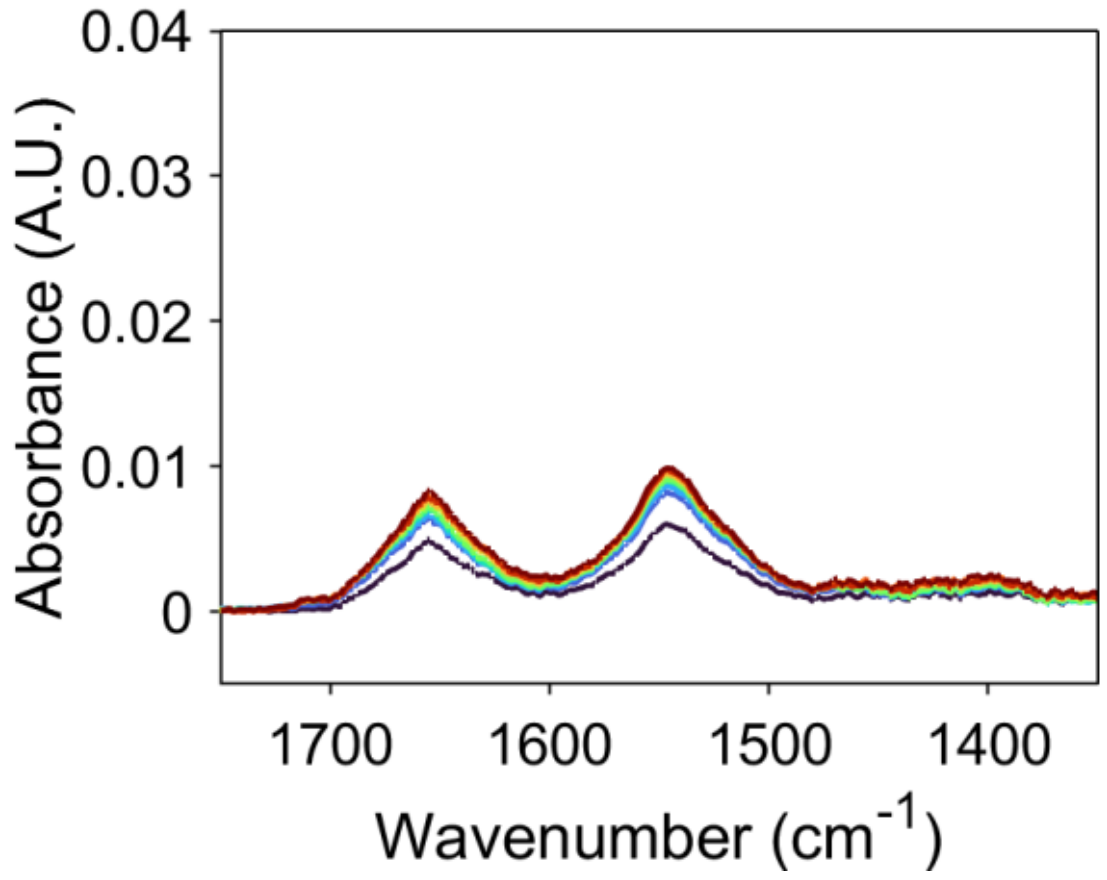

B)

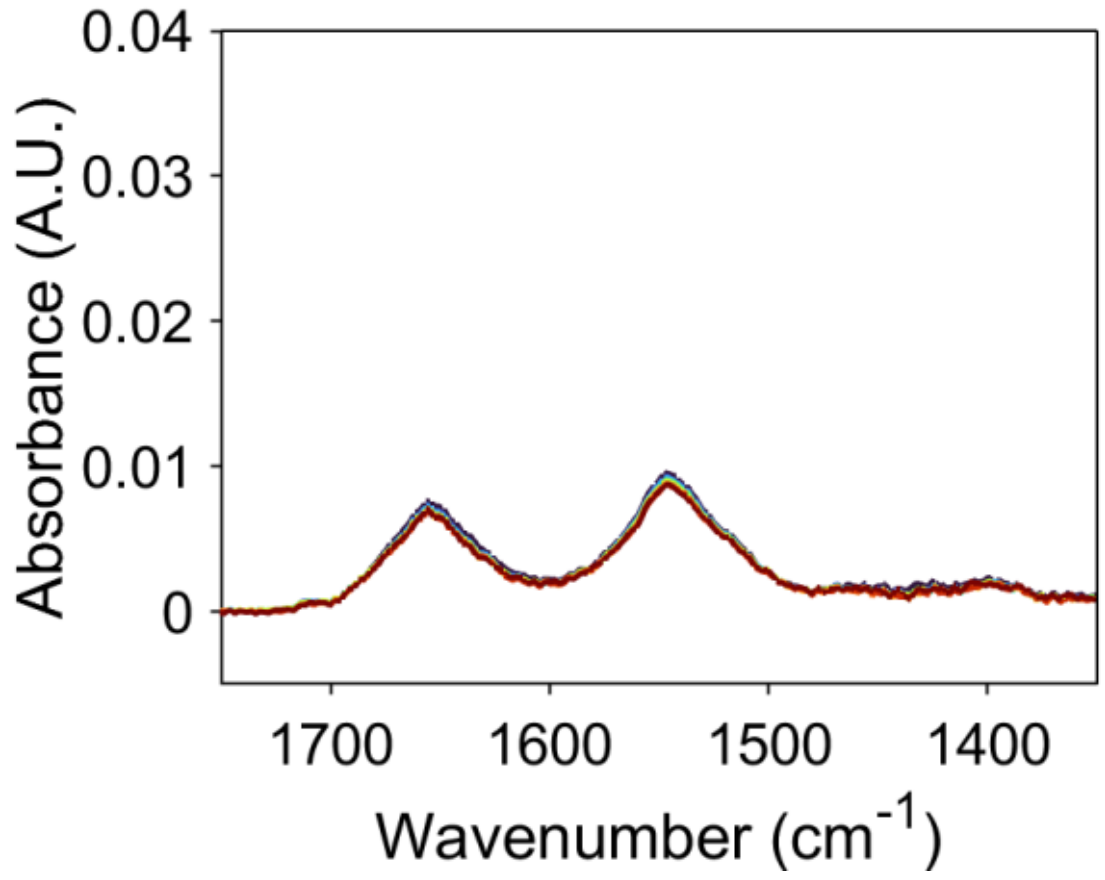

C)

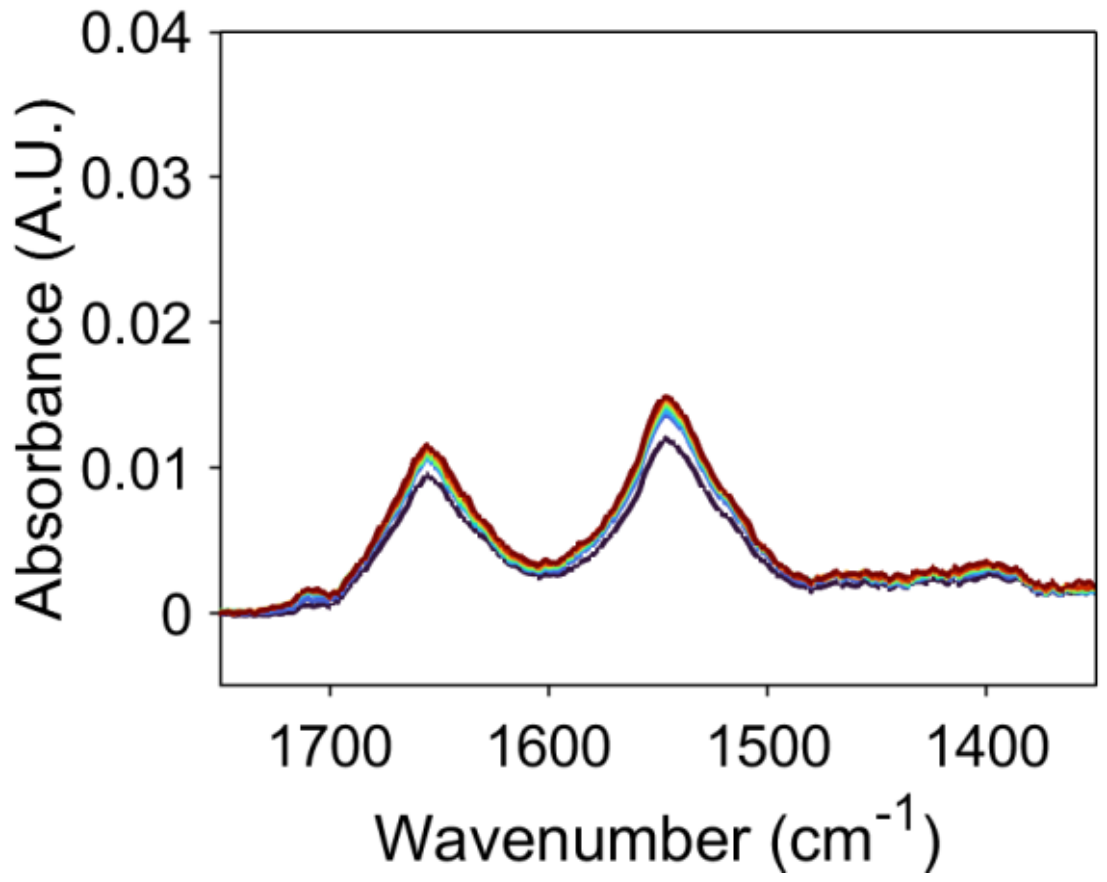

D)

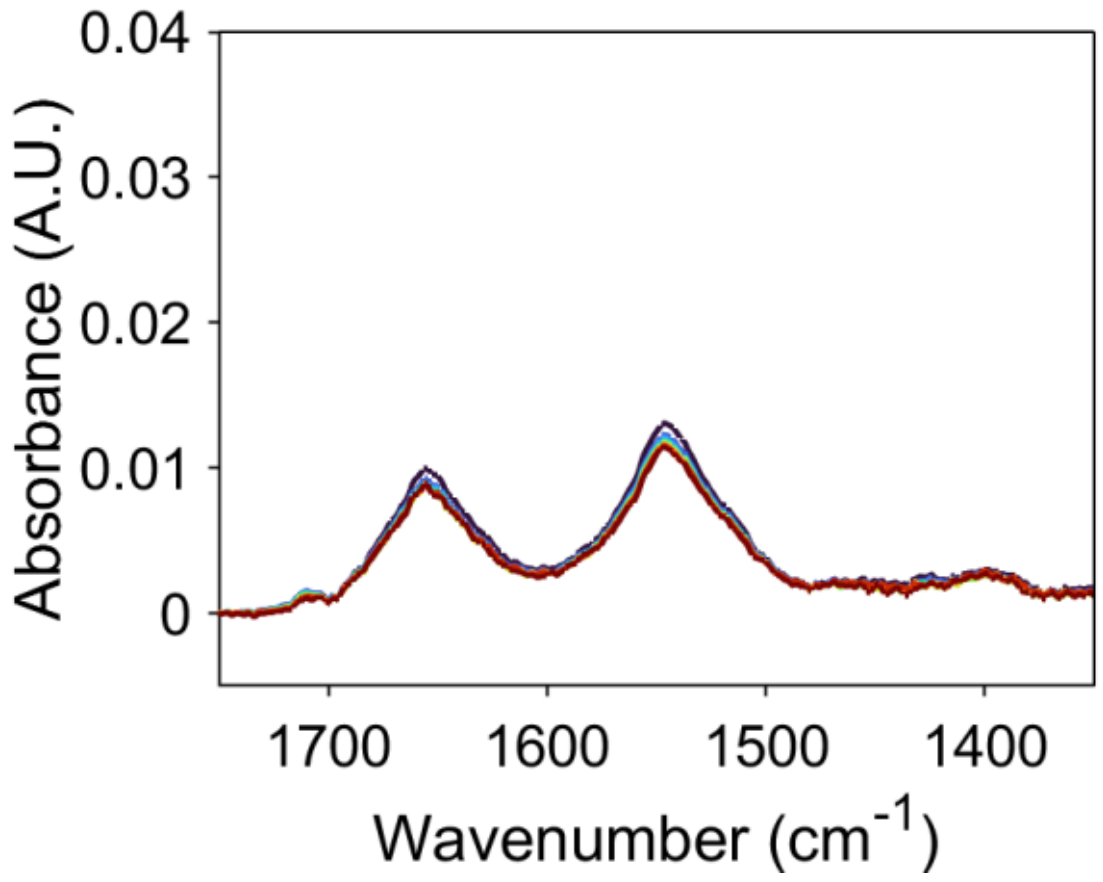

E)

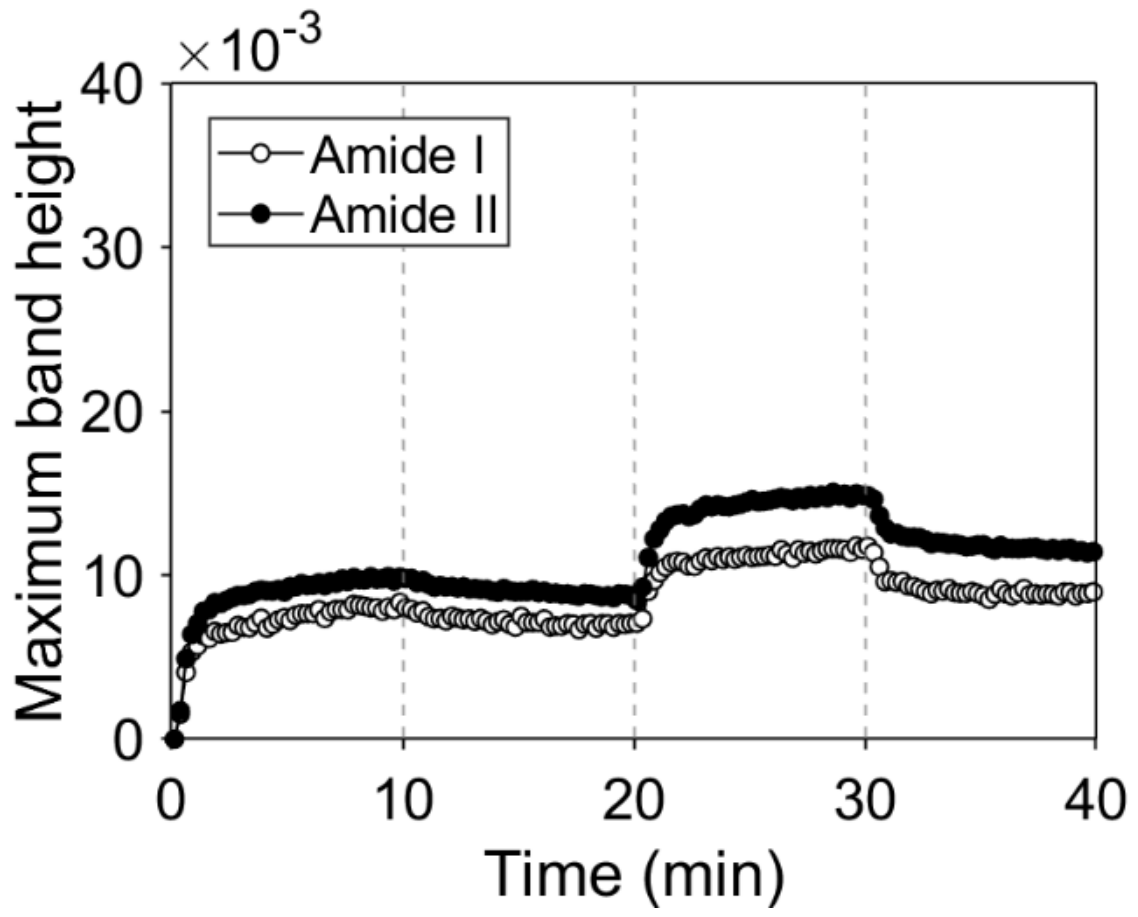

Figure S 22: **Averaged ATR-FTIR spectra of HB on NU-1000 coated ATR crystal (corrected to pH 5).** A) 20 µM protein application. B) Washing with MilliQ. C) 80 µM protein application. D) Washing with MilliQ. E). Maximum band height of amide I (~1650 cm^{-1}, white) and amide II (~1550 cm^{-1}, black) bands plotted against time. Starting spectra are depicted in blue and transform to red. The gray dashed lines indicate solutions swaps. Background spectrum**:** NU-1000 coated ATR crystal flushed with MilliQ.

A)

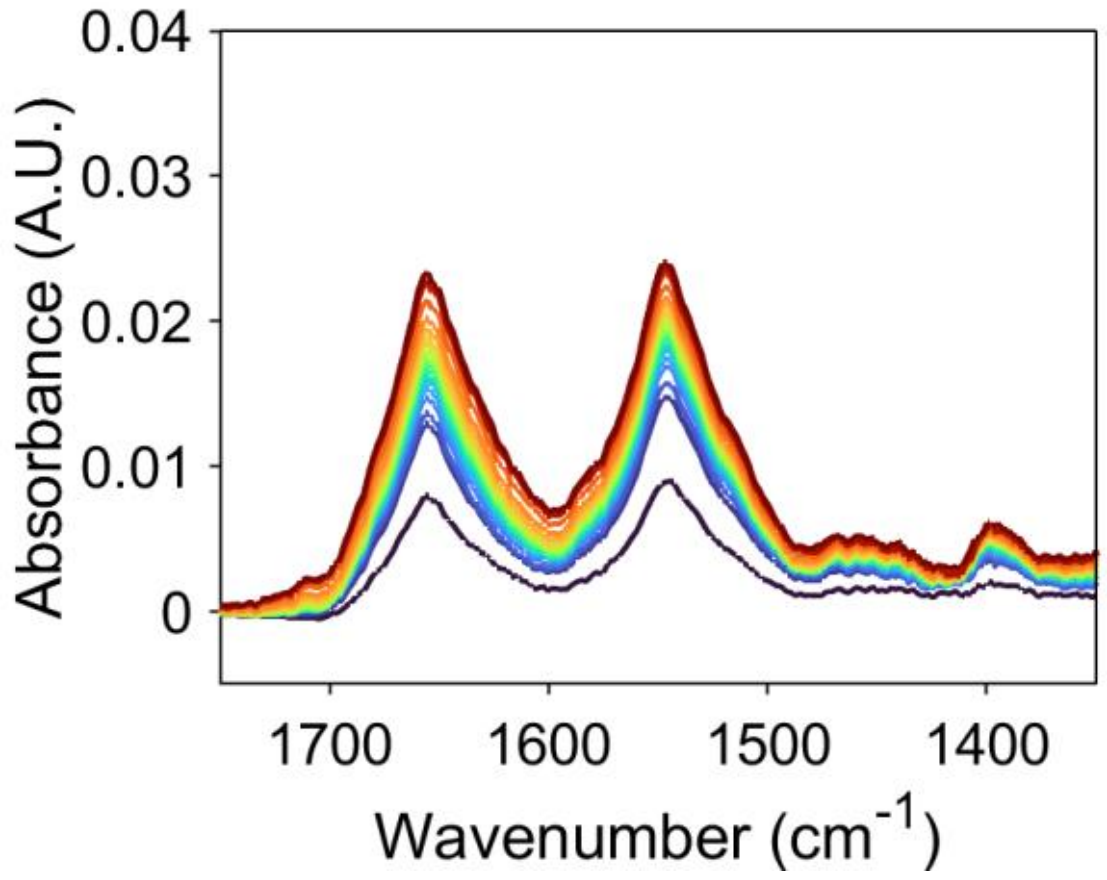

B)

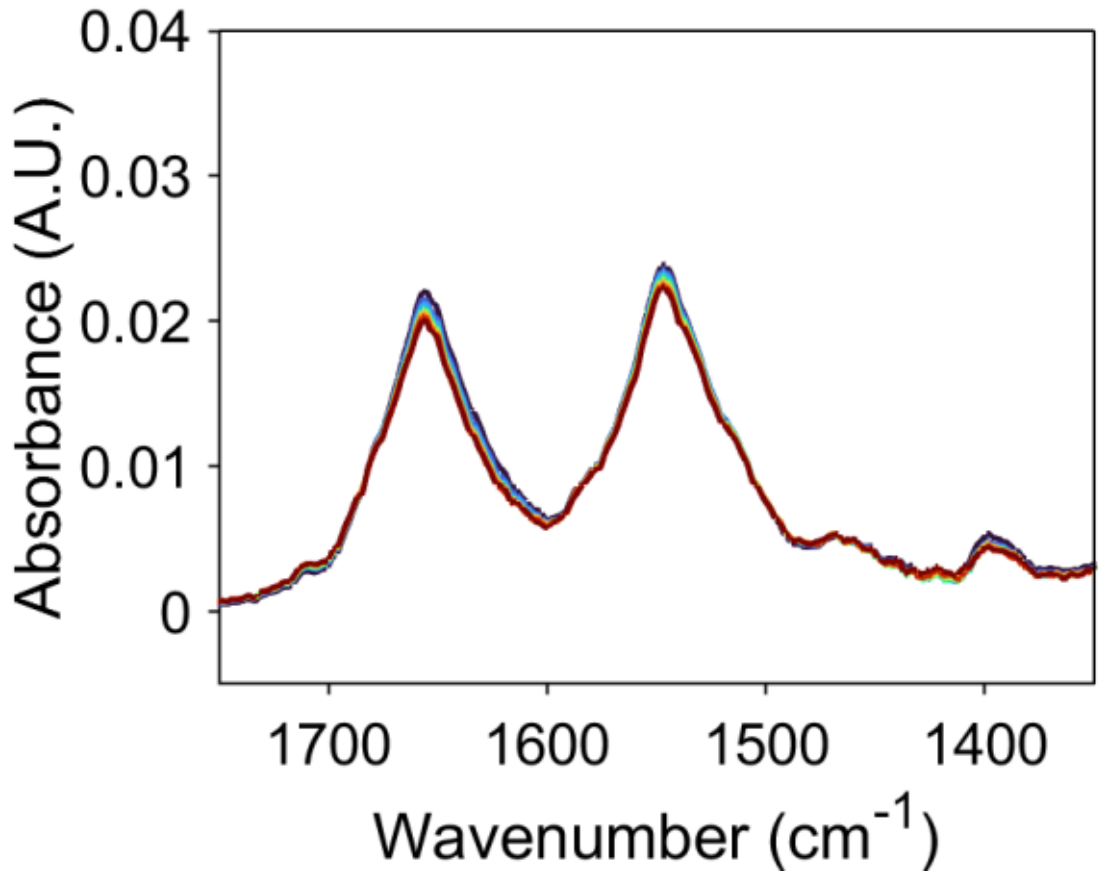

C)

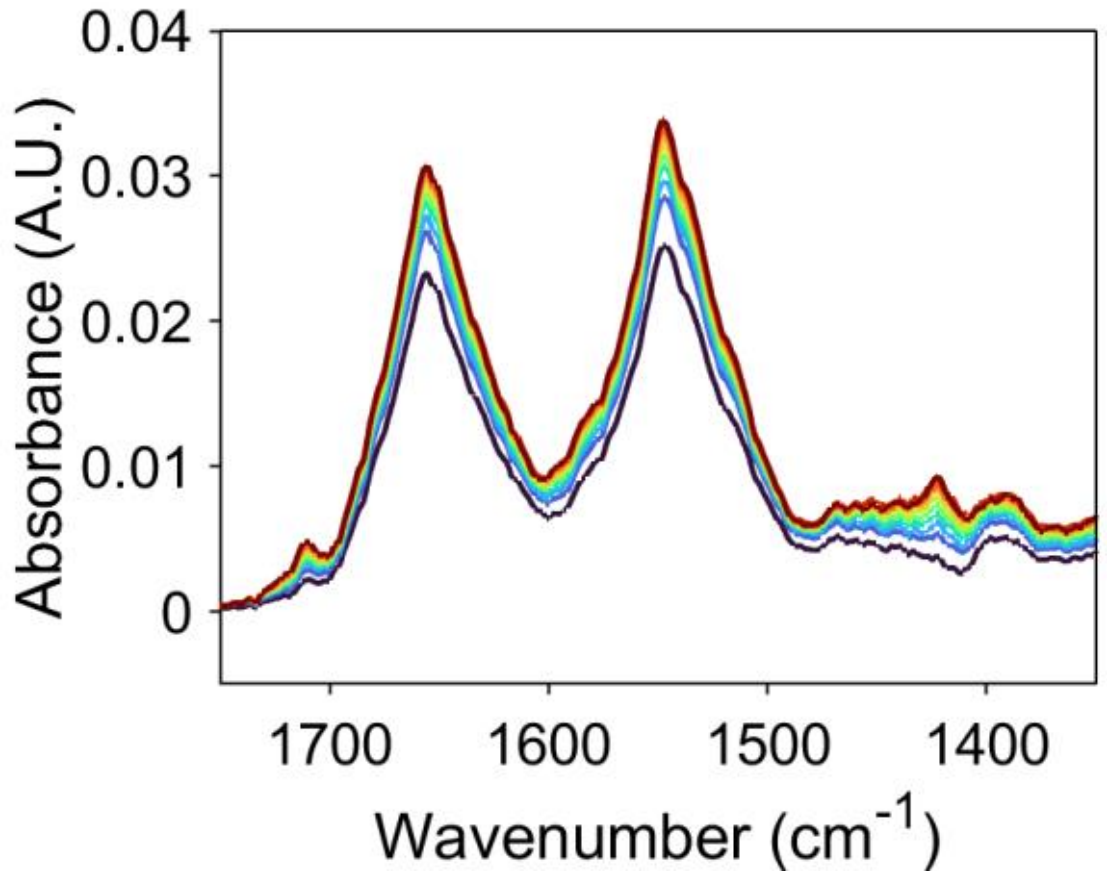

D)

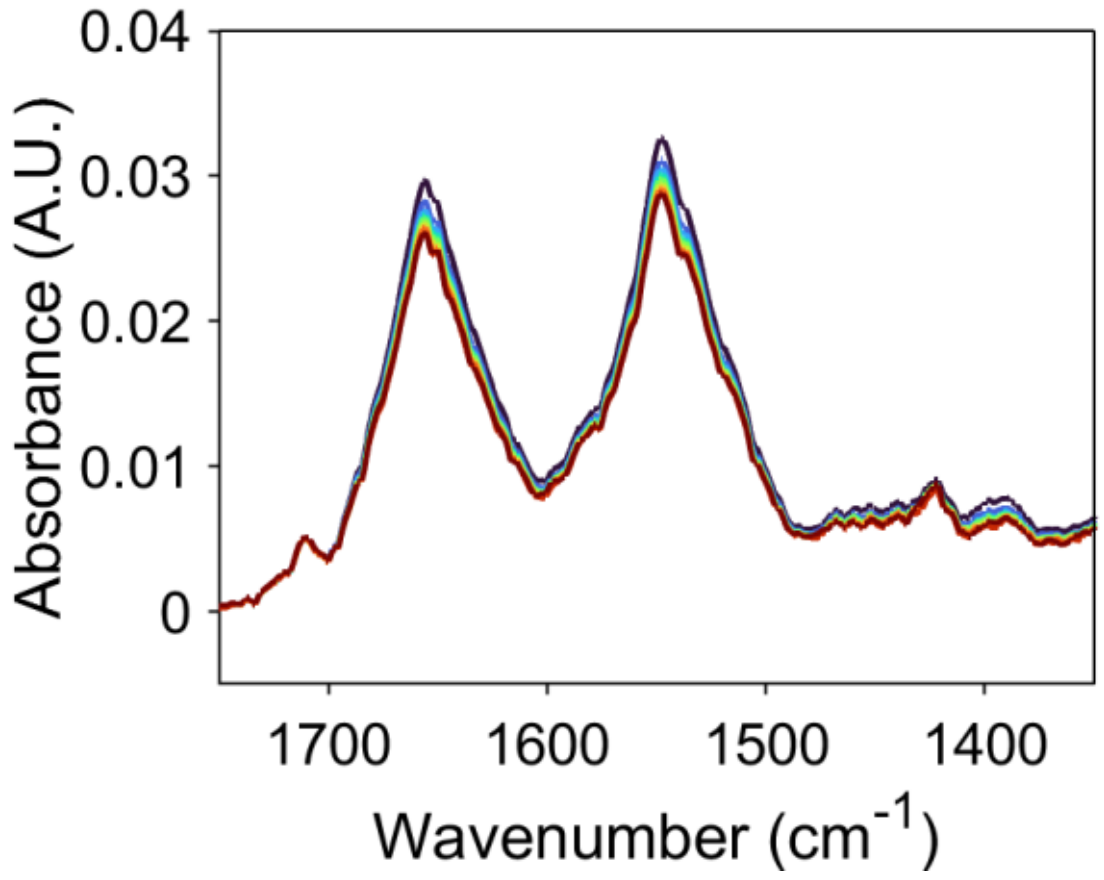

E)

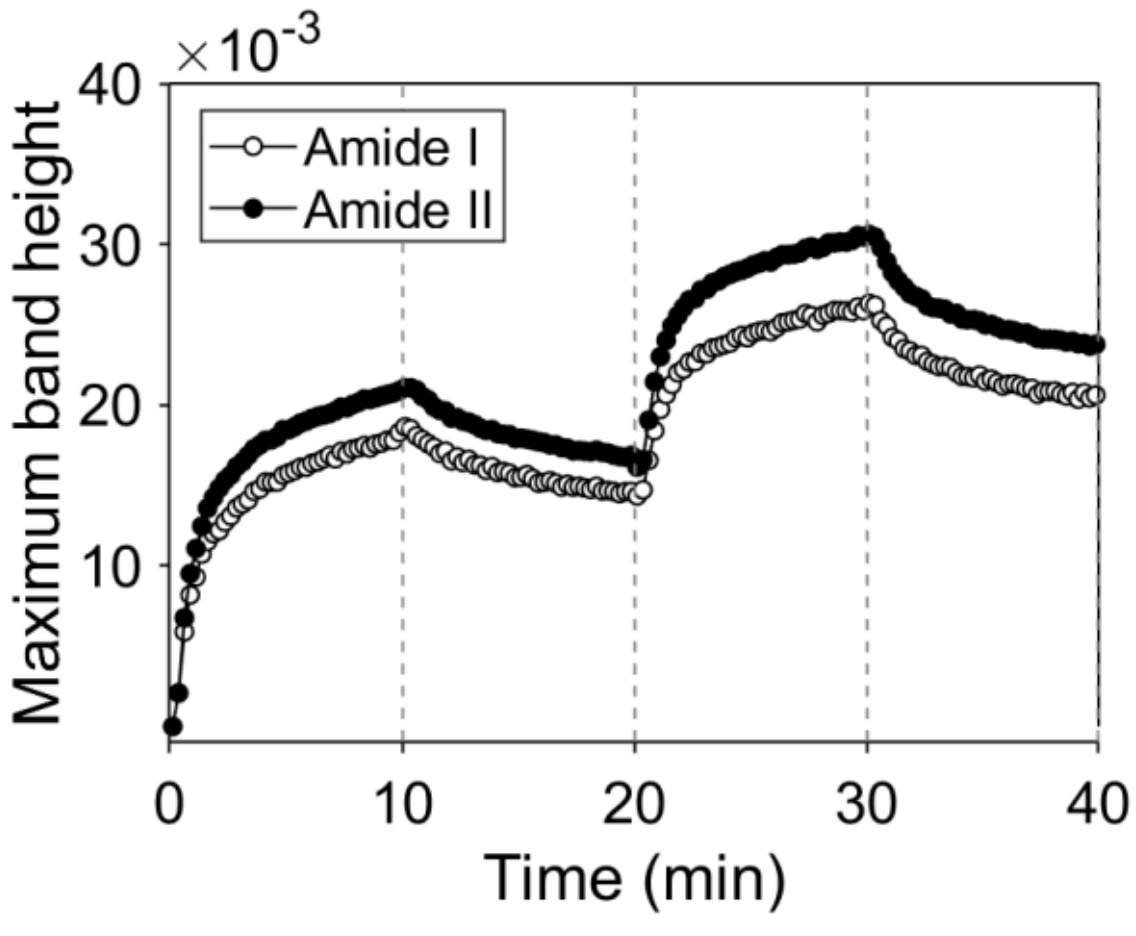

Figure S 23: **Averaged ATR-FTIR spectra of HB on blank ATR crystal (pH 6.9, not corrected).** A) 20 µM protein application. B) Washing with MilliQ. C) 80 µM protein application. D) Washing with MilliQ. E). Maximum band height of amide I (~1650 cm^{-1}, white) and amide II (~1550 cm^{-1}, black) bands plotted against time. Starting spectra are depicted in blue and transform to red. The gray dashed lines indicate solutions swaps. Background spectrum**:** blank ATR crystal flushed with MilliQ.

A)

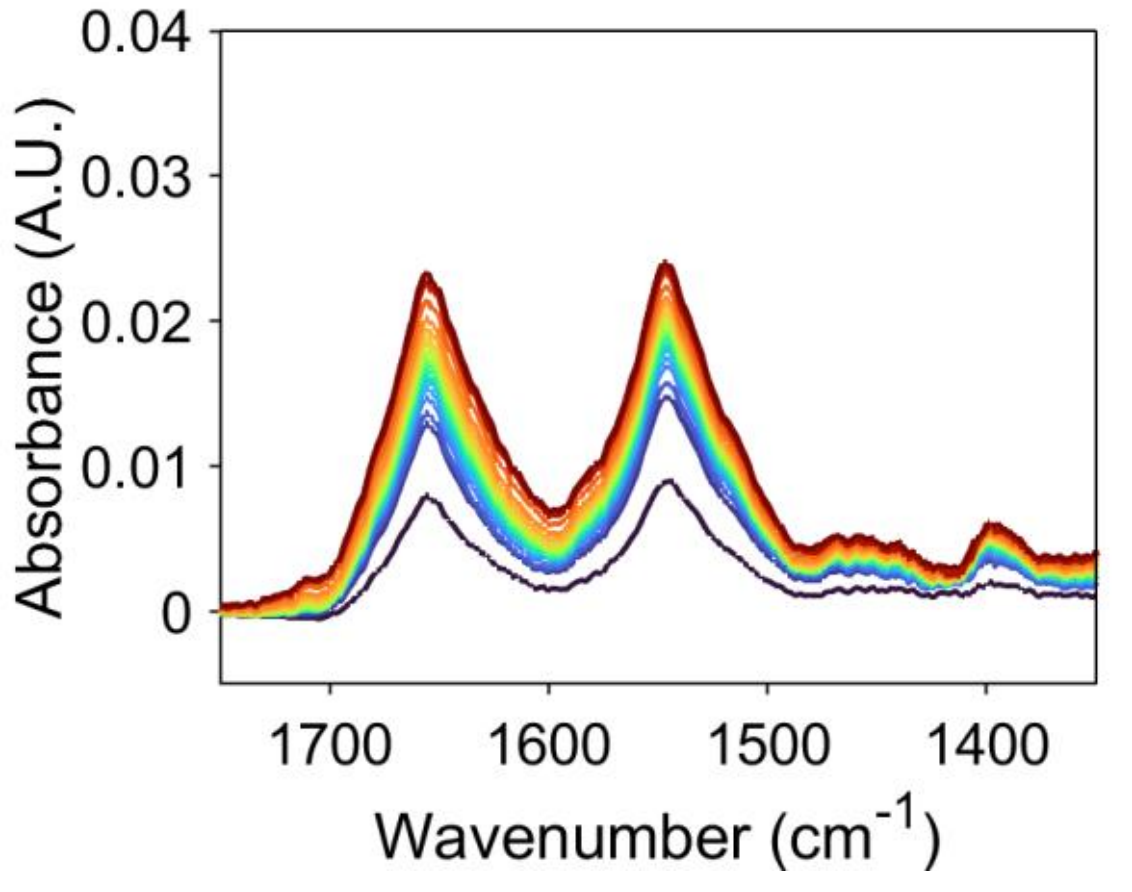

B)

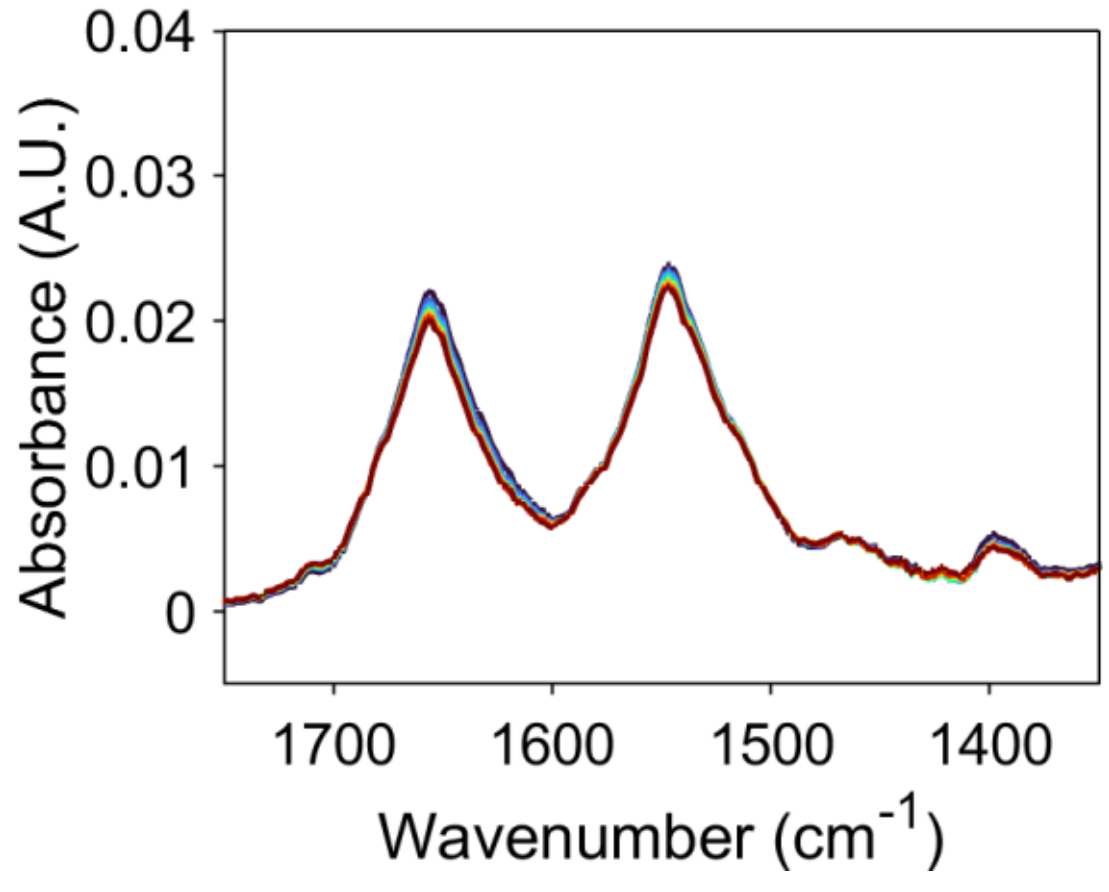

C)

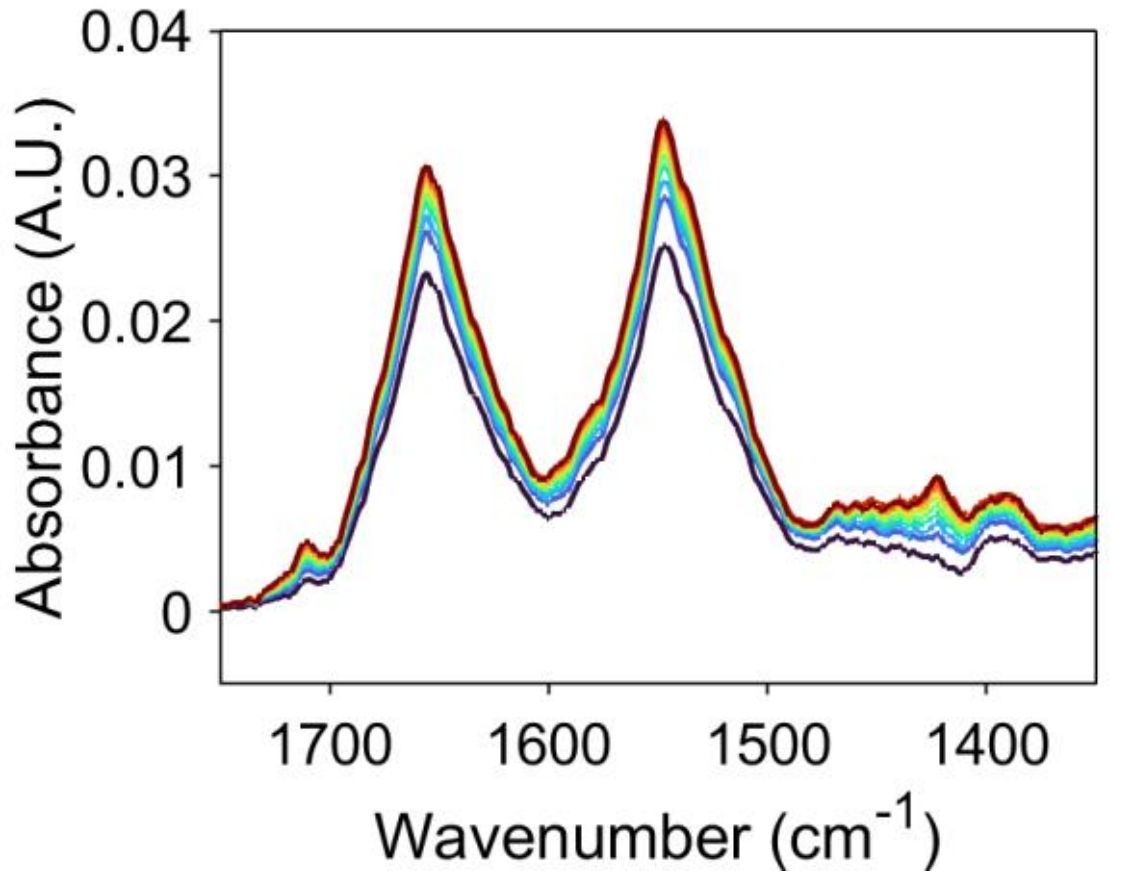

D)

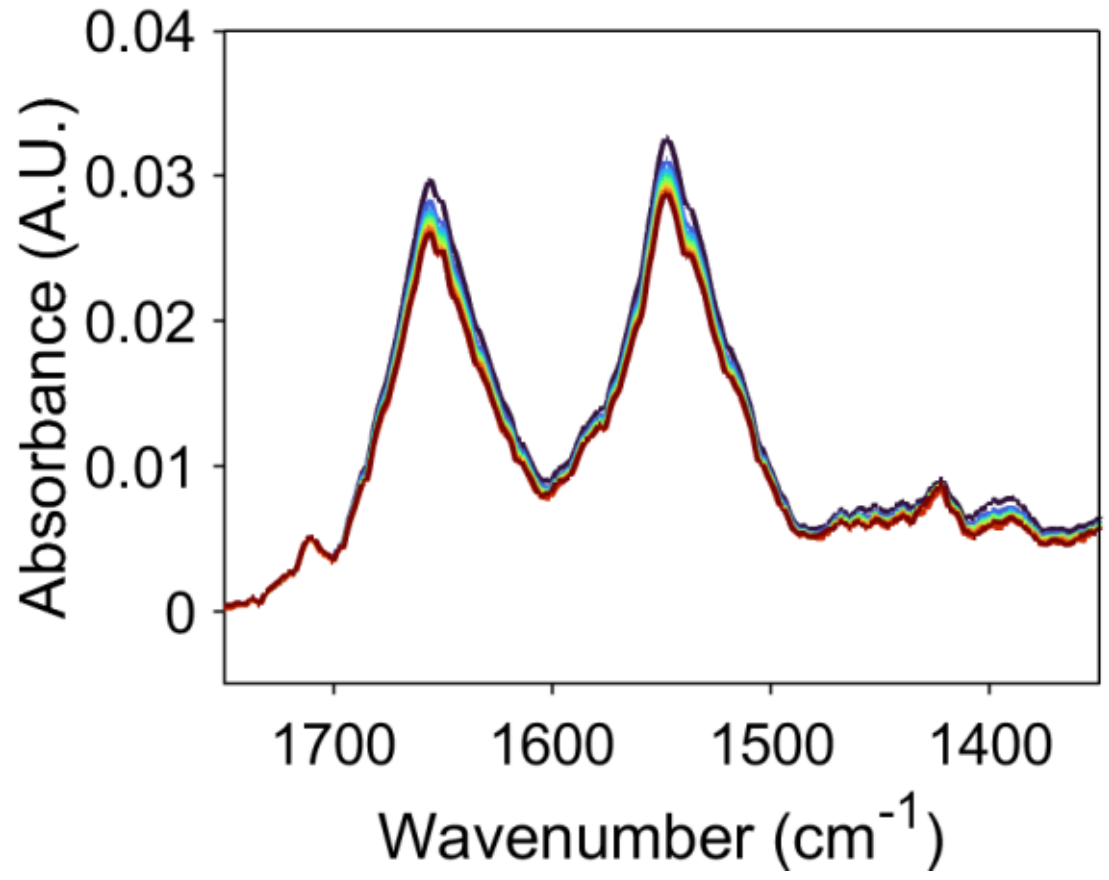

E)

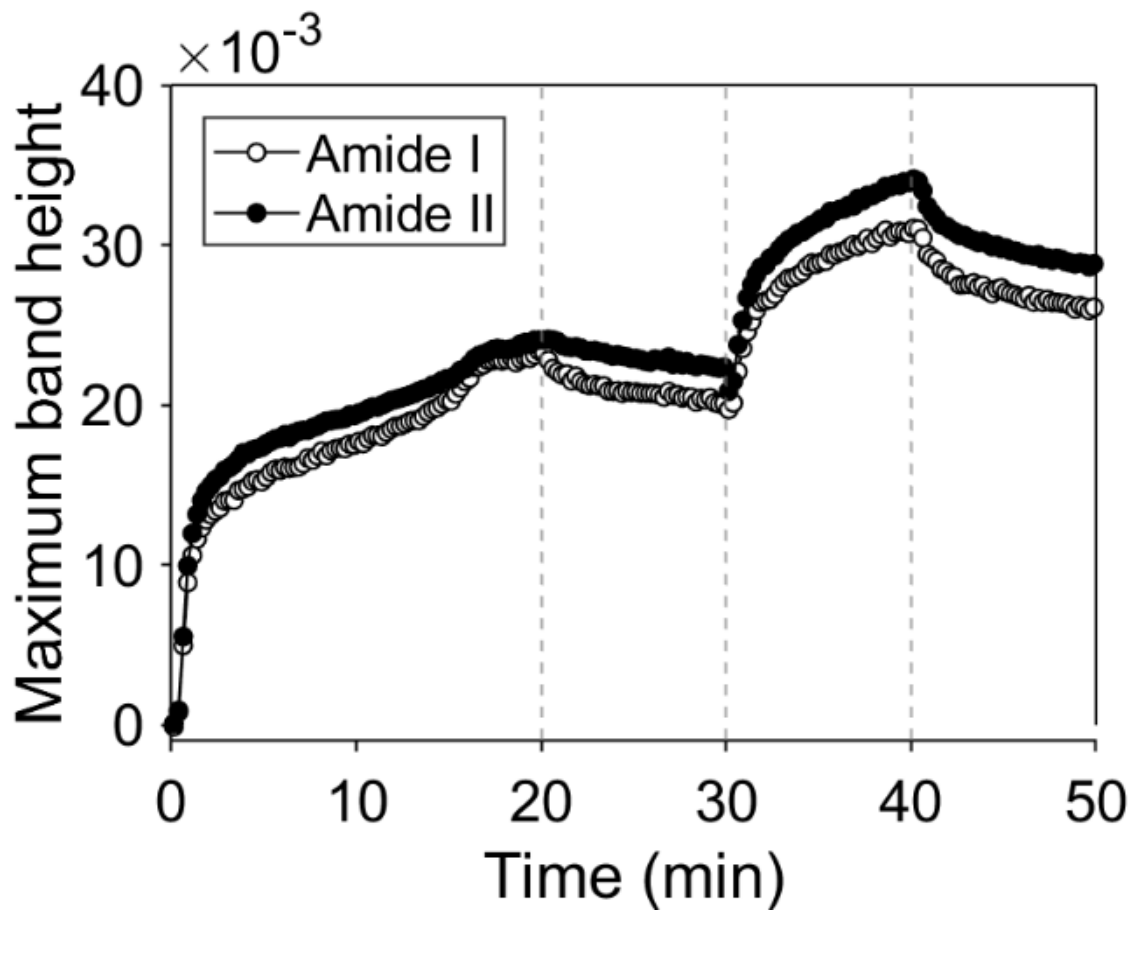

Figure S 24: **Averaged ATR-FTIR spectra of HB on NU-1000 coated ATR crystal (pH 6.9, not corrected).** A) 20 µM protein application. B) Washing with MilliQ. C) 80 µM protein application. D) Washing with MilliQ. E). Maximum band height of amide I (~1650 cm^{-1}, white) and amide II (~1550 cm^{-1}, black) bands plotted against time. Starting spectra are depicted in blue and transform to red. The gray dashed lines indicate solutions swaps. Background spectrum**:** NU-1000 coated ATR crystal flushed with MilliQ.

A)

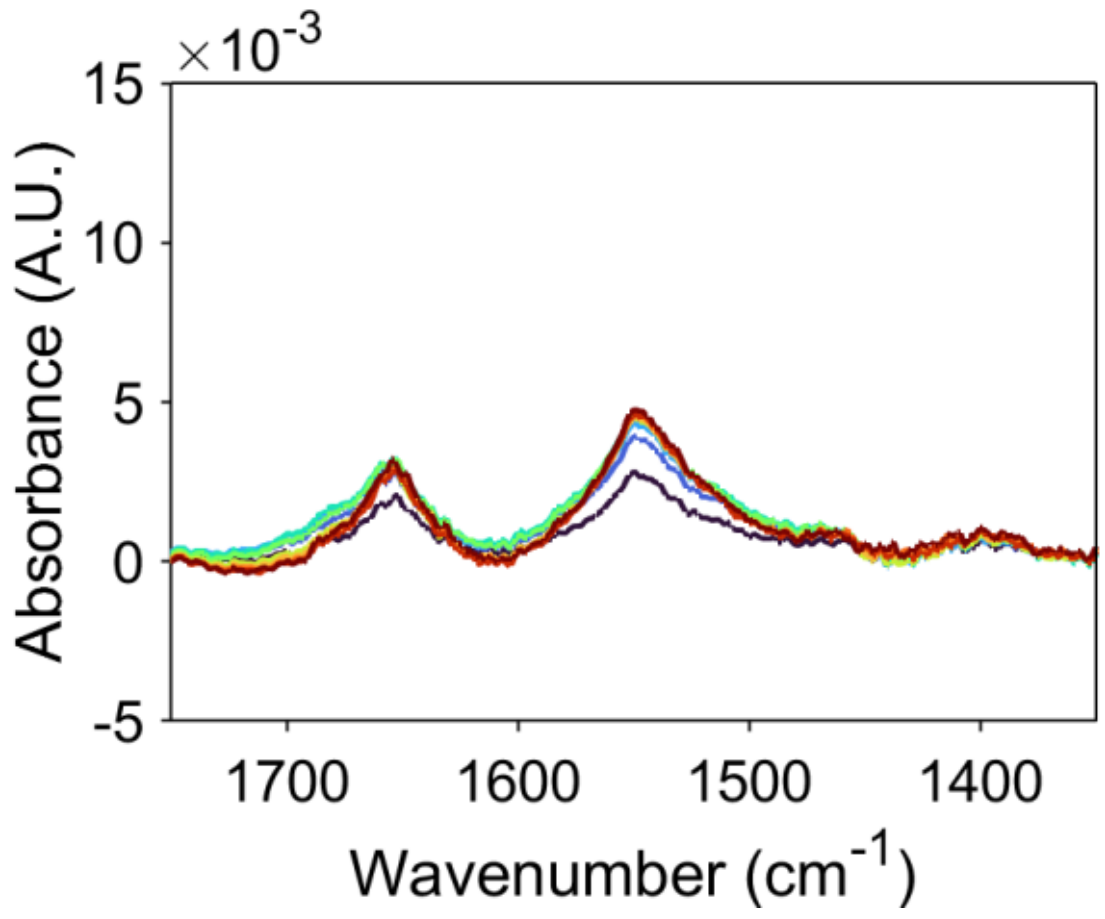

B)

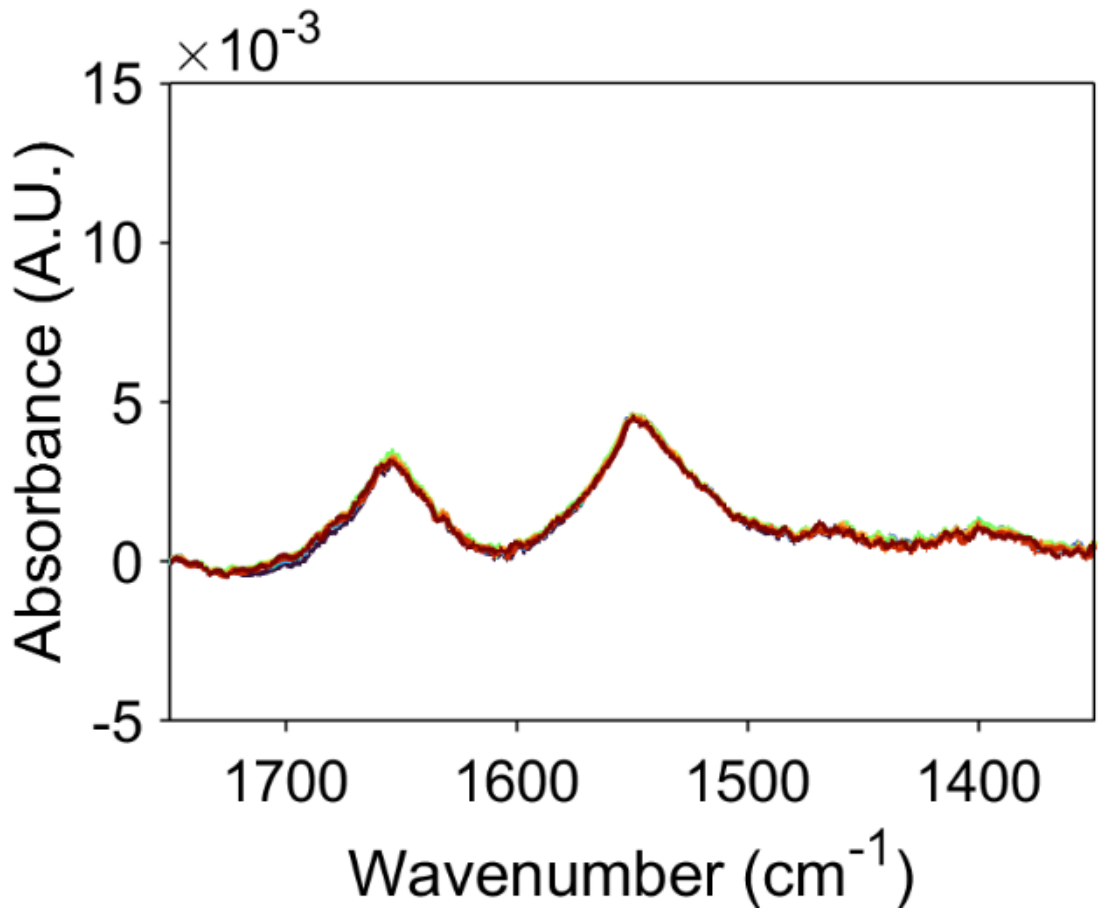

C)

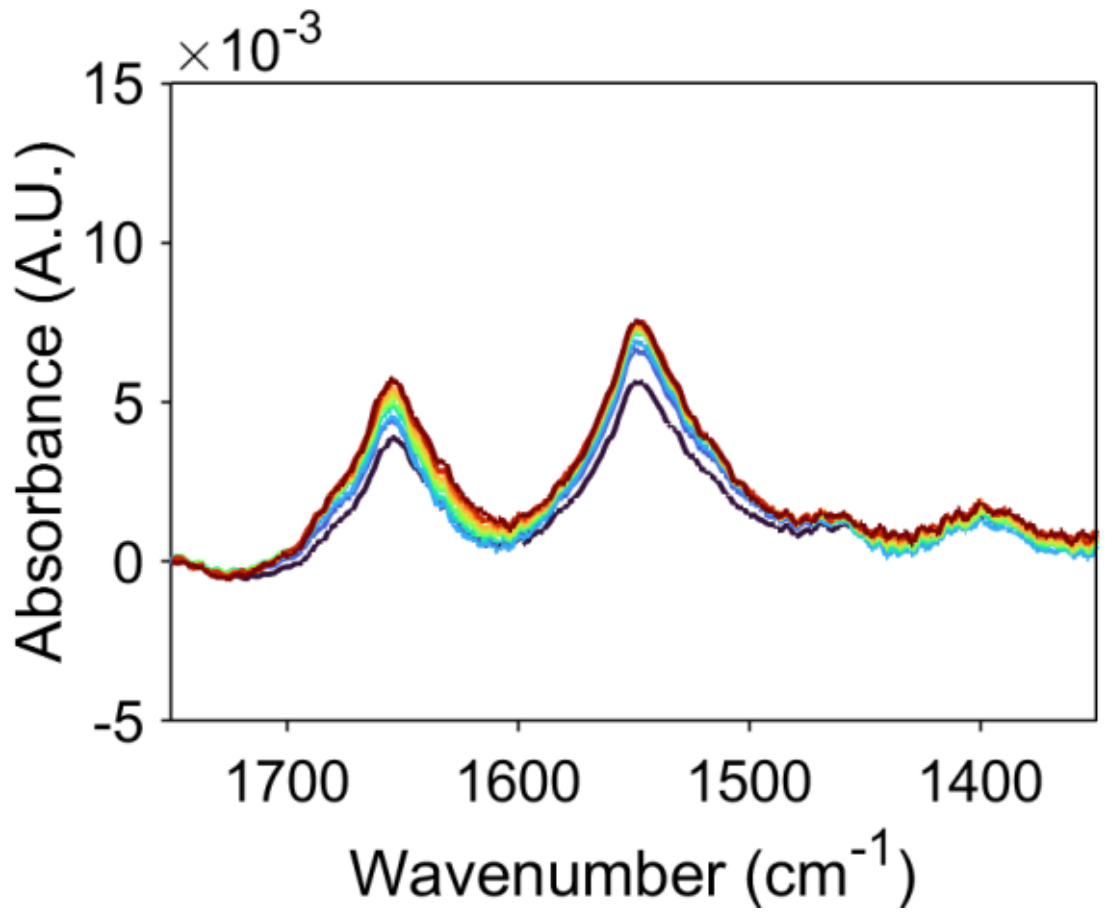

D)

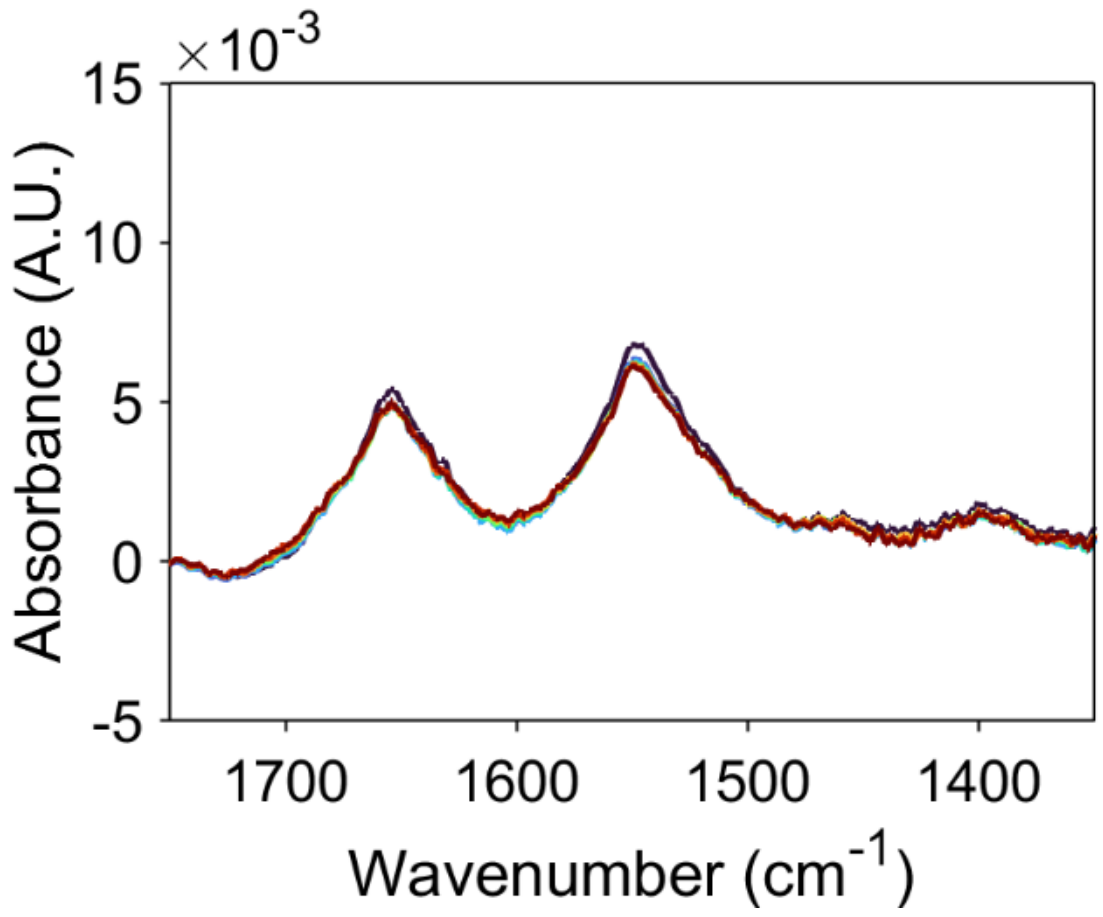

E)

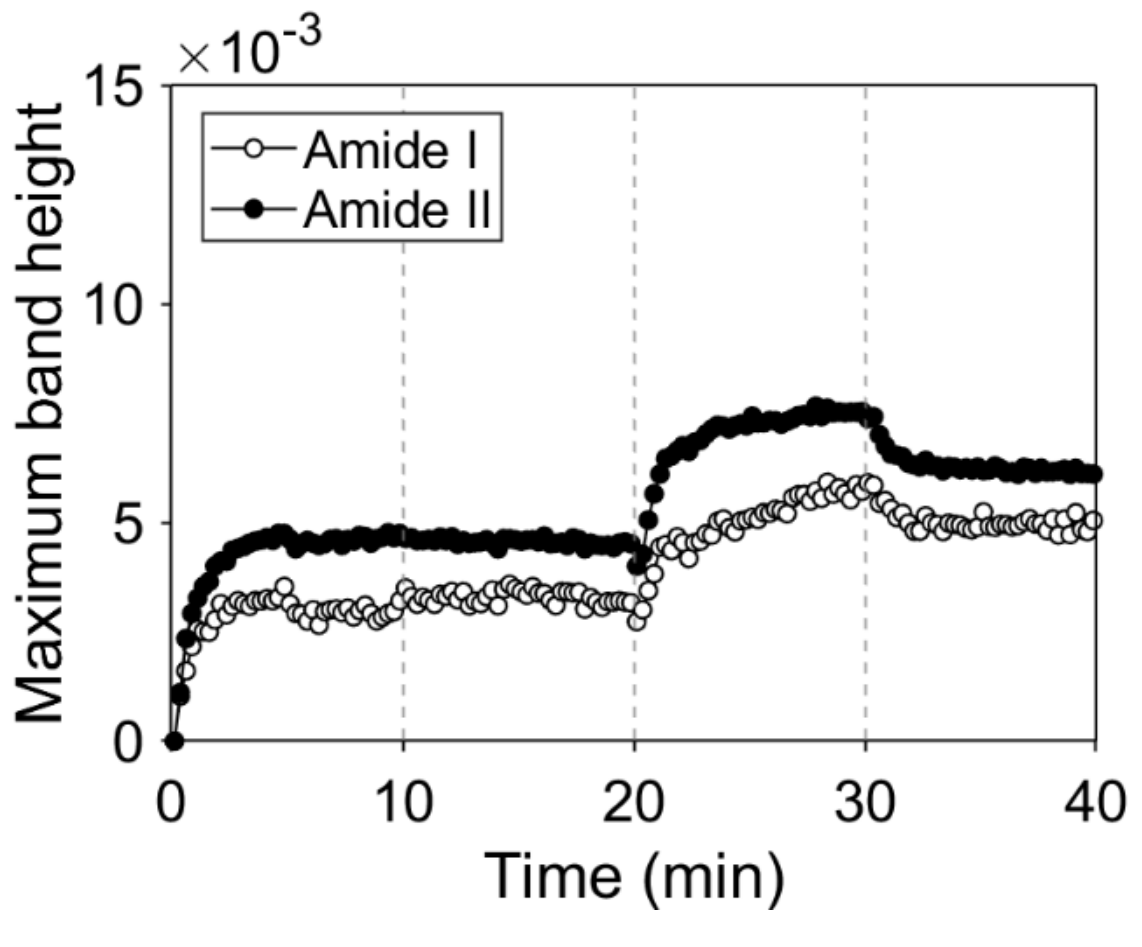

Figure S 25: **Averaged ATR-FTIR spectra of MB on blank ATR crystal (corrected to pH 7).** A) 20 µM protein application. B) Washing with MilliQ. C) 80 µM protein application. D) Washing with MilliQ. E). Maximum band height of amide I (~1650 cm^{-1}, white) and amide II (~1550 cm^{-1}, black) bands plotted against time. Starting spectra are depicted in blue and transform to red. The gray dashed lines indicate solutions swaps. Background spectrum**:** blank ATR crystal flushed with MilliQ.

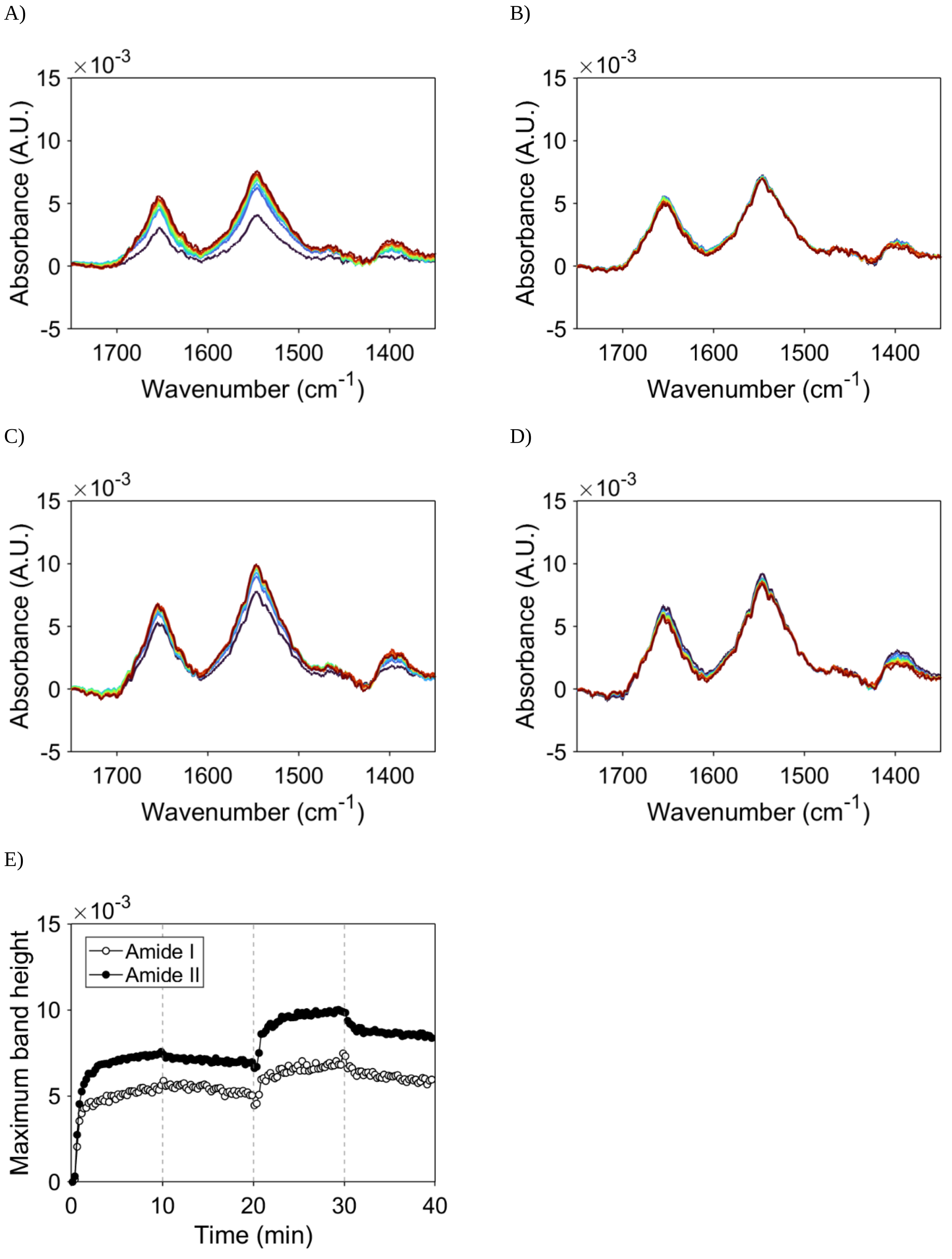

Figure S 26: **Averaged ATR-FTIR spectra of MB on NU-1000 coated ATR crystal (corrected to pH 7).** A) 20 µM protein application. B) Washing with MilliQ. C) 80 µM protein application. D) Washing with MilliQ. E). Maximum band height of amide I (~1650 cm^{-1}, white) and amide II (~1550 cm^{-1}, black) bands plotted against time. Starting spectra are depicted in blue and transform to red. The gray dashed lines indicate solutions swaps. Background spectrum**:** NU-1000 coated ATR crystal flushed with MilliQ.

A)

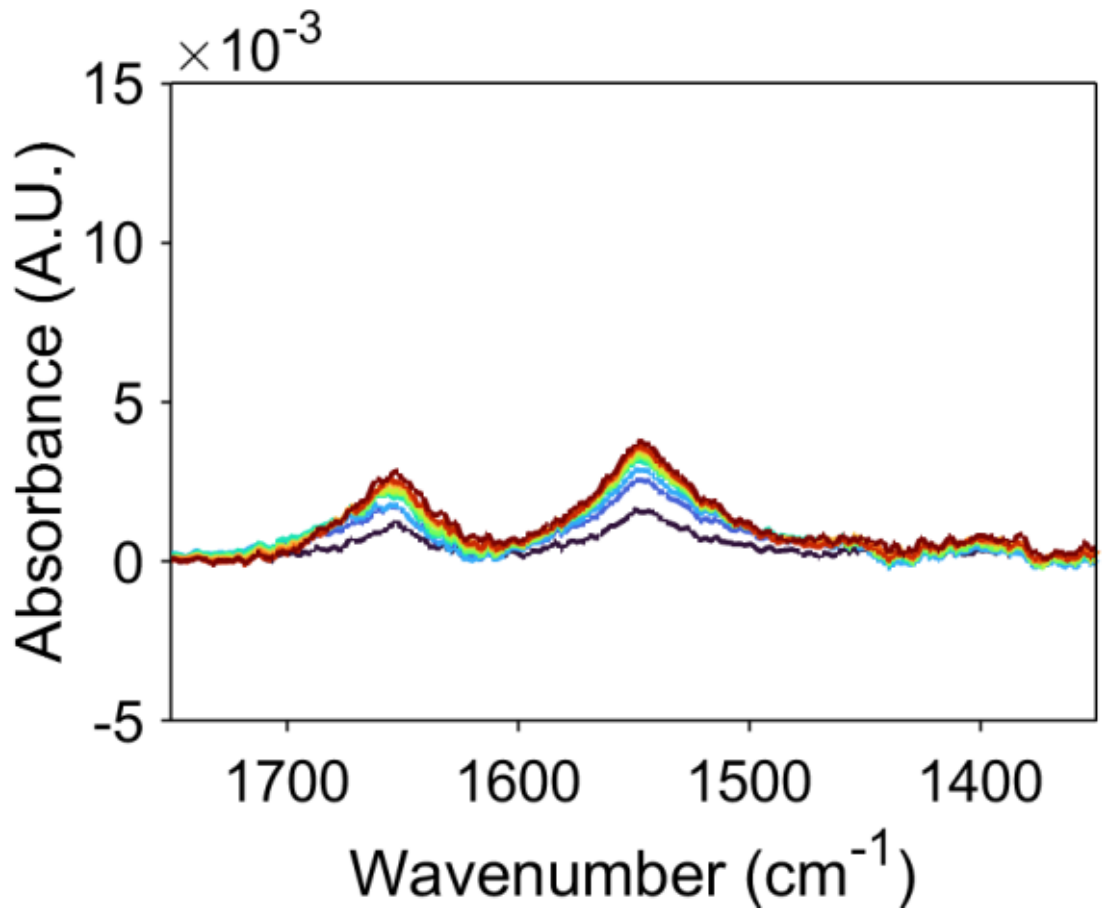

B)

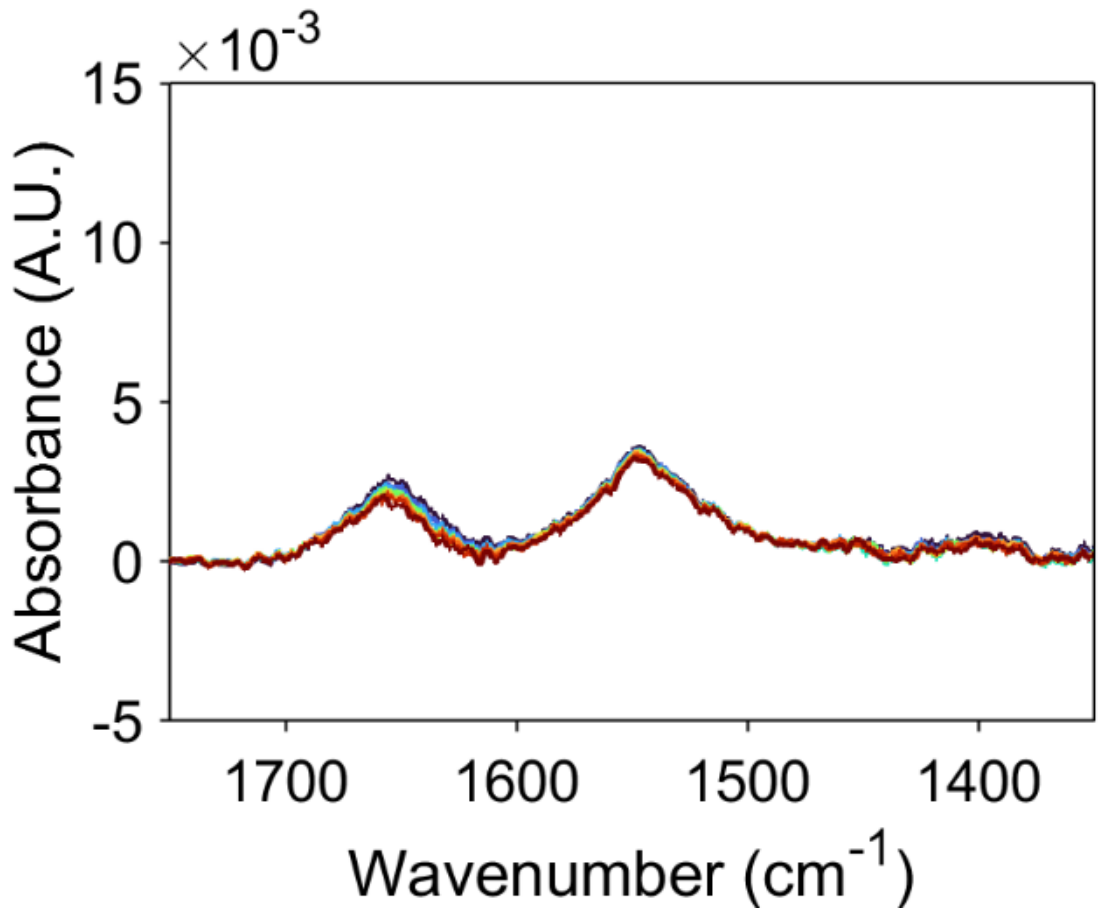

C)

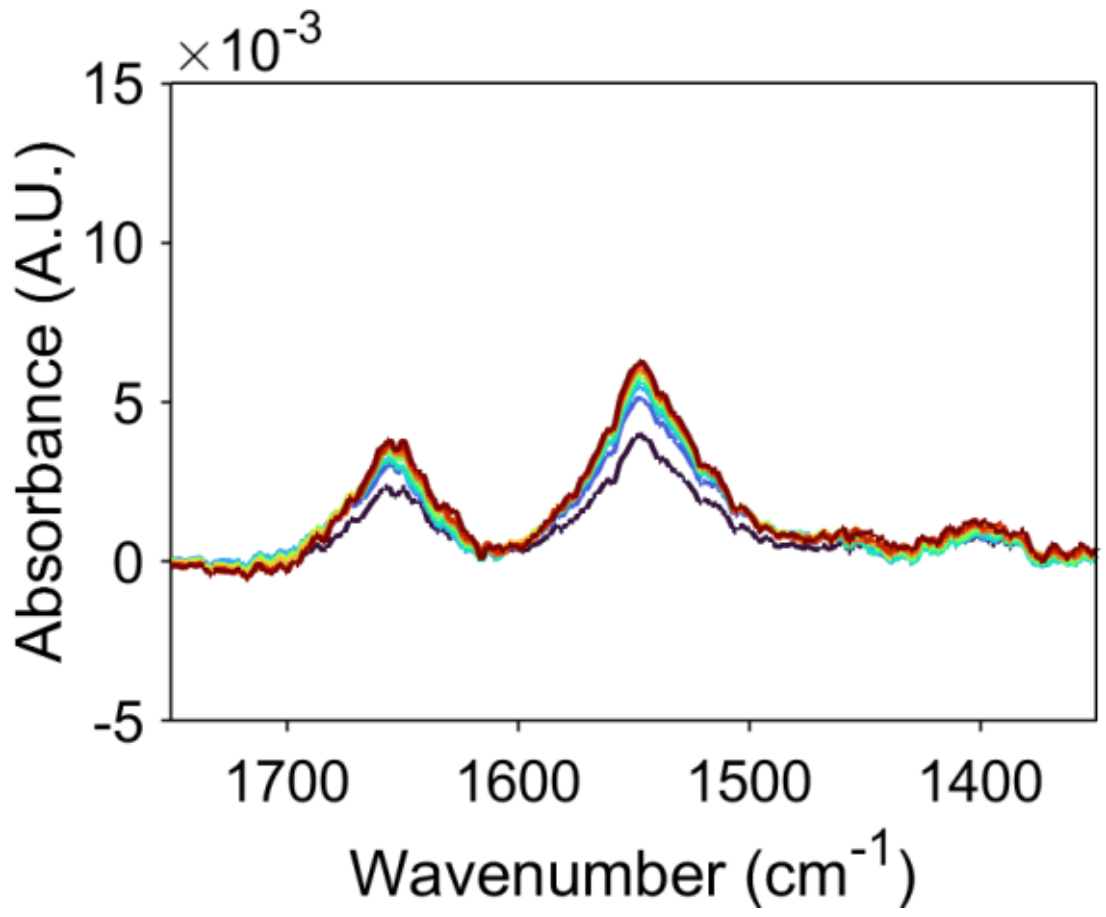

D)

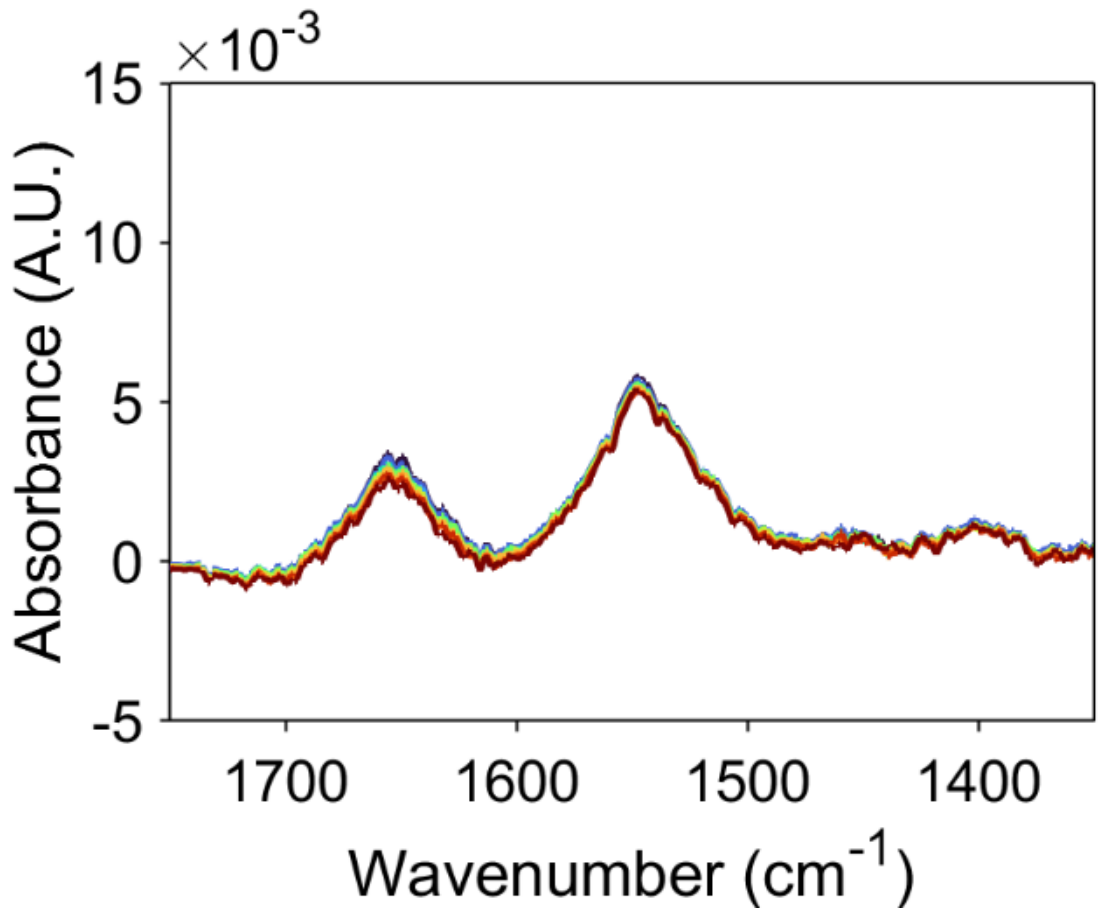

E)

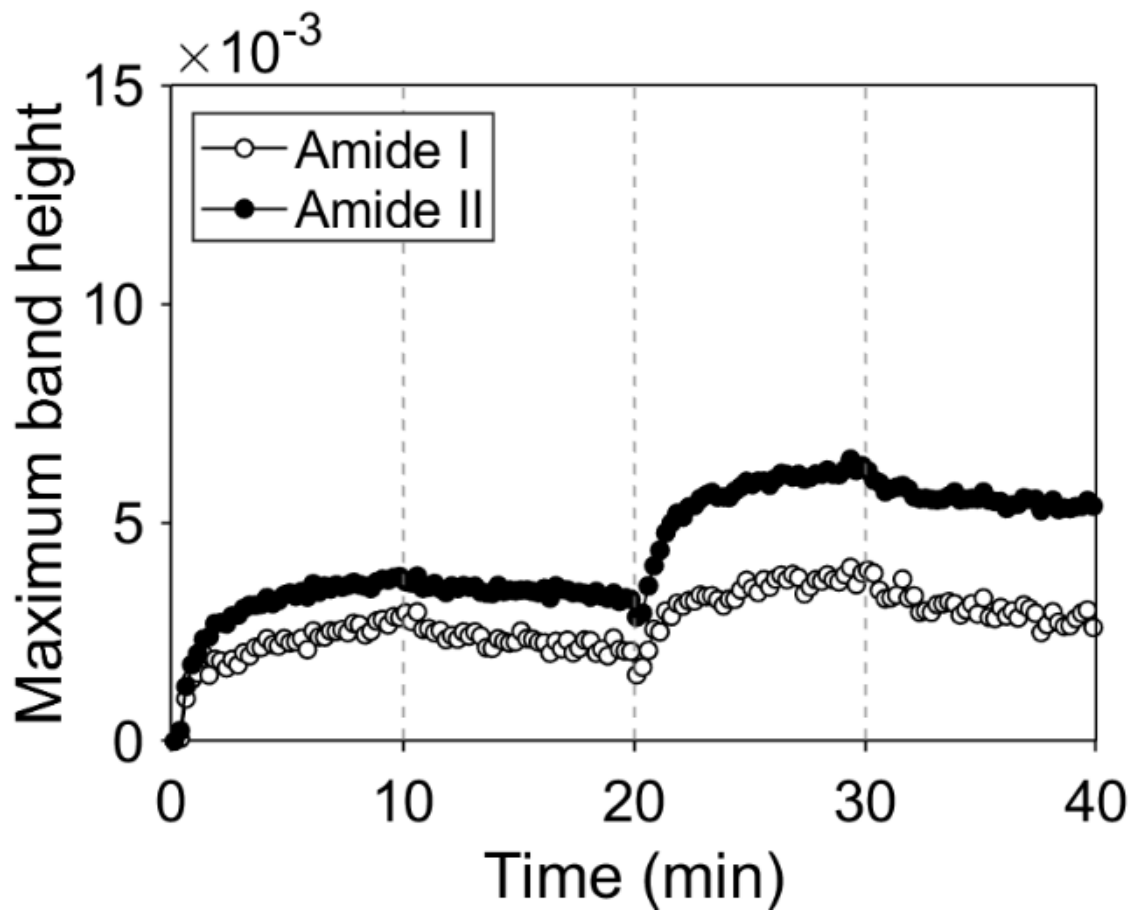

Figure S 27: **Averaged ATR-FTIR spectra of MB on blank ATR crystal (corrected to pH 5).** A) 20 µM protein application. B) Washing with MilliQ. C) 80 µM protein application. D) Washing with MilliQ. E). Maximum band height of amide I (~1650 cm^{-1}, white) and amide II (~1550 cm^{-1}, black) bands plotted against time. Starting spectra are depicted in blue and transform to red. The gray dashed lines indicate solutions swaps. Background spectrum**:** blank ATR crystal flushed with MilliQ.

A)

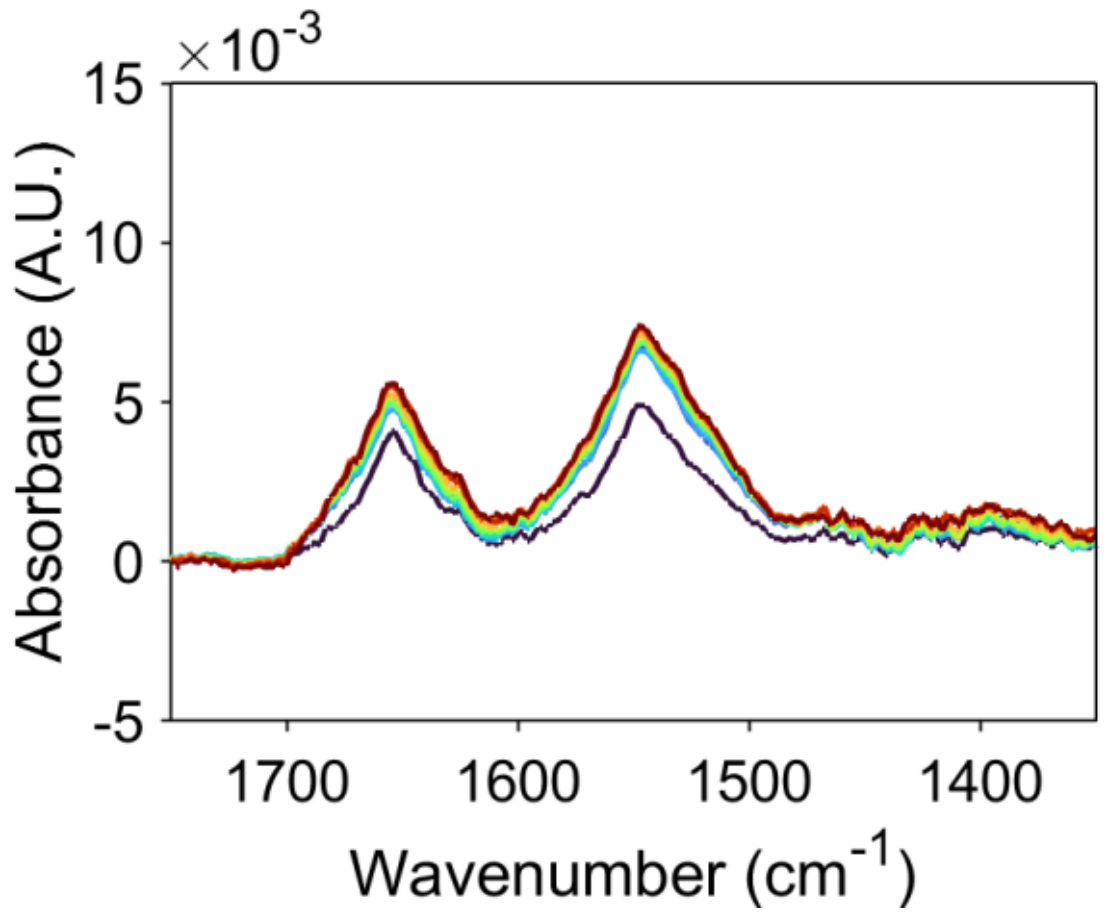

B)

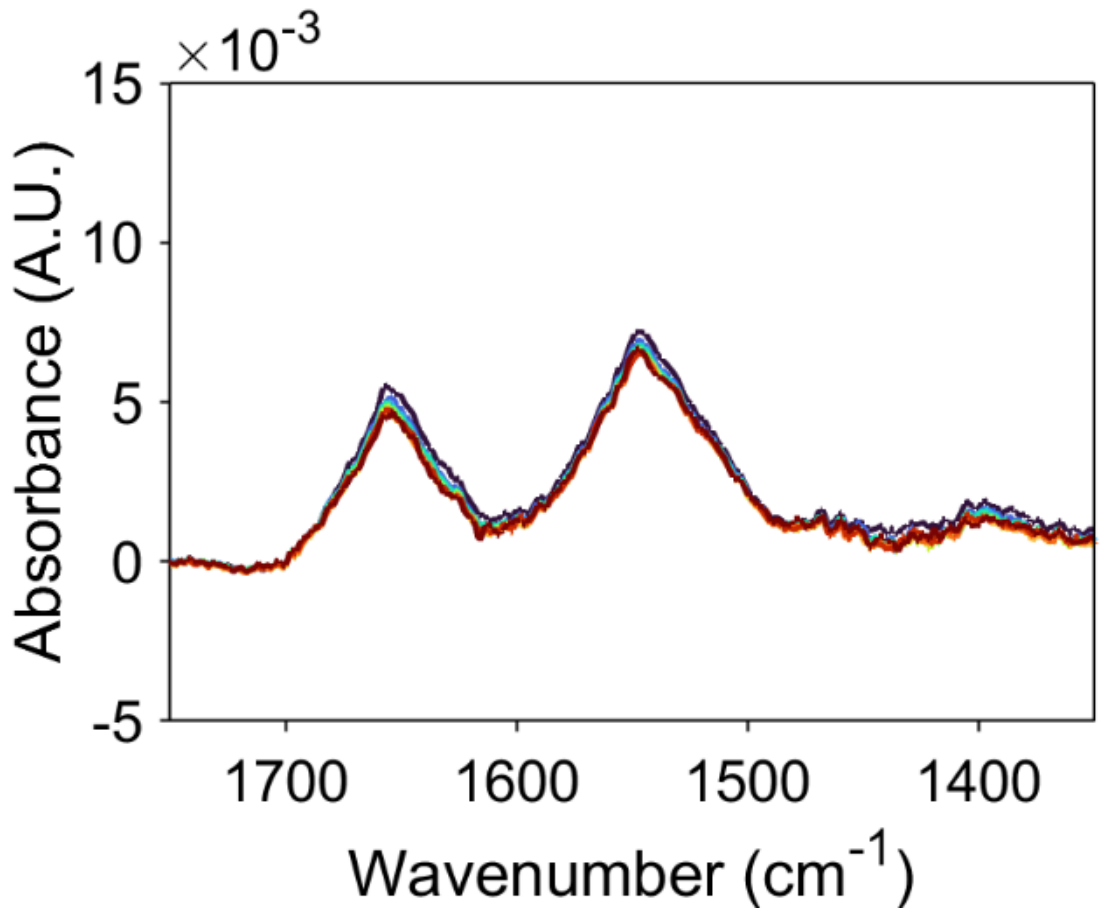

C)

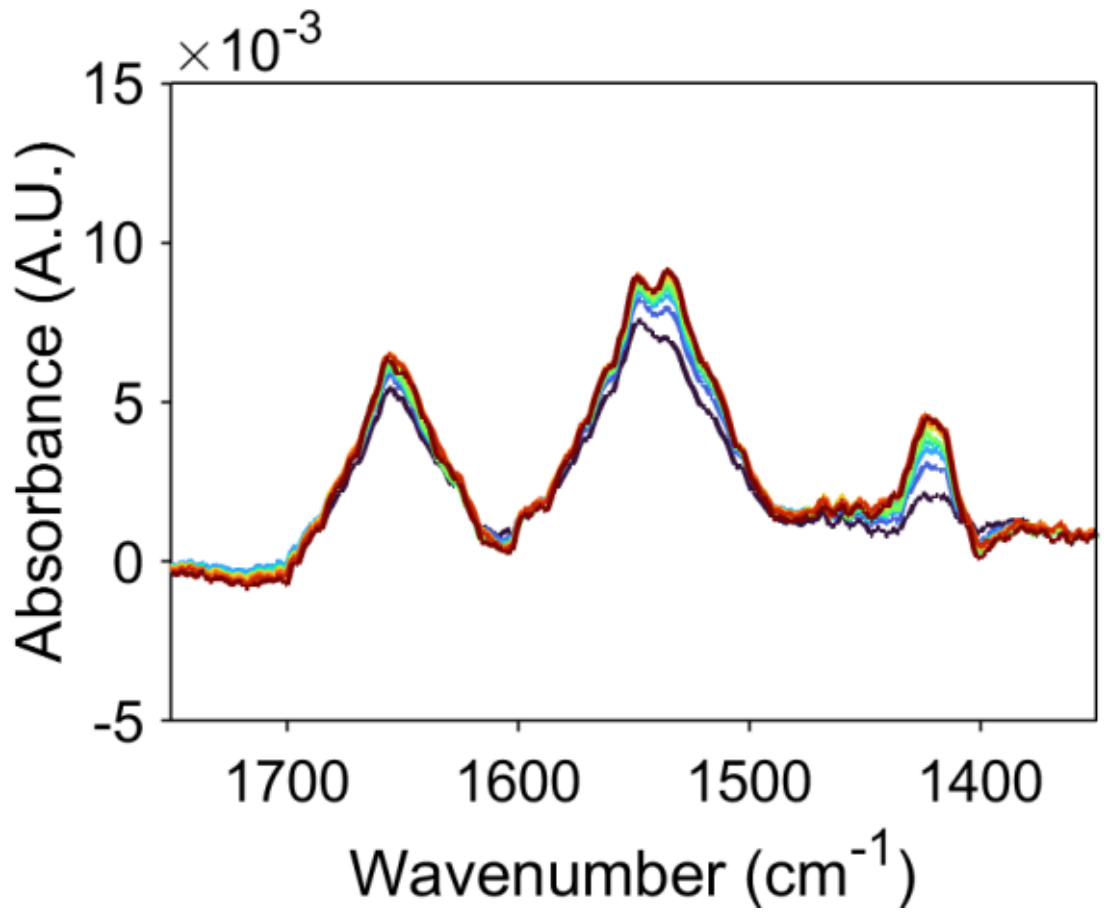

D)

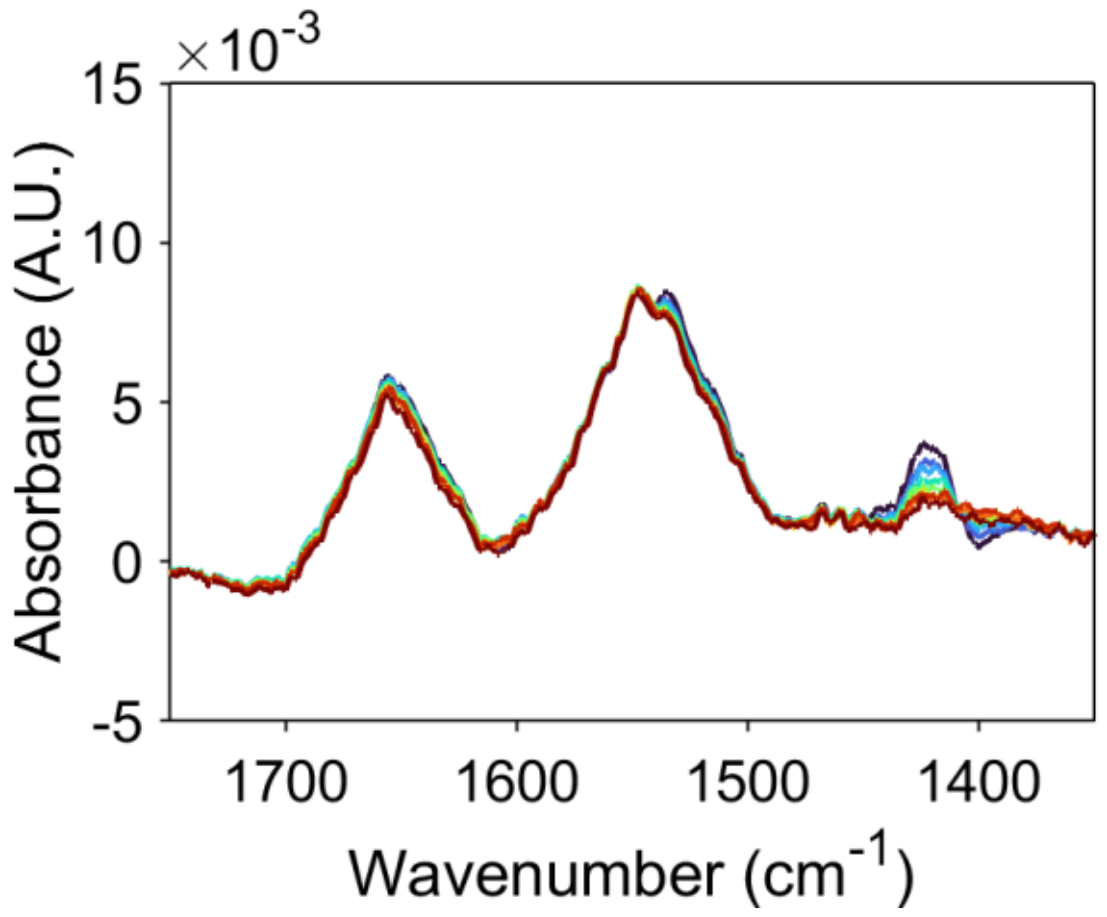

E)

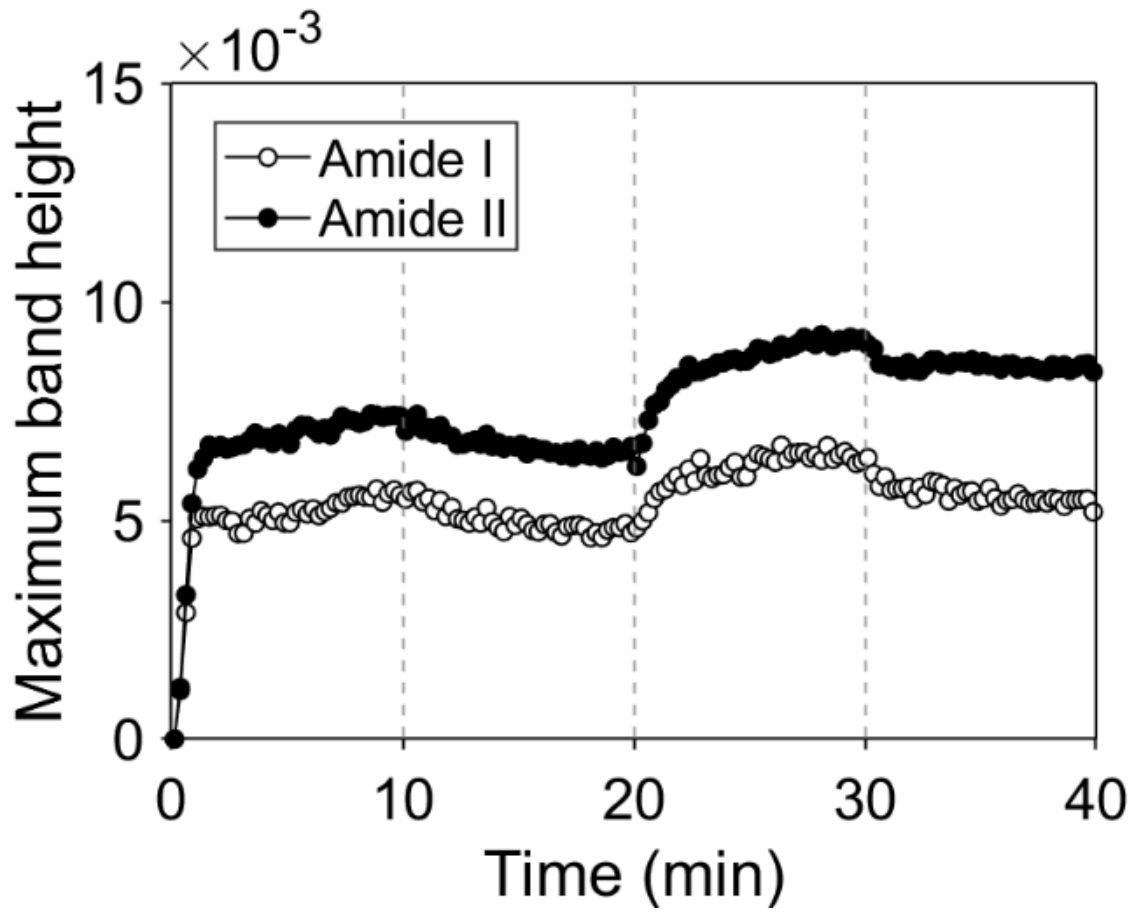

Figure S 28: **Averaged ATR-FTIR spectra of MB on NU-1000 coated ATR crystal (corrected to pH 5).** A) 20 µM protein application. B) Washing with MilliQ. C) 80 µM protein application. D) Washing with MilliQ. E). Maximum band height of amide I (~1650 cm^{-1}, white) and amide II (~1550 cm^{-1}, black) bands plotted against time. Starting spectra are depicted in blue and transform to red. The gray dashed lines indicate solutions swaps. Background spectrum**:** NU-1000 coated ATR crystal flushed with MilliQ.

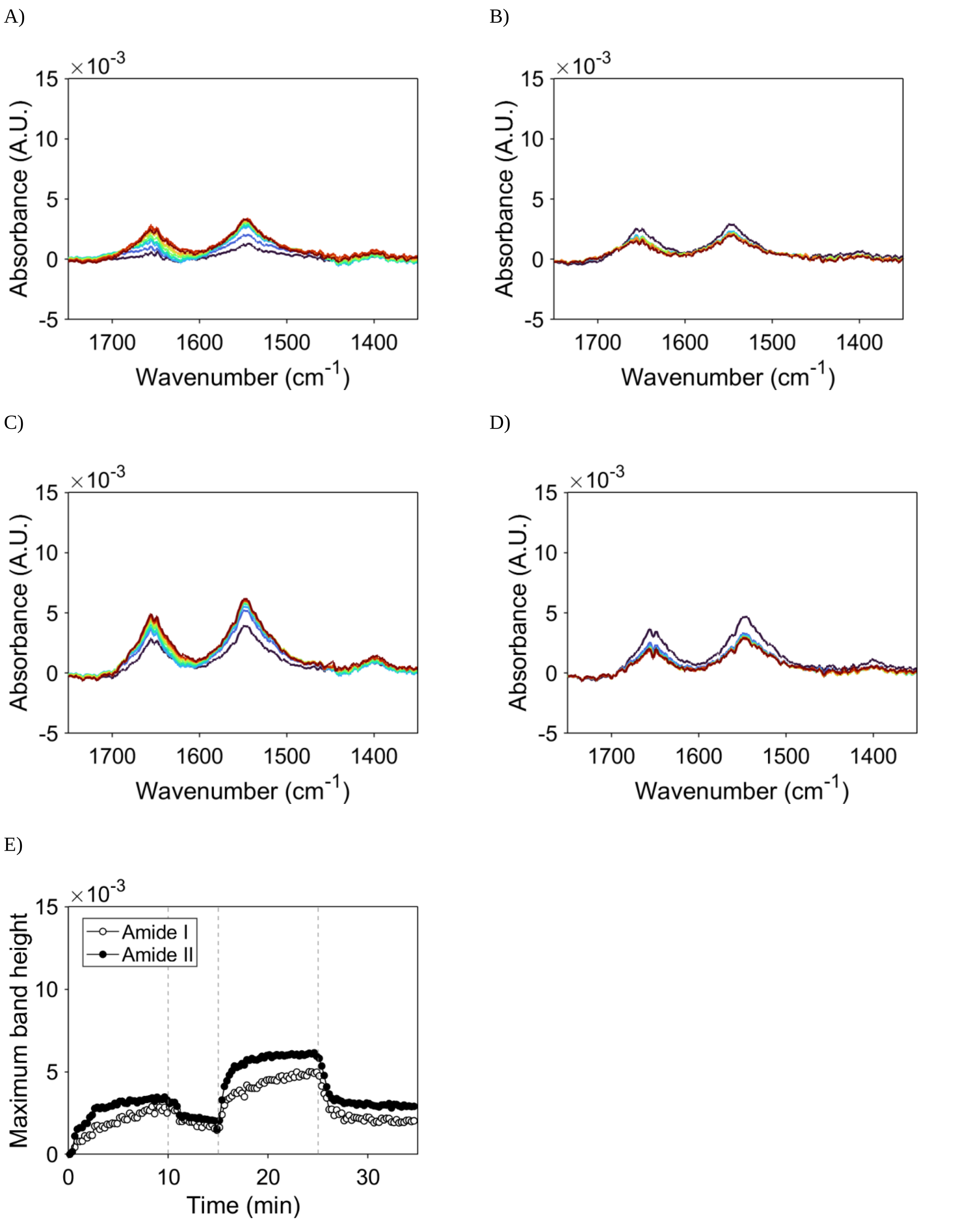

Figure S 29: **Averaged ATR-FTIR spectra of MB on blank ATR crystal (pH 7.4, not corrected).** A) 20 µM protein application. B) Washing with MilliQ. C) 80 µM protein application. D) Washing with MilliQ. E). Maximum band height of amide I (~1650 cm^{-1}, white) and amide II (~1550 cm^{-1}, black) bands plotted against time. Starting spectra are depicted in blue and transform to red. The gray dashed lines indicate solutions swaps. Background spectrum**:** blank ATR crystal flushed with MilliQ.

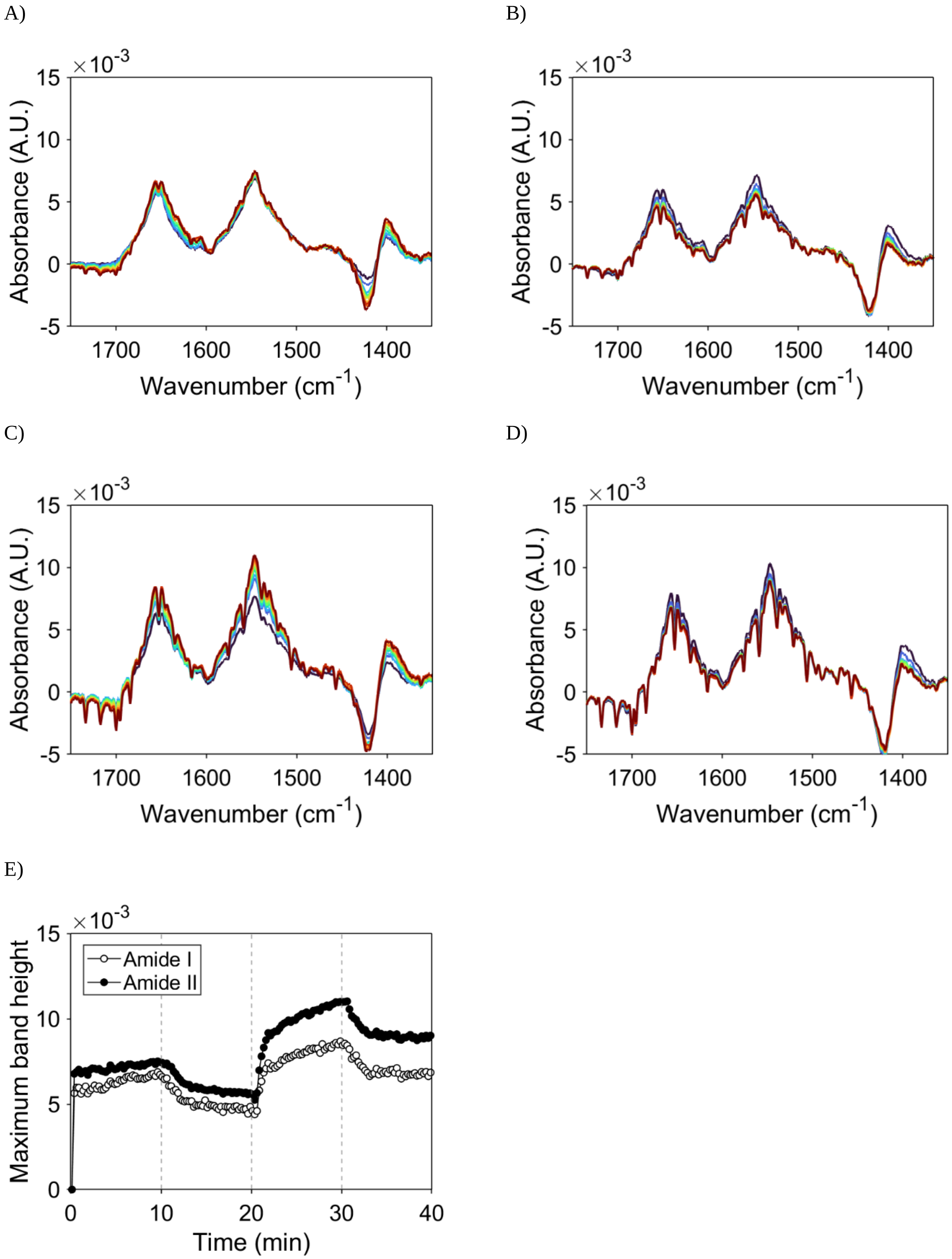

Figure S 30: **Averaged ATR-FTIR spectra of MB on NU-1000 coated ATR crystal (pH 7.4, not corrected).** A) 20 µM protein application. B) Washing with MilliQ. C) 80 µM protein application. D) Washing with MilliQ. E). Maximum band height of amide I (~1650 cm^{-1}, white) and amide II (~1550 cm^{-1}, black) bands plotted against time. Starting spectra are depicted in blue and transform to red. The gray dashed lines indicate solutions swaps. Background spectrum**:** NU-1000 coated ATR crystal flushed with MilliQ.

A)

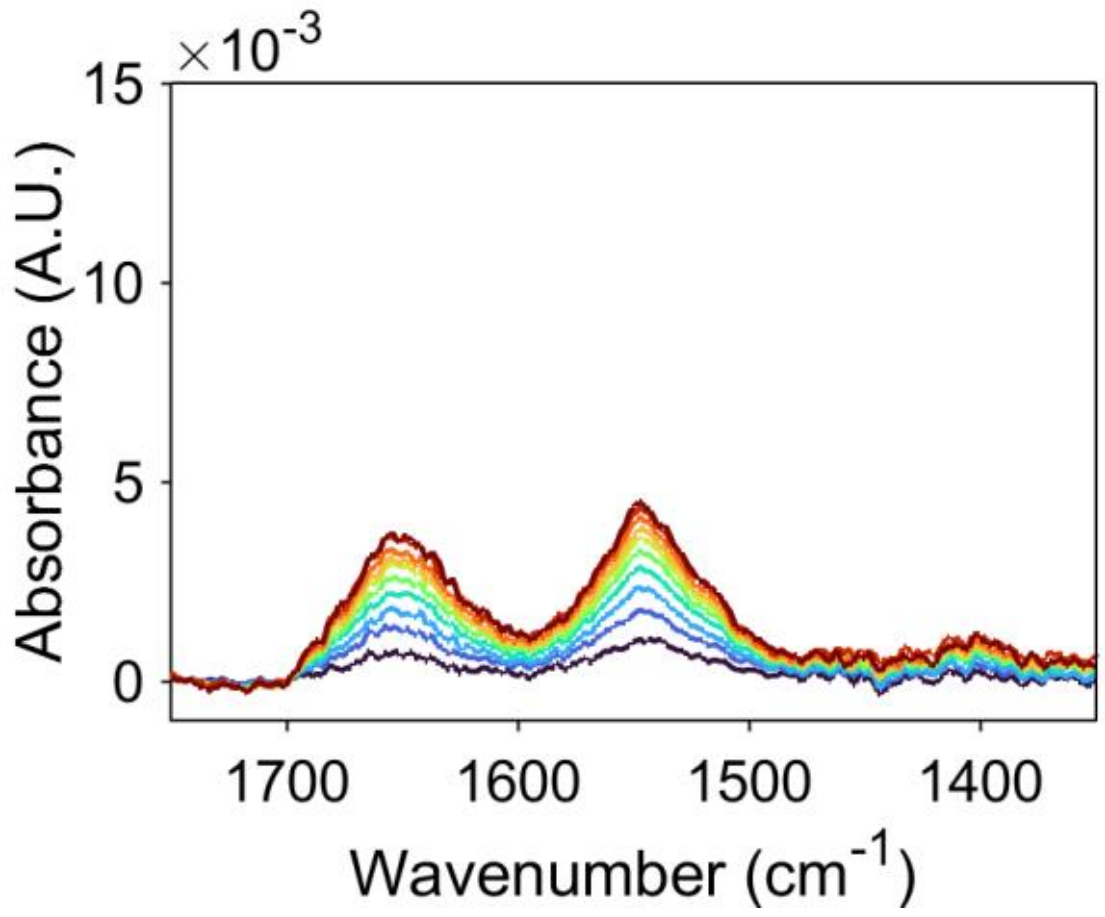

B)

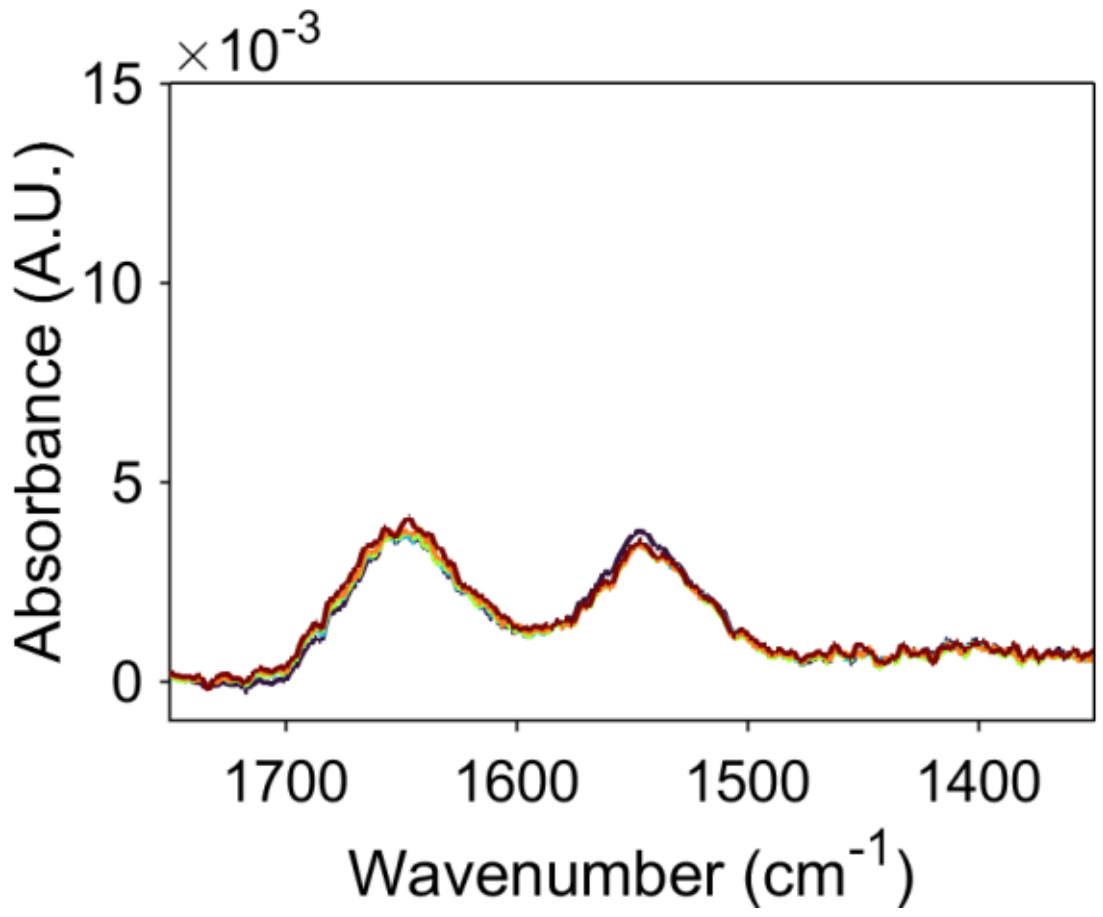

C)

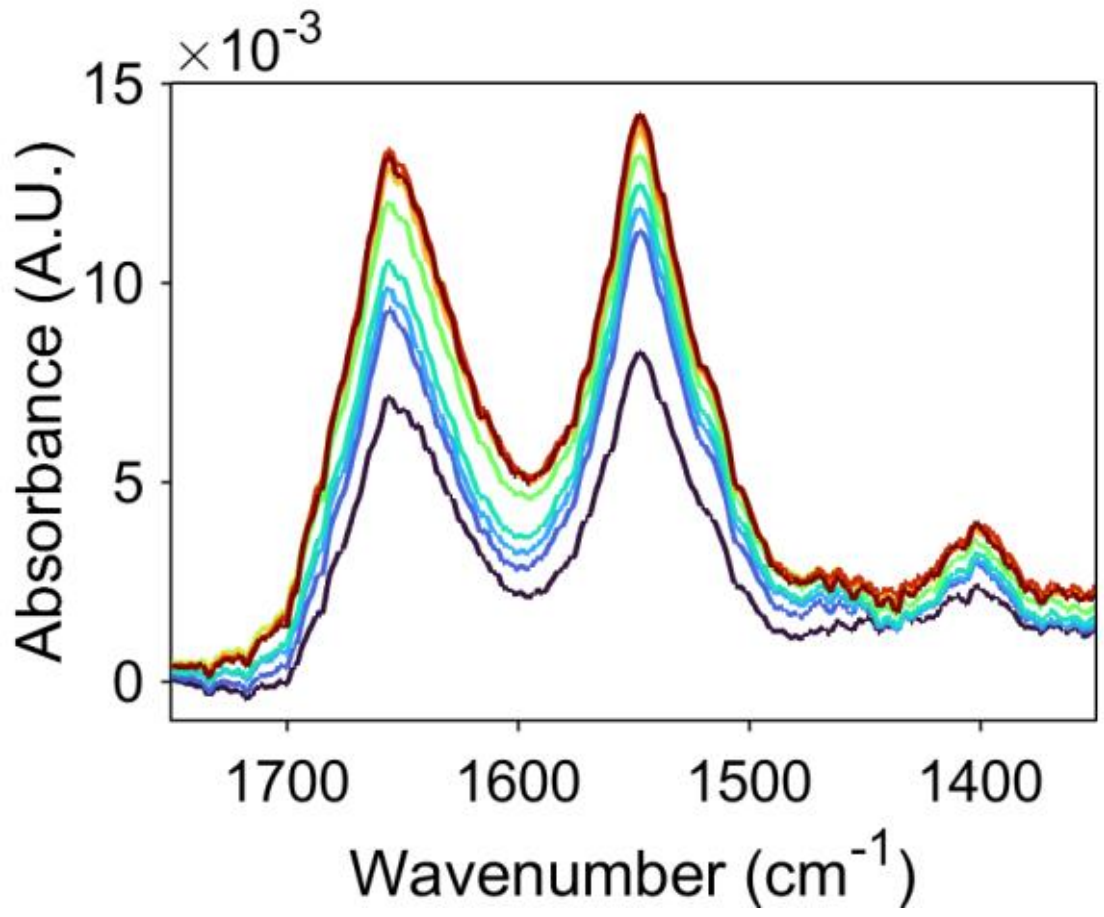

D)

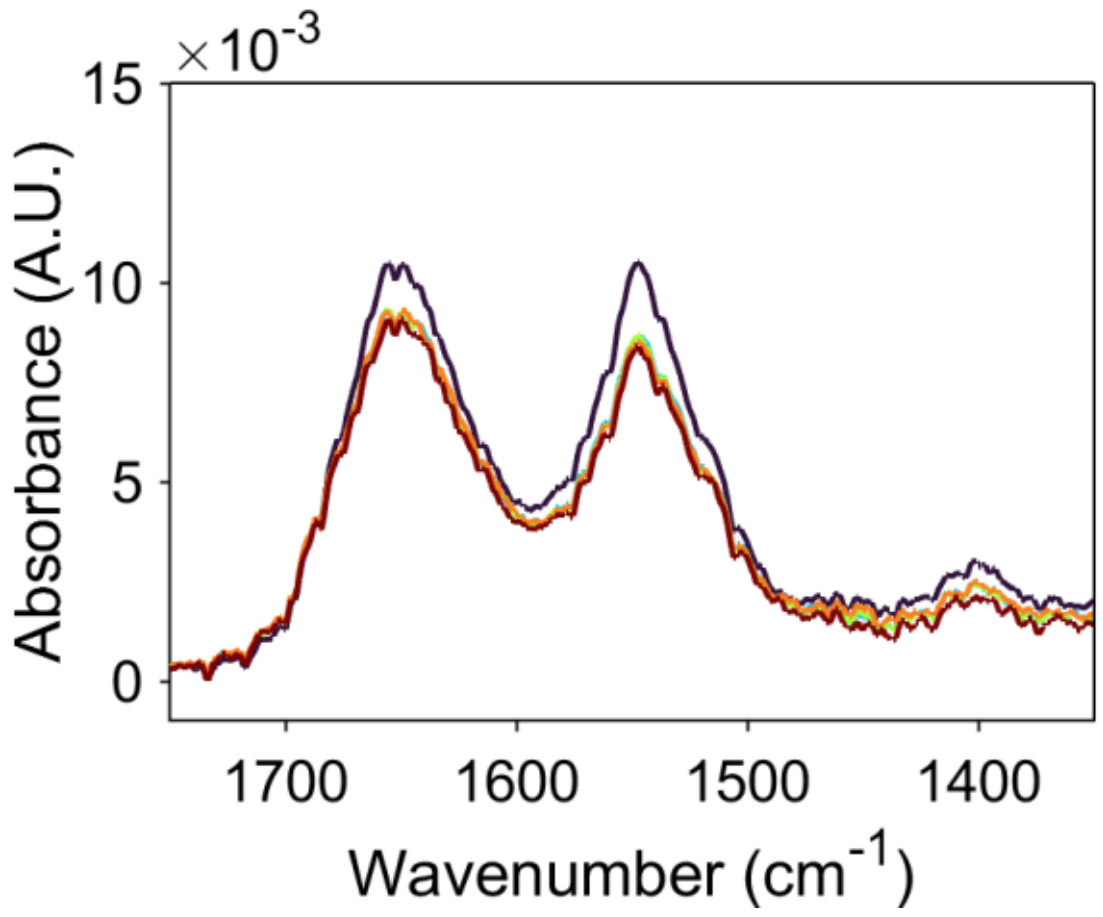

E)

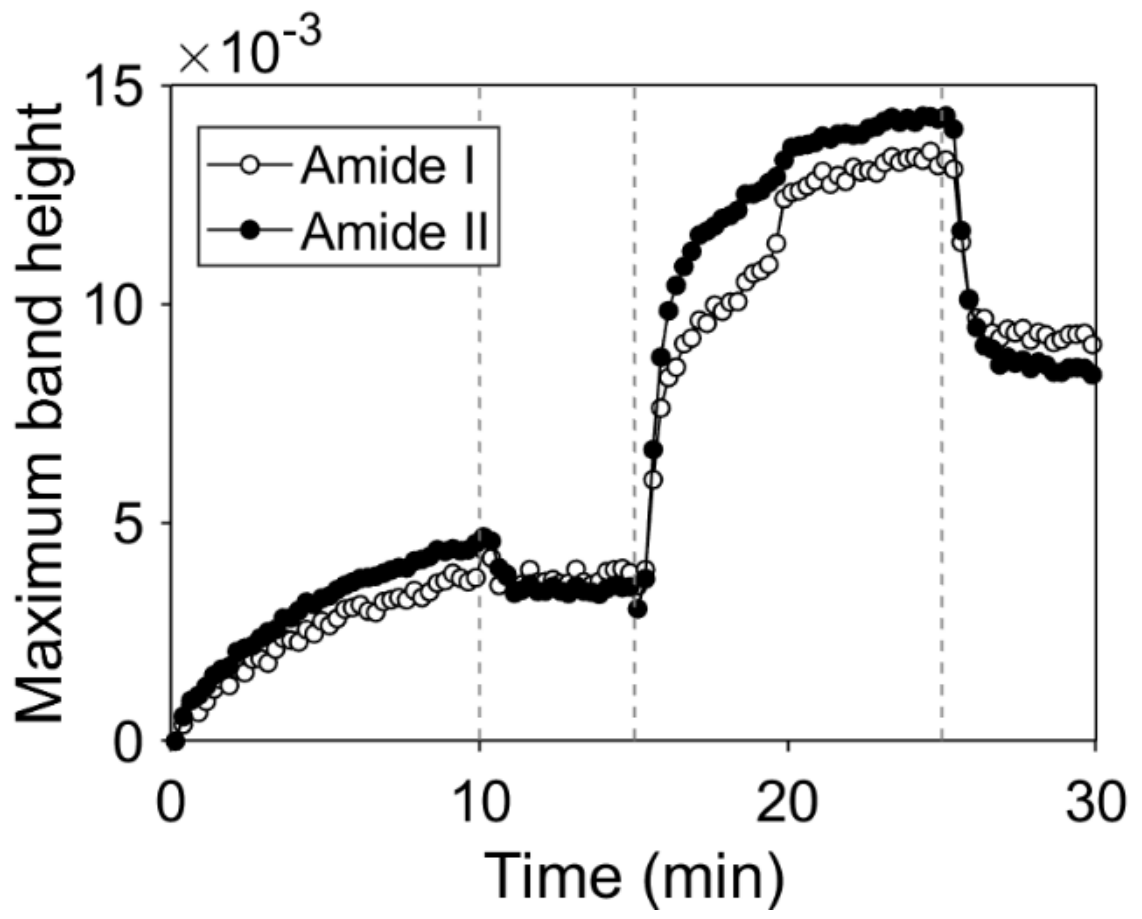

Figure S 31: **Averaged ATR-FTIR spectra of BSA on blank ATR crystal (pH 7).** A) 20 µM protein application. B) Washing with MilliQ. C) 80 µM protein application. D) Washing with MilliQ. E). Maximum band height of amide I (~1650 cm^{-1}, white) and amide II (~1550 cm^{-1}, black) bands plotted against time. Starting spectra are depicted in blue and transform to red. The gray dashed lines indicate solutions swaps. Background spectrum: ATR crystal flushed with MilliQ.

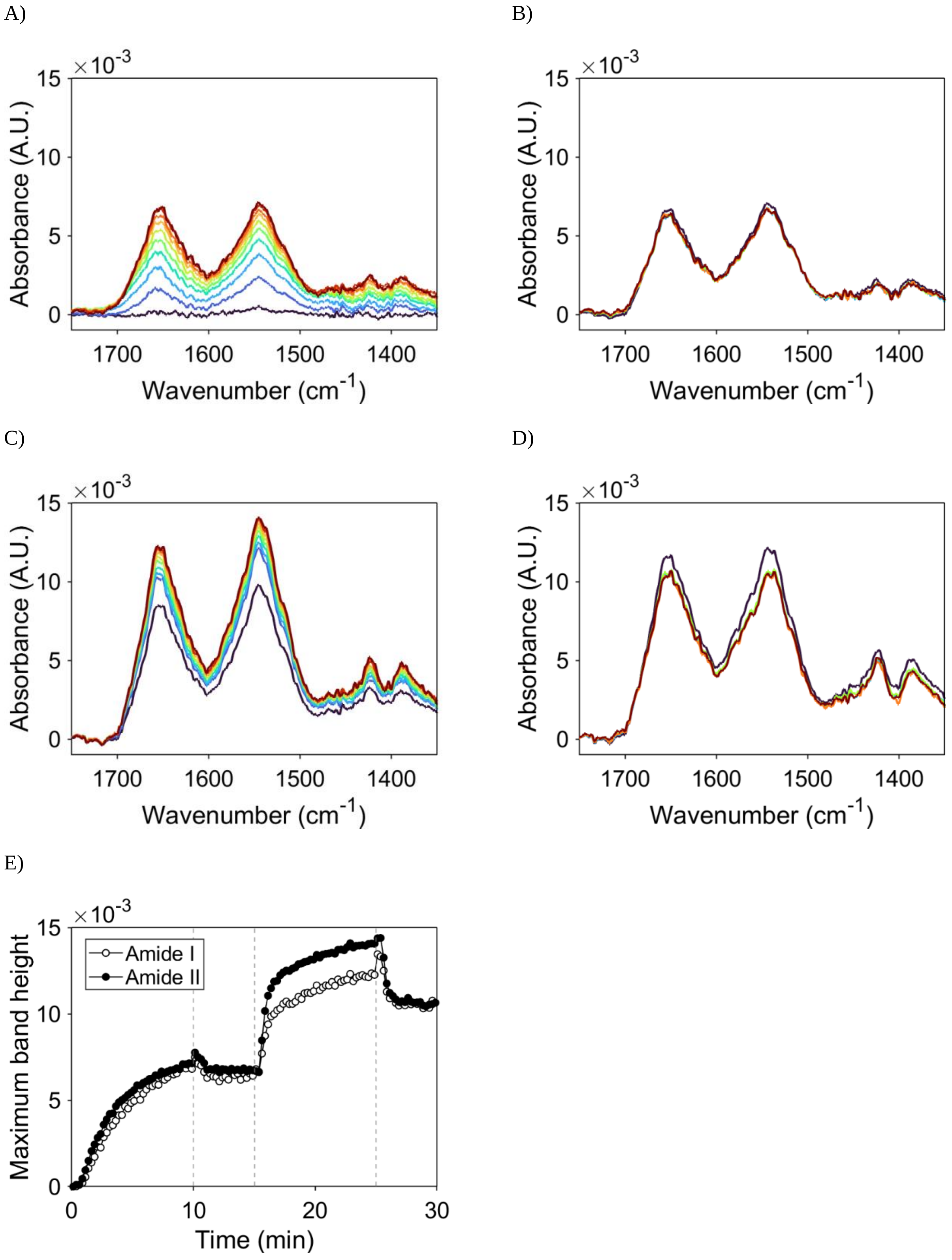

Figure S 32: **Averaged ATR-FTIR spectra of BSA on NU-1000 coated ATR (pH 7).** A) 20 µM protein application. B) Washing with MilliQ. C) 80 µM protein application. D) Washing with MilliQ. E). Maximum band height of amide I (~1650 cm^{-1}, white) and amide II (~1550 cm^{-1}, black) bands plotted against time. Starting spectra are depicted in blue and transform to red. The gray dashed lines indicate solutions swaps. Background spectrum**:** NU-1000 coated ATR crystal flushed with MilliQ.

A)

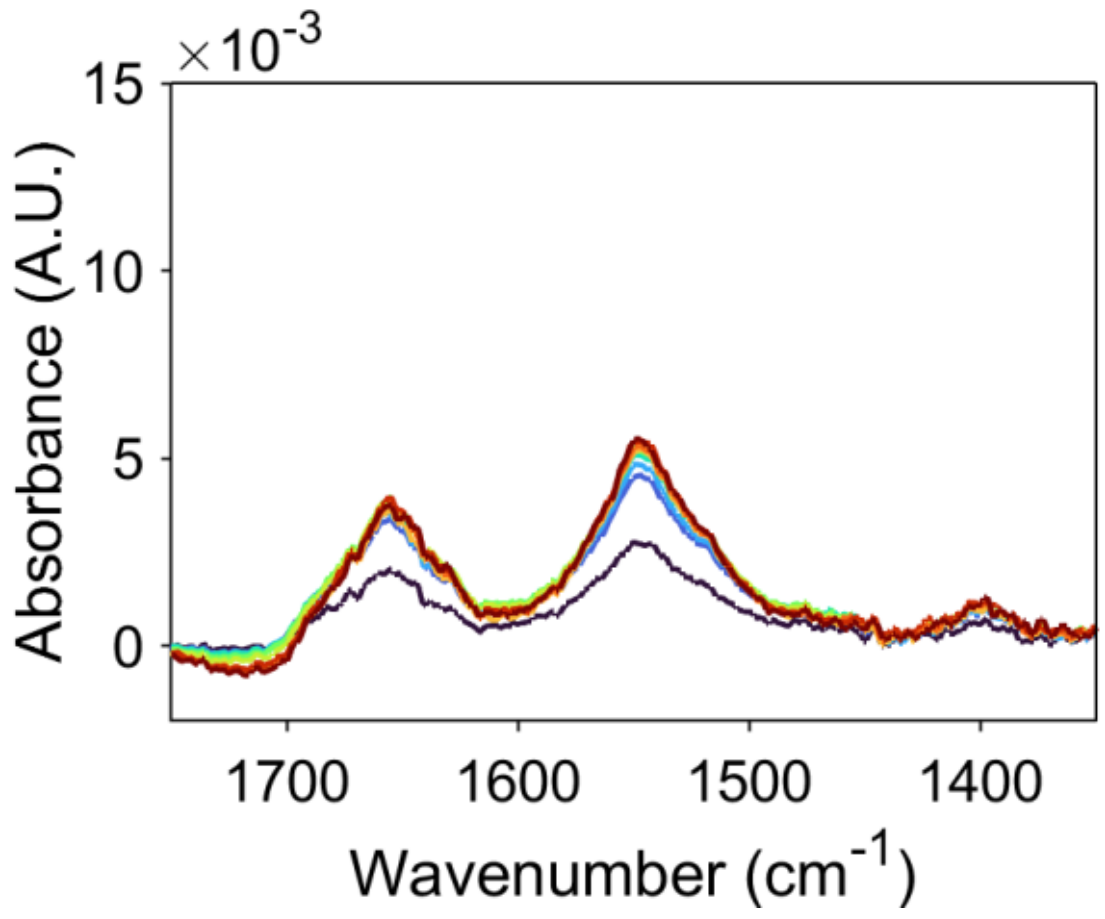

B)

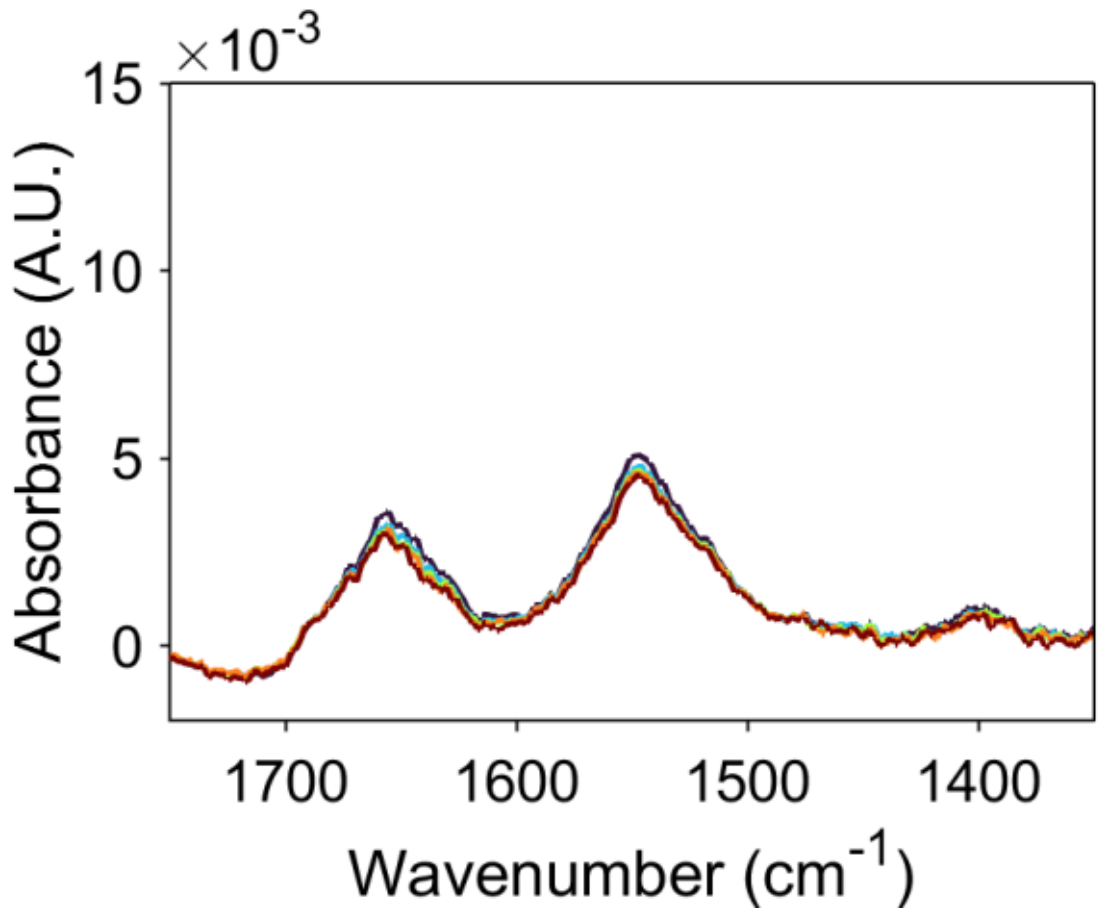

C)

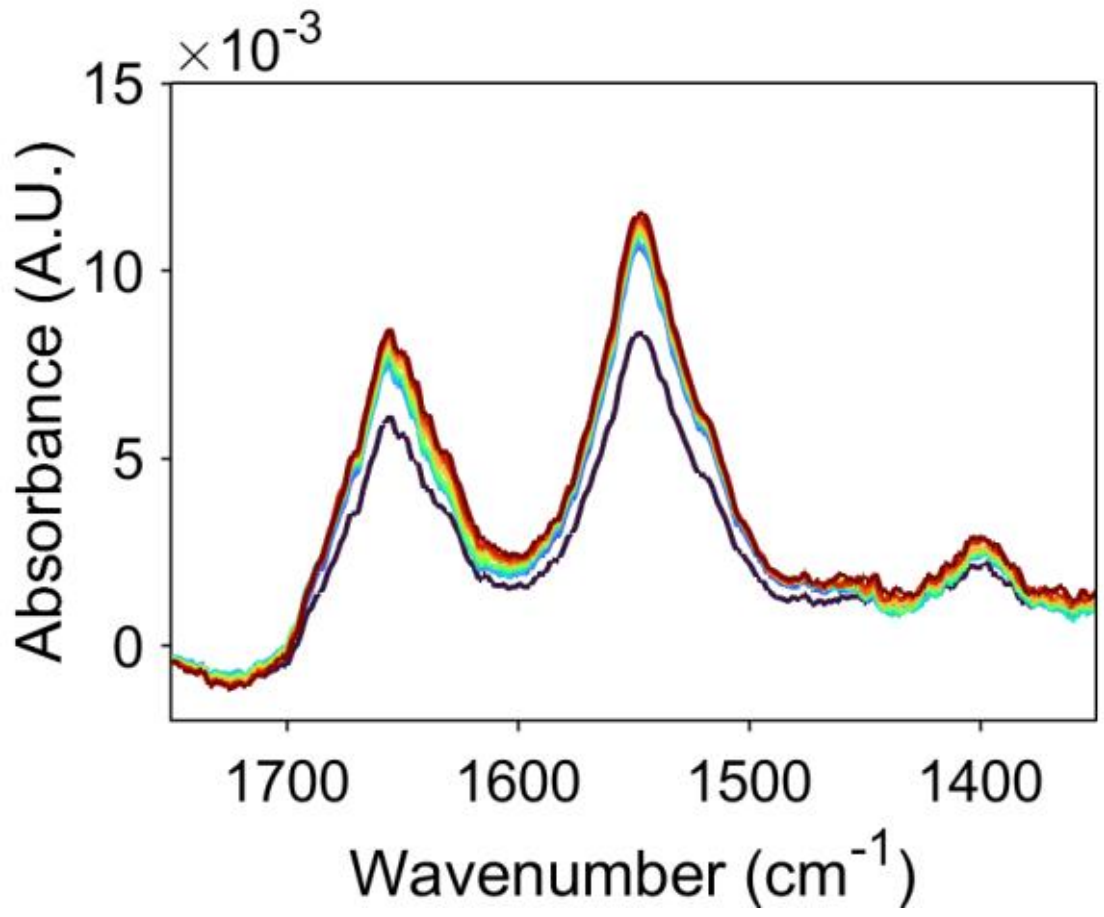

D)

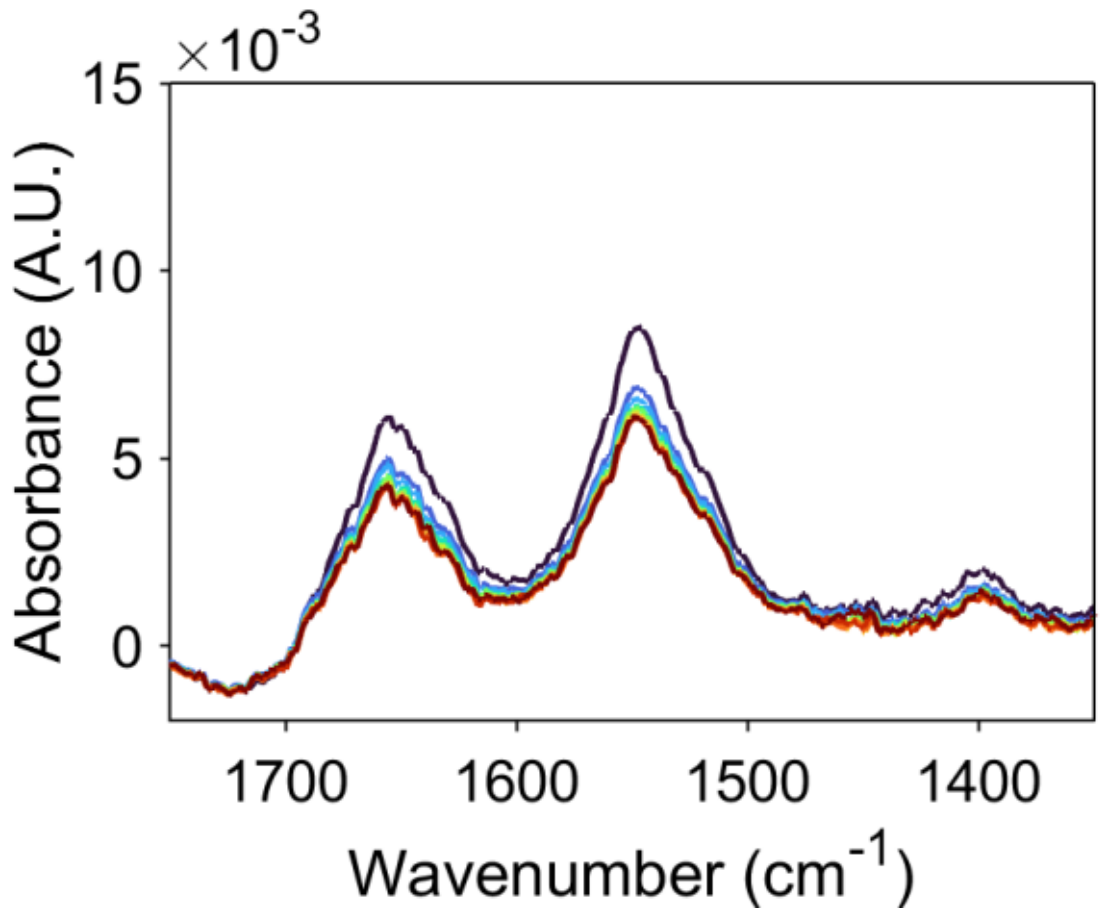

E)

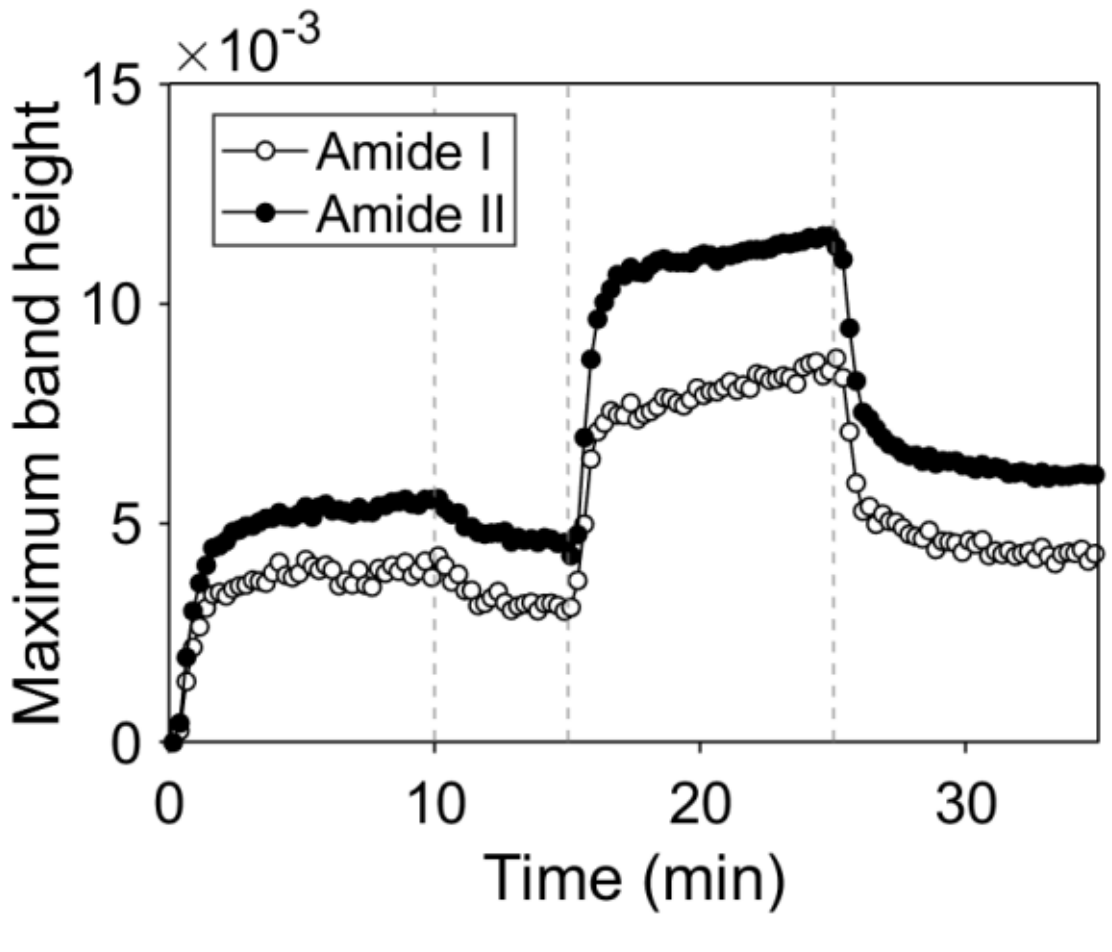

Figure S 33: **Averaged ATR-FTIR spectra of BSA on blank ATR crystal (pH 5.5).** A) 20 µM protein application. B) Washing with MilliQ. C) 80 µM protein application. D) Washing with MilliQ. E). Maximum band height of amide I (~1650 cm^{-1}, white) and amide II (~1550 cm^{-1}, black) bands plotted against time. Starting spectra are depicted in blue and transform to red. The gray dashed lines indicate solutions swaps. Background spectrum**:** blank ATR crystal flushed with MilliQ.

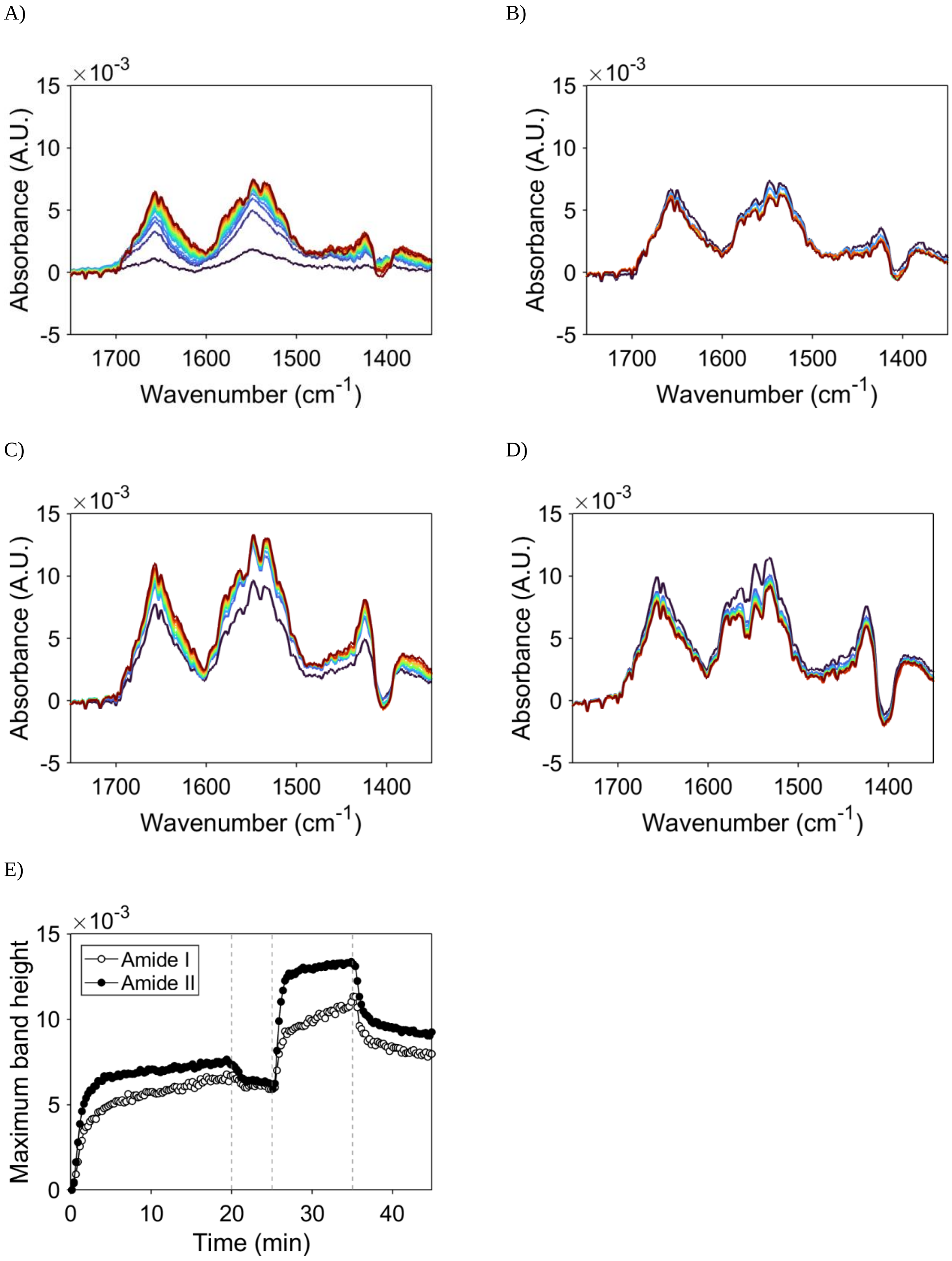

Figure S 34: **Averaged ATR-FTIR spectra of BSA on NU-1000 coated ATR crystal (pH 5.5).** A) 20 µM protein application. B) Washing with MilliQ. C) 80 µM protein application. D) Washing with MilliQ. E). Maximum band height of amide I (~1650 cm^{-1}, white) and amide II (~1550 cm^{-1}, black) bands plotted against time. Starting spectra are depicted in blue and transform to red. The gray dashed lines indicate solutions swaps. Background spectrum: NU-1000 coated ATR crystal flushed with MilliQ.

A)

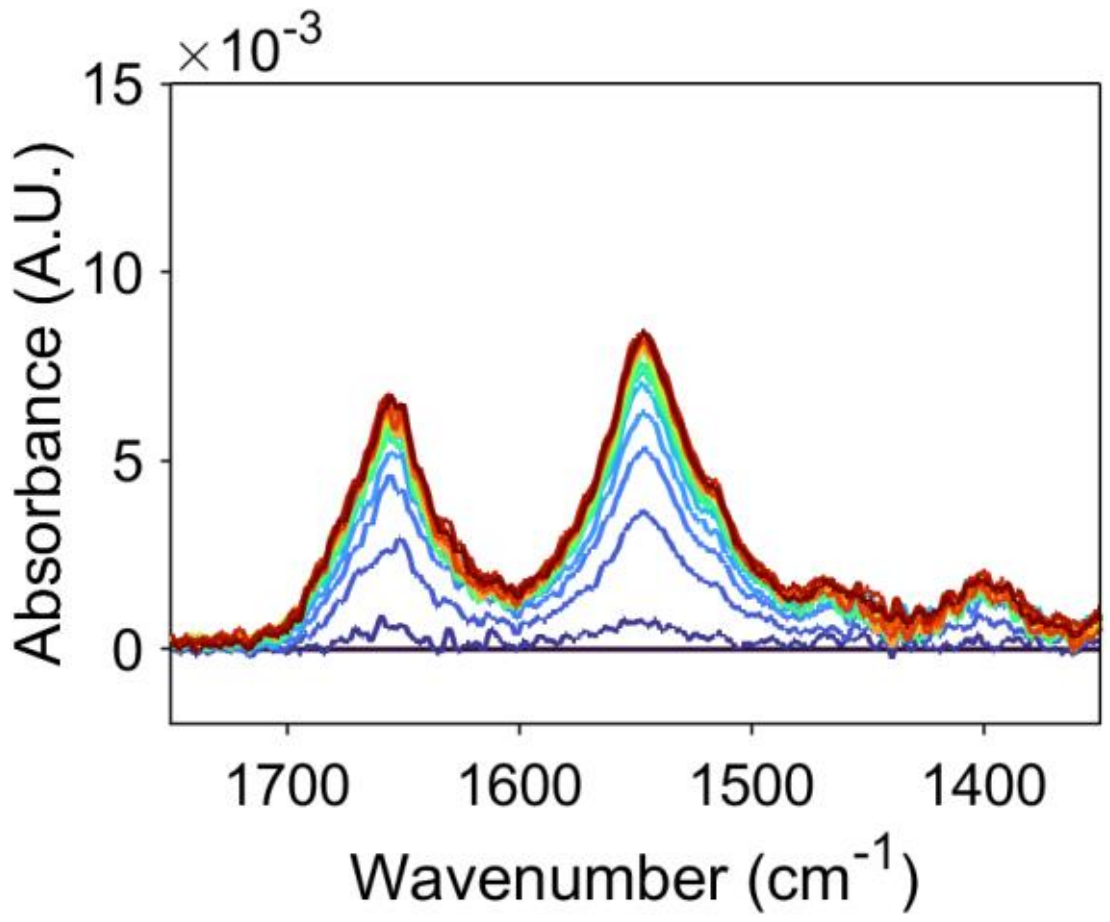

B)

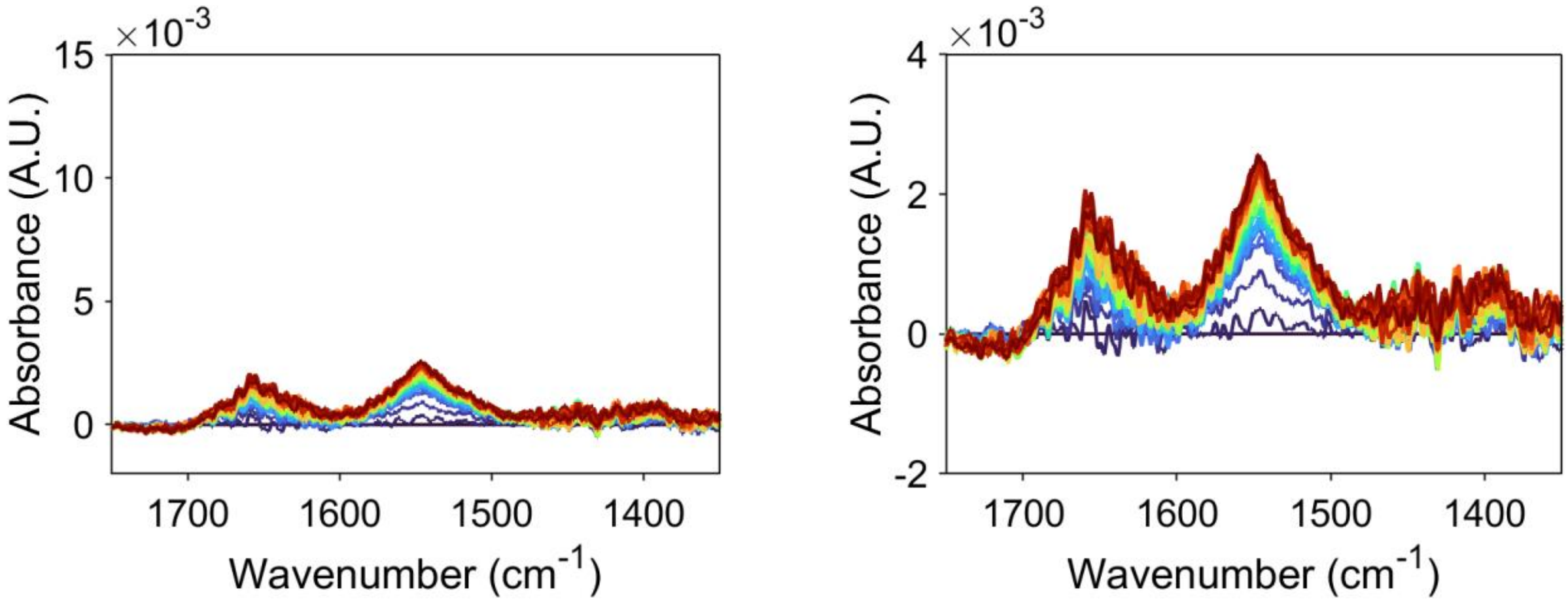

C)

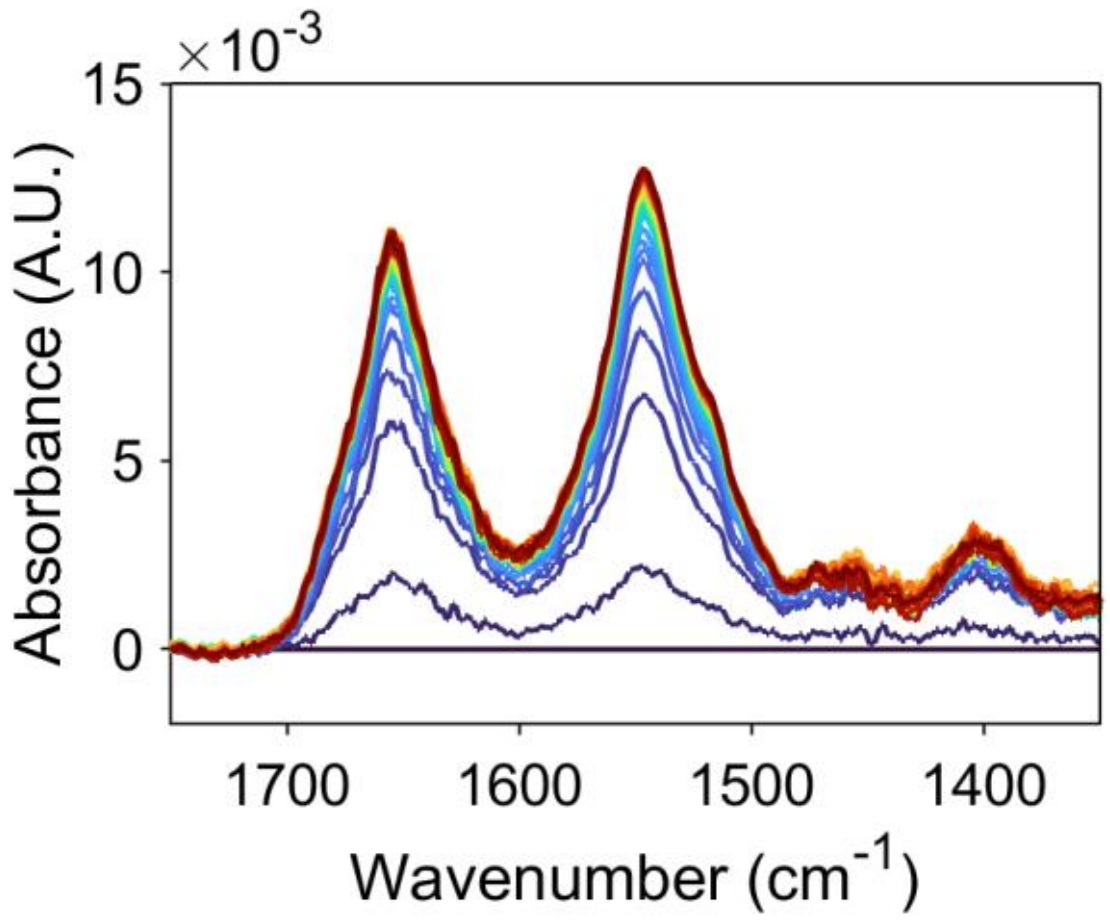

Figure S 35: **ATR-FTIR spectra of 20 µM BSA on blank ATR crystal.** A) In presence of 0.5 % EtOH. B) With heat treatment (25 min at 75°C) before IR spectroscopy. C) In presence of few microliters of NaCl (0.1 M) no formation of salt bridges and BSA penetration are observed. Background spectrum**:** blank ATR crystal flushed with MilliQ.

A)

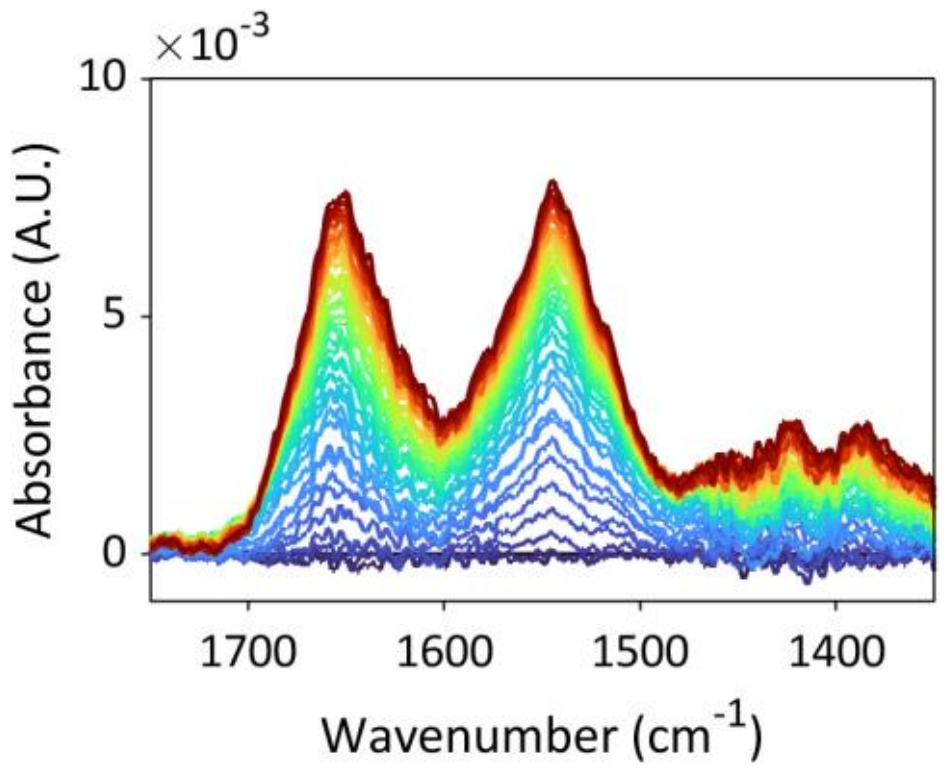

B)

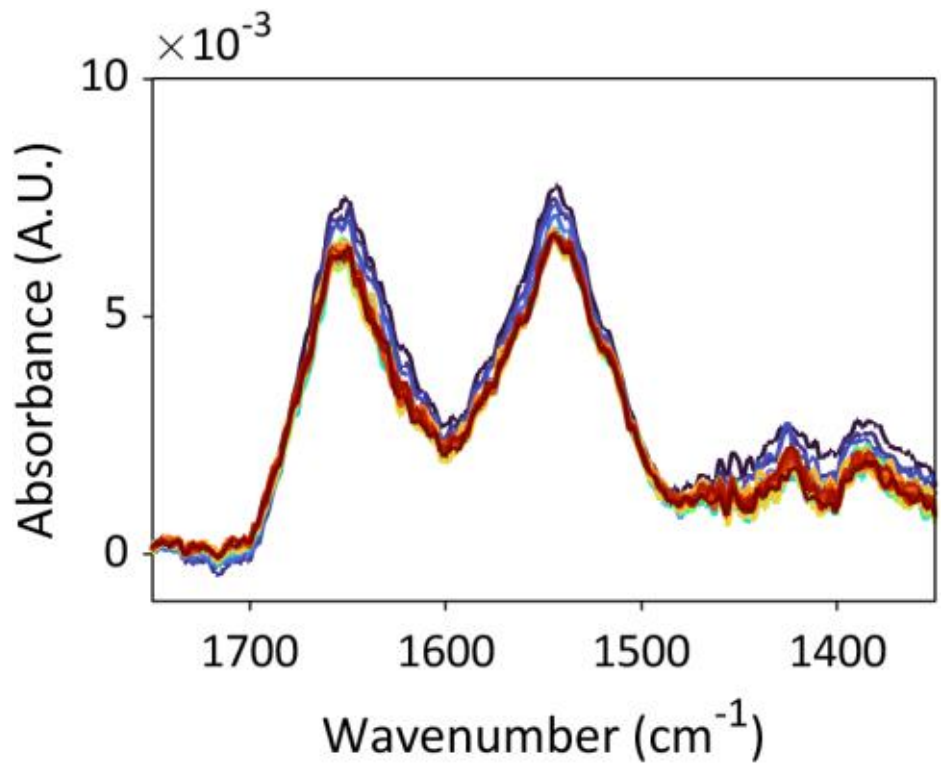

C)

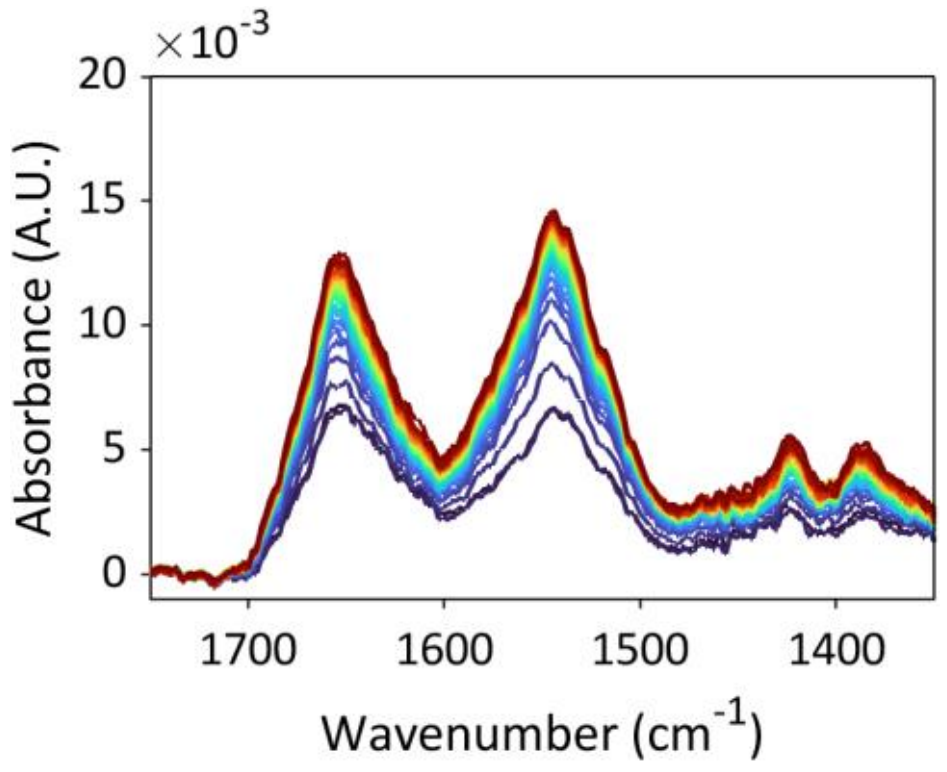

D)

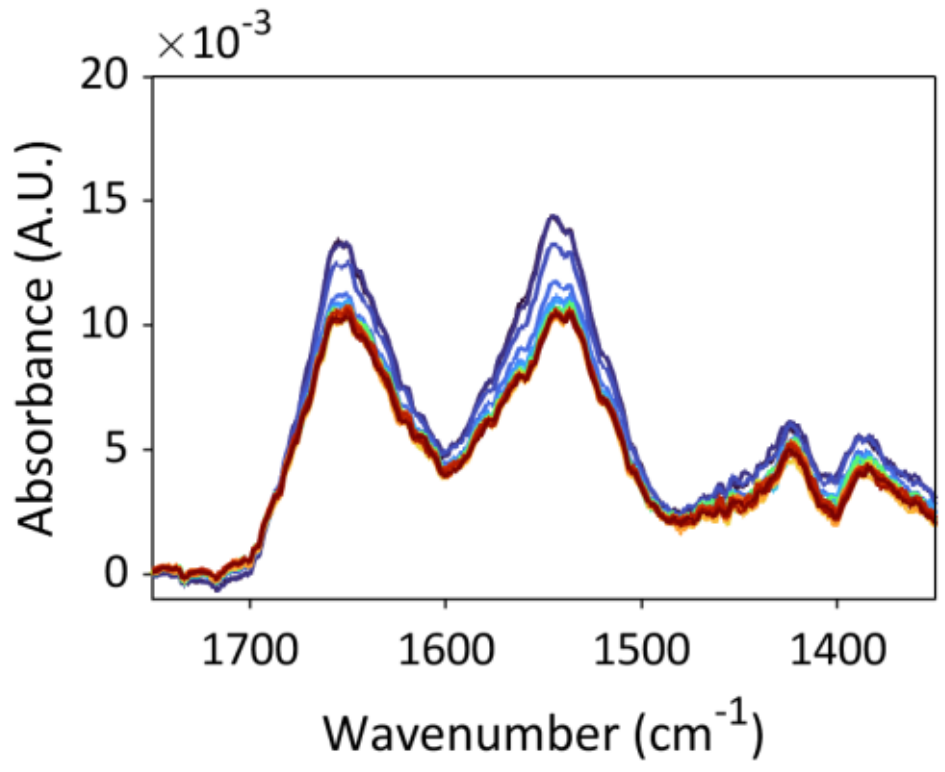

C)

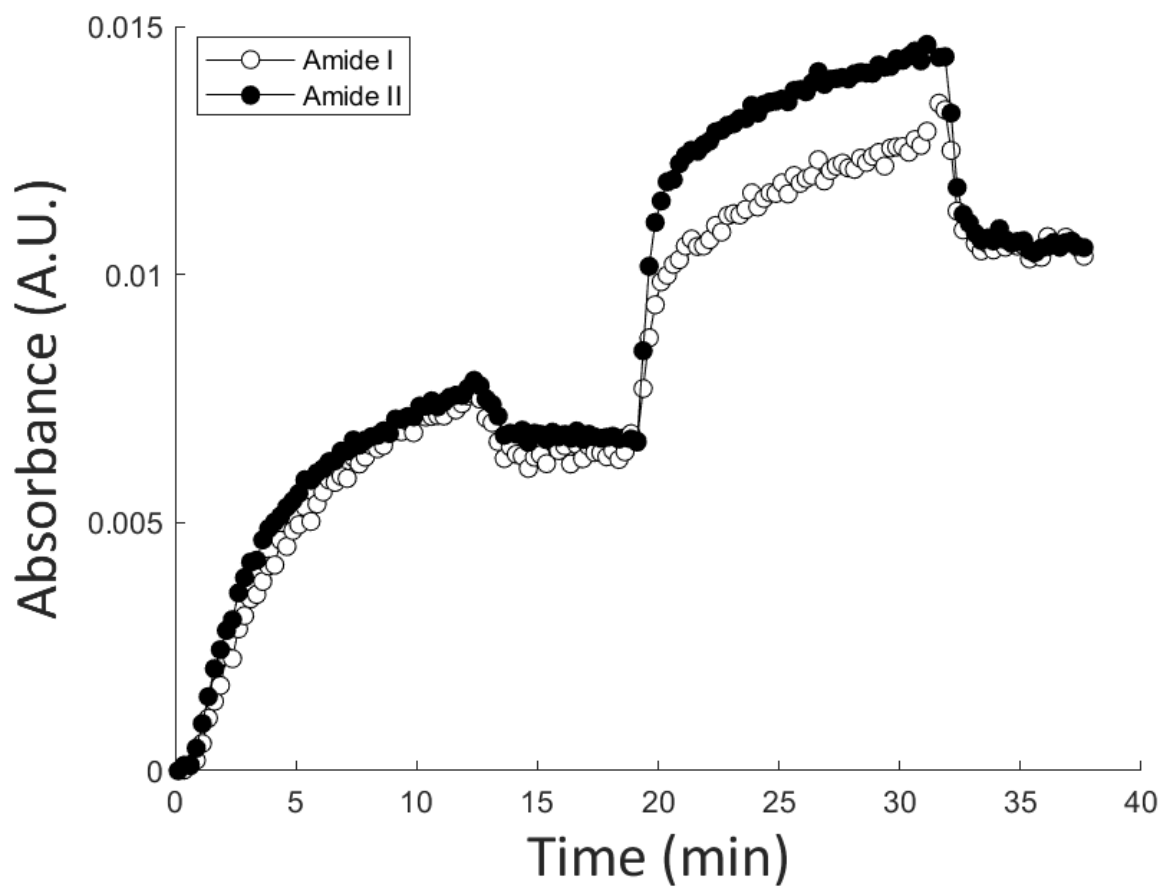

Figure S 36: **ATR-FTIR spectra of 20 µM BSA on NU-1000 coated ATR crystal in presence of few microliters of NaCl (0.1 M).** Background spectrum**:** blank ATR crystal flushed with MilliQ. A) 20 µM protein application. B) Washing with MilliQ. C) 80 µM protein application. D) Washing with MilliQ. E). Maximum band height of amide I (~1650 cm^{-1}, white) and amide II (~1550 cm^{-1}, black) bands plotted against time. Starting spectra are depicted in blue and transform to red. Background spectrum**:** NU-1000 coated ATR crystal flushed with MilliQ.

S11: Enzymatic reactivity

Table S 10: Overview of the amounts of immobilized enzymes onto NU-1000.

Enzyme type<br>Immobilization time	#Immobilized Enzyme<br>(nmol/mg NU-1000)	#Immobilized Enzyme<br>(µg/mg NU-1000)
Cyt c, 2 h	0.64	8.0
Cyt c, 24 h	0.27*	3.4*
BSA, 2 h	0.19	13
BSA, 24 h	1.90	124

*Note that this value is not reliable due to Cyt c denaturation over 24 h.

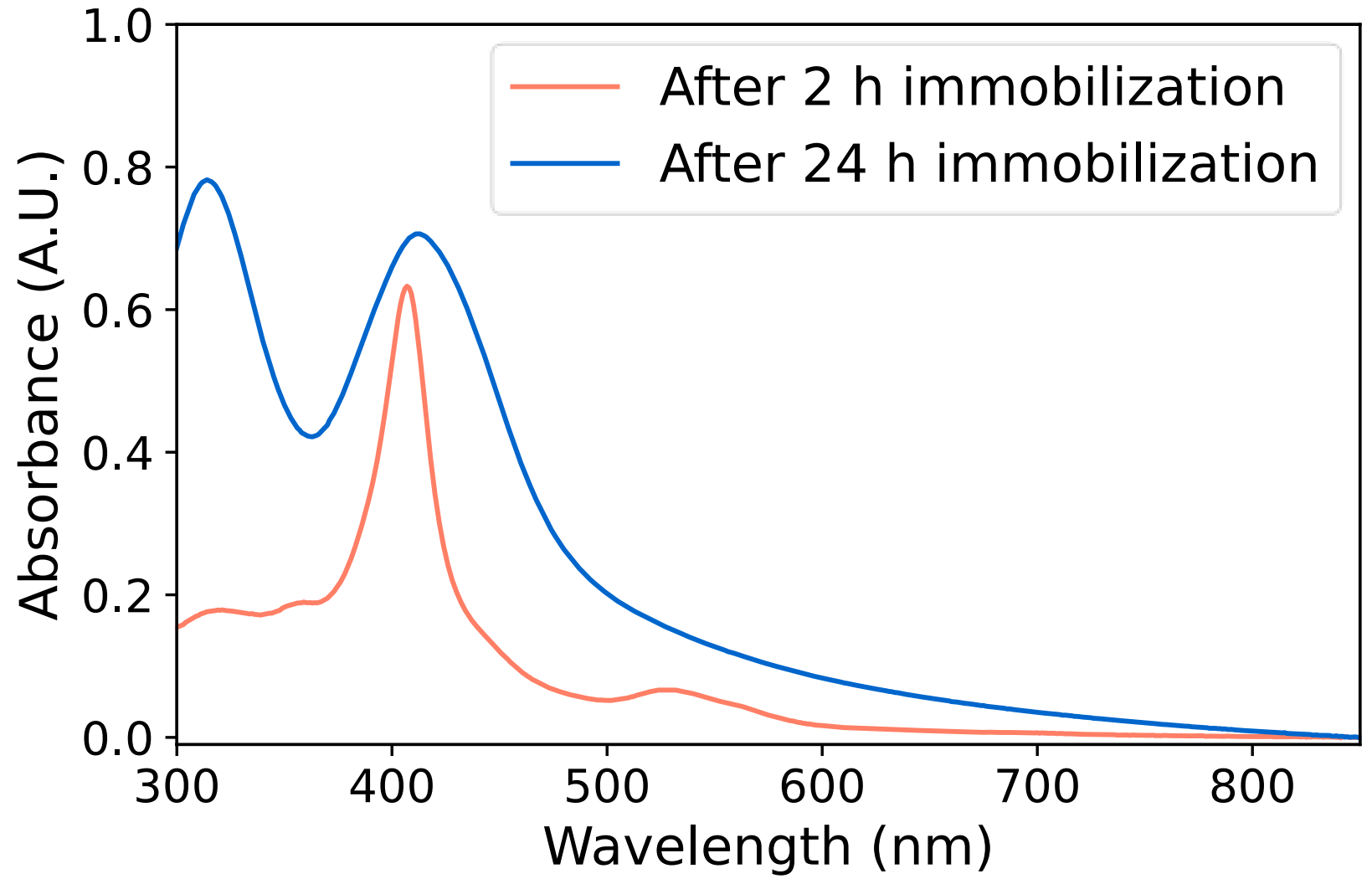

Figure S 37: UV-vis spectra of Cyt c supernatant after 2 and 24 h of immobilization. Broadening of the Soret band at 415 nm after 24 h of immobilization point towards denaturation.

Table S 11: Overview of the initial reaction rates for the hydrolysis of para-nitrophenyl acetate by indicated catalysts.

Catalyst<br>(+ immobilization time)	Reaction rate<br>($\times 10^{-5}$ s^{-1})	Normalized reaction rate<br>($\times 10^{-5}$ s^{-1} mol^{-1})
Blank	4.2	-
NU-1000	37.6	-
Cyt c	111.1	-
Cyt c@NU-1000 (2 h)	371.6	1376.3

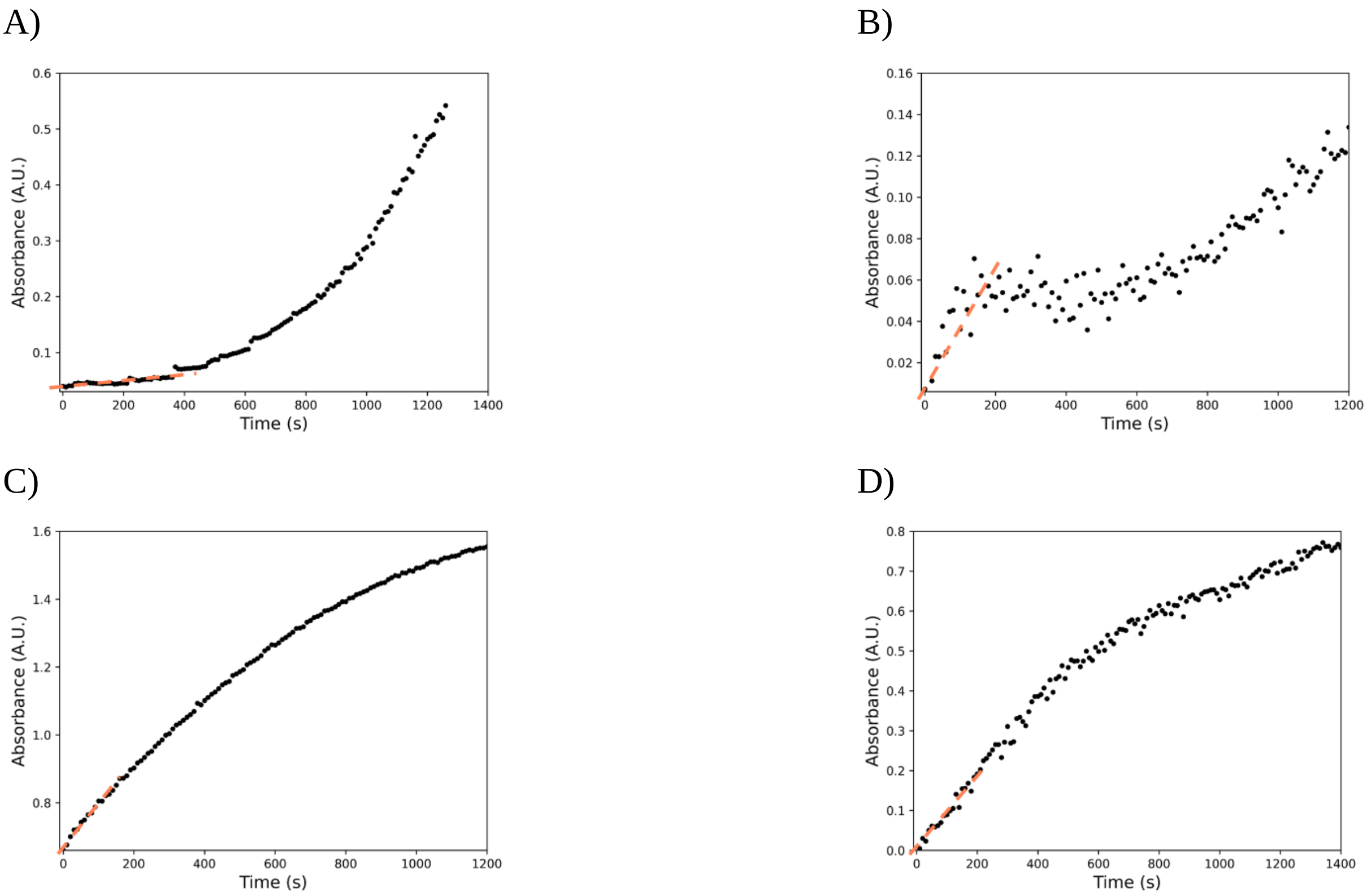

Figure S 38: **Absorbance at 420 nm over time and initial rate (orange dashed line) for the oxidation of Pyrogallol.** A) Control reaction in Tris buffer alone. B) Control reaction catalyzed by NU-1000. C) Catalyzed by Cyt c. D) Catalyzed by Cyt c@NU-1000.

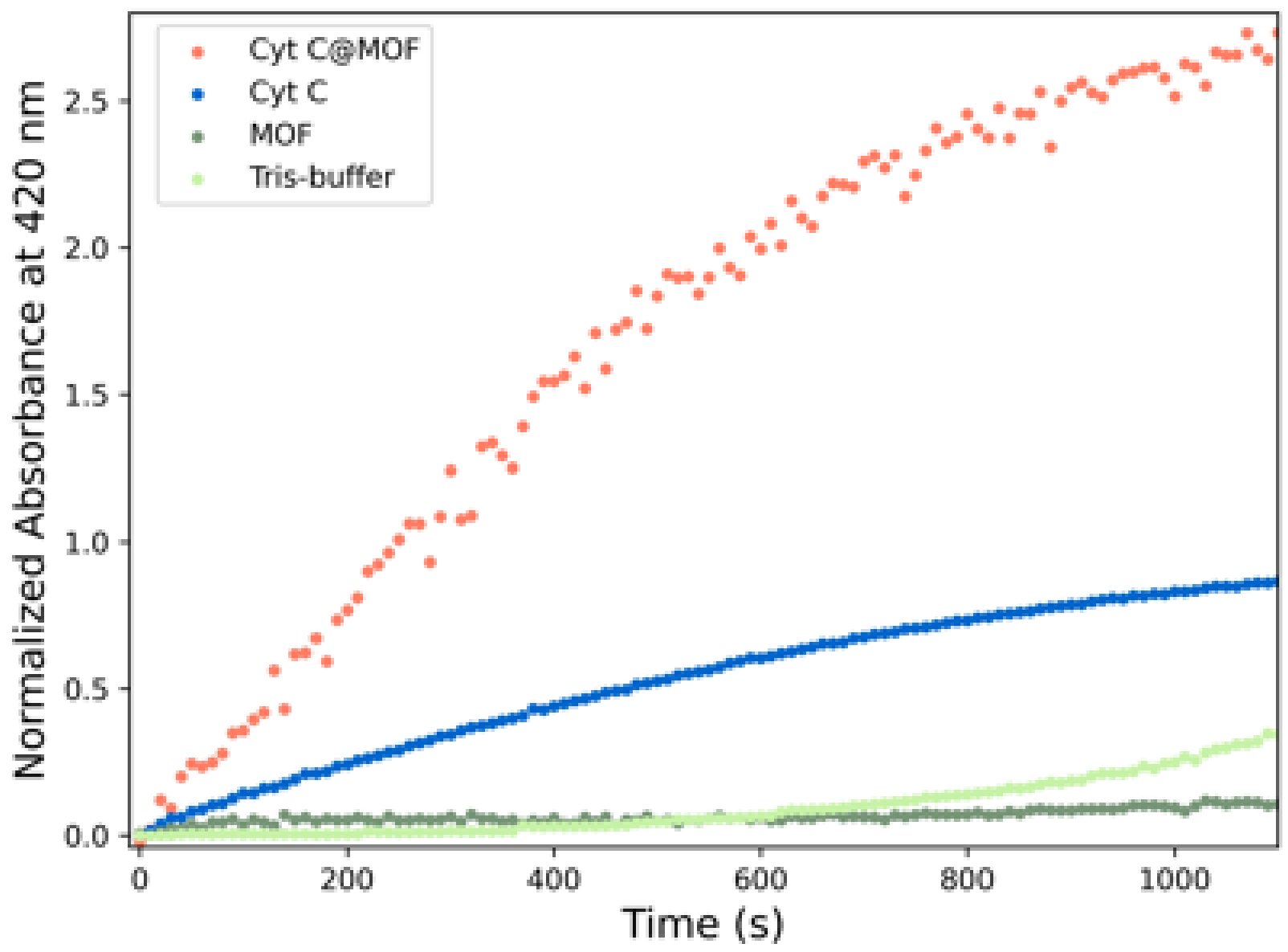

Figure S 39: Comparison of normalized absorbance at 420 nm over time for the oxidation of pyrogallol.

Table S 12: Overview of the initial reaction rates for the hydrolysis of para-nitrophenyl acetate by indicated catalysts.

**Catalyst (+ immobilization time)**	**Reaction rate ($\times10^{-5}$ s^{-1})**	**Normalized reaction rate ($\times10^{-5}$ s^{-1} mol^{-1})**
Blank	0.3	-
NU-1000	3.6	-
BSA	2.5	-
BSA@NU-1000 (2 h)	2.1	11.1
BSA	13.9	-
BSA (24 h)	3.4	1.8

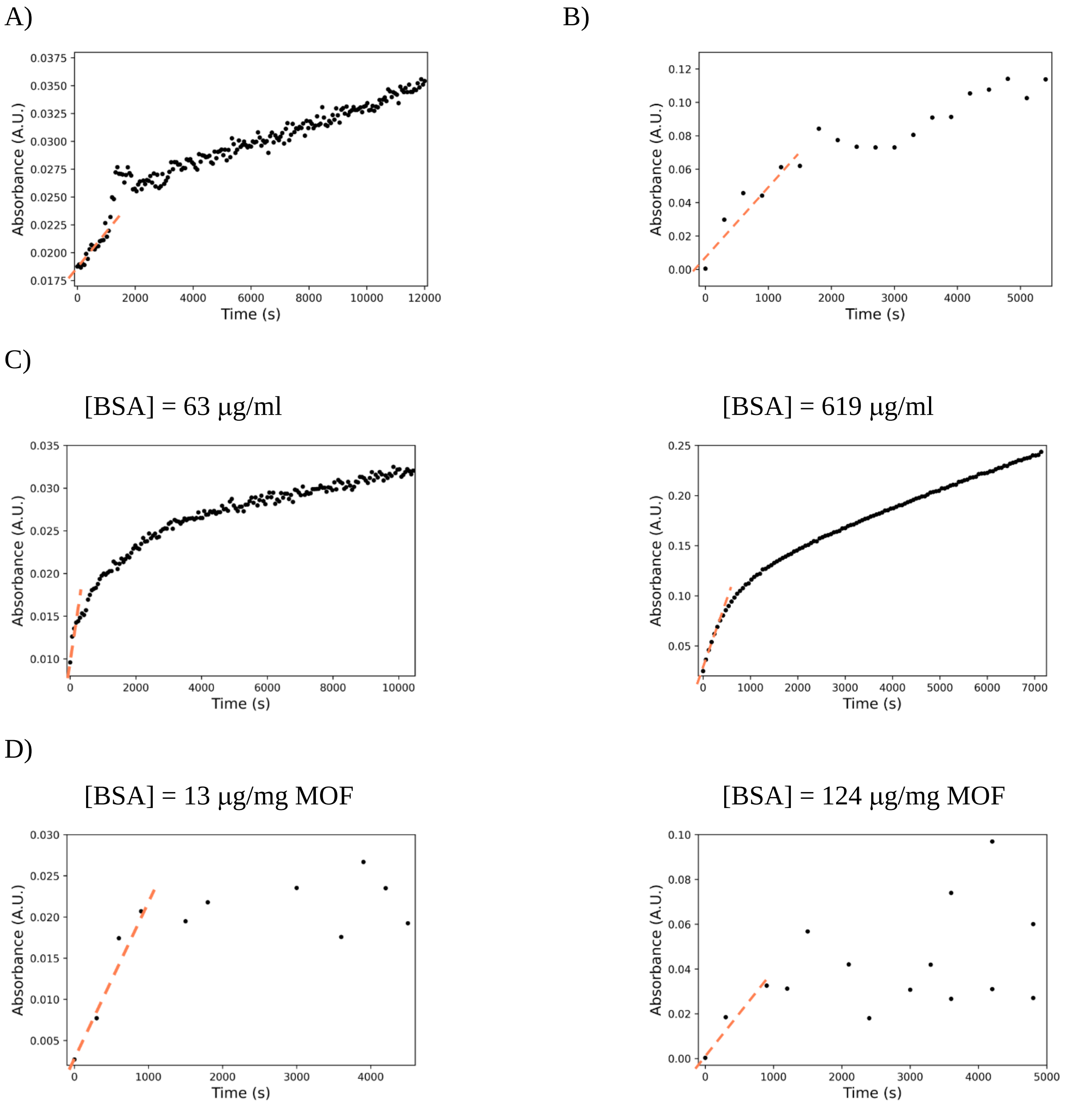

Figure S 40: **Absorbance at 400 nm over time and initial rate (orange dashed line) for the hydrolysis of pNPA (65 µM).** A) Control reaction in PBS alone. B) Control reaction catalyzed by NU-1000. C) Catalyzed by BSA. D) Catalyzed by BSA@NU-1000.

References

(1) Kelly, H. R.; Sreekumar, S.; Manee, V.; Cuomo, A. E.; Newhouse, T. R.; Batista, V. S.; Buono, F. Ligand-Based Principal Component Analysis Followed by Ridge Regression: Application to an Asymmetric Negishi Reaction. *ACS Catal.* **2024**, *14* (7), 5027–5038. DOI: https://doi.org/10.1021/acscatal.3c06230.

(2) Grant, B. J.; Rodrigues, A. P. C.; ElSawy, K. M.; McCammon, J. A.; Caves, L. S. D. Bio3d: an R package for the comparative analysis of protein structures. *Bioinformatics* **2006**, *22* (21), 2695–2696. DOI: https://doi.org/10.1093/bioinformatics/btl461.

(3) Wright, E. S. Using DECIPHER v2.0 to Analyze Big Biological Sequence Data in R. *The R Journal* **2016**, *8* (1), 352–359. DOI: https://doi.org/10.32614/RJ-2016-025.

(4) Osorio, D. a. R.-V., Paola and Torres, Rodrigo. Peptides: A Package for Data Mining of Antimicrobial Peptides. *The R journal* **2015**, *7* (1), 4–14. DOI: https://doi.org/10.32614/RJ-2015-001.

(5) Xiao, N.; Cao, D.-S.; Zhu, M.-F.; Xu, Q.-S. protr/ProtrWeb: R package and web server for generating various numerical representation schemes of protein sequences. *Bioinformatics* **2015**, *31* (11), 1857–1859. DOI: https://doi.org/10.1093/bioinformatics/btv042.

(6) van den Boogaart, K. G.; Tolosana-Delgado, R. "compositions": A unified R package to analyze compositional data. *Computers & Geosciences* **2008**, *34* (4), 320–338. DOI: https://doi.org/10.1016/j.cageo.2006.11.017.

(7) *R: A Language and Environment for Statistical Computing*; R Core Team: Vienna, Austria, 2021. https://www.R-project.org/.

(8) Loosen, A.; de Azambuja, F.; Parac-Vogt, T. N. Which factors govern the adsorption of peptides to Zr(iv)-based metal–organic frameworks? *Mater. Adv.* **2022**, *3* (5), 2475–2487. DOI: https://doi.org/10.1039/D1MA01027A.

(9) Rouquerol, J.; Rouquerol, F.; Sing, K. S. W. *Absorption by Powders and Porous Solids*; Academic Press, 2013.

(10) Harrick, N. J.; Carlson, A. I. Internal Reflection Spectroscopy: Validity of Effective Thickness Equations. *Appl. Opt.* **1971**, *10*, 19–23. DOI: https://doi.org/10.1364/AO.10.000019.

(11) Li, P.; Modica, Justin A.; Howarth, Ashlee J.; Vargas L, E.; Moghadam, Peyman Z.; Snurr, Randall Q.; Mrksich, M.; Hupp, Joseph T.; Farha, Omar K. Toward Design Rules for Enzyme Immobilization in Hierarchical Mesoporous Metal-Organic Frameworks. *Chem* **2016**, *1* (1), 154–169. DOI: https://doi.org/10.1016/j.chempr.2016.05.001.

(12) Titze, T.; Lauerer, A.; Heinke, L.; Chmelik, C.; Zimmermann, N. E. R.; Keil, F. J.; Ruthven, D. M.; Kärger, J. Transport in Nanoporous Materials Including MOFs: The Applicability of Fick's Laws. *Angewandte Chemie International Edition* **2015**, *54* (48), 14580–14583. DOI: https://doi.org/10.1002/anie.201506954.

(13) Kärger, J.; Ruthven, D. M.; Theodorou, D. N. *Diffusion in Nanoporous Materials*; Wiley-VCH, 2012.

(14) Dvoyashkin, M.; Chmelik, C.; Nonnen, T.; Hermann, R.; Russina, M.; Gläser, R. Probing Water Diffusion Inside Crystals of AlPO-5 by PFG NMR and IRM for Heat Storage Applications. *Chem. Ing. Tech.* **2023**, *95* (11), 1748–1757. DOI: https://doi.org/10.1002/cite.202300072.

(15) Akpinar, I.; Wang, X.; Fahy, K.; Sha, F.; Yang, S.; Kwon, T.-w.; Das, P. J.; Islamoglu, T.; Farha, O. K.; Stoddart, J. F. Biomimetic Mineralization of Large Enzymes Utilizing a Stable Zirconium-Based Metal-Organic Frameworks. *J. Am. Chem. Soc.* **2024**, *146* (8), 5108–5117. DOI: https://doi.org/10.1021/jacs.3c07785.

(16) Greifenstein, R.; Ballweg, T.; Hashem, T.; Gottwald, E.; Achauer, D.; Kirschhöfer, F.; Nusser, M.; Brenner-Weiß, G.; Sedghamiz, E.; Wenzel, W.; et al. MOF-Hosted Enzymes for Continuous Flow Catalysis in Aqueous and Organic Solvents. *Angew. Chem. Int. Ed.* **2022**, *61* (18), e202117144. DOI: https://doi.org/10.1002/anie.202117144.

(17) Lamy, H.; Bullier-Marchandin, E.; Pointel, C.; Echalard, A.; Ladam, G. D.; Lutzweiler, G. Kinetic Study of the Esterase-like Activity of Albumin following Condensation by Macromolecular Crowding. *Biomacromolecules* **2024**, *25* (5), 2803–2813. DOI: https://doi.org/10.1021/acs.biomac.3c01431.

(18) Sha, F.; Chen, Y.; Drout, R. J.; Idrees, K. B.; Zhang, X.; Farha, O. K. Stabilization of an enzyme cytochrome c in a metal-organic framework against denaturing organic solvents. *iScience* **2021**, *24* (6). DOI: https://doi.org/10.1016/j.isci.2021.102641.

(19) Swinnen, S.; Lox, M.; de Azambuja, F.; Parac-Vogt, T. N. UiO-66 MOF Nanozymes as Affinity Columns for Proteomics Applications. *ACS App. Nano Mater.* **2025**, *8* (7), 3536–3544. DOI: https://doi.org/10.1021/acsanm.4c07008.
